# Modulation of the Naked‐Eye and Fluorescence Color of a Protonated Boron‐Doped Thiazolothiazole by Anion‐Dependent Hydrogen Bonding

**DOI:** 10.1002/chem.202201398

**Published:** 2022-07-13

**Authors:** Stephan Hagspiel, Felipe Fantuzzi, Merle Arrowsmith, Annalena Gärtner, Maximilian Fest, Jonas Weiser, Bernd Engels, Holger Helten, Holger Braunschweig

**Affiliations:** ^1^ Institute for Inorganic Chemistry Julius-Maximilians-Universität Würzburg Am Hubland 97074 Würzburg Germany; ^2^ Institute for Sustainable Chemistry & Catalysis with Boron Julius-Maximilians-Universität Würzburg Am Hubland 97074 Würzburg Germany; ^3^ Institute for Physical and Theoretical Chemistry Julius-Maximilians-Universität Würzburg Emil-Fischer-Str. 42 97074 Würzburg Germany; ^4^ Current address: School of Physical Sciences Ingram Building University of Kent Park Wood Rd CT2 7NH Canterbury UK

**Keywords:** hydrogen bonding, intramolecular charge transfer, thiazaborolothiazaborole, visible and fluorescence color modulation

## Abstract

The reaction of a cyclic alkyl(amino)carbene (CAAC)‐stabilized thiazaborolo[5,4‐*d*]thiazaborole (TzbTzb) with strong Brønsted acids, such as HCl, HOTf (Tf=O_2_SCF_3_) and [H(OEt_2_)_2_][BAr^F^_4_] (Ar^F^=3,5‐(CF_3_)_2_C_6_H_3_), results in the protonation of both TzbTzb nitrogen atoms. In each case X‐ray crystallographic data show coordination of the counteranions (Cl^−^, OTf^−^, BAr^F^_4_^−^) or solvent molecules (OEt_2_) to the doubly protonated fused heterocycle via hydrogen‐bonding interactions, the strength of which strongly influences the ^1^H NMR shift of the N*H* protons, enabling tuning of both the visible (yellow to red) and fluorescence (green to red) colors of these salts. DFT calculations reveal that the hydrogen bonding of the counteranion or solvent to the protonated nitrogen centers affects the intramolecular TzbTzb‐to‐CAAC charge transfer character involved in the S_0_→S_1_ transition, ultimately enabling fine‐tuning of their absorption and emission spectral features.

## Graphical abstract

**The visible and fluorescence colors** of a doubly protonated boron‐doped thiazolothiazole can be modulated by tuning its hydrogen‐bonding interactions with its counteranions or donor solvent molecules (Cl^−^, O_3_SCF_3_^−^, B(3,5‐(CF_3_)_2_C_6_H_3_)_4_^−^, OEt_2_). DFT calculations show that these affect the relative energies of the π orbitals involved in the S_0_→S_1_ transition, thus enabling fine‐tuning of their absorption and emission spectral features.

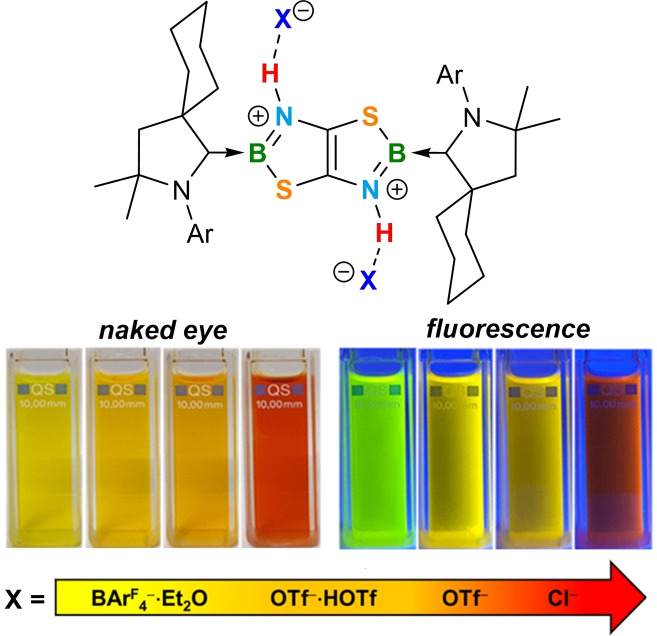

## Introduction

Small molecules capable of displaying a range of visible or fluorescence colors in response to external analytes are particularly sought‐after as sensors for applications in water pollution monitoring and biosensing.[^1^, ^2^] Such colorimetric probes work either by an on‐off fluorescence switch or a chromatic shift of their absorption or emission wavelength as they either bind to or undergo a chemical reaction with the contaminant or biological target of interest. As anions are ubiquitous in biological systems and aqueous environments, the development of new anion sensors remains an area of high research interest, in which heterocyclic chromophores play an important part.[^3^, ^4^, ^5^, ^6^, ^7^]

The highly electron‐deficient thiazolo[5,4‐*d*]thiazole (TzTz) building block, which is comprised of two 1,3‐thiazole units fused at the C−C bond, has generated increasing interest in small‐molecule and materials chemistry for its highly tunable optoelectronic properties.[^8^, ^9^, ^10^] Their light‐harvesting properties make TzTz‐based materials excellent candidates for solar cell applications[^11^, ^12^, ^13^] and photocatalysis.[^14^, ^15^, ^16^] Furthermore, TzTz push‐pull compounds and viologens display strong solvatochromism and electrochromism covering a large fluorescence spectral range.[^17^, ^18^, ^19^] In the area of chemosensing, TzTz‐based fluorophores have been used to detect various metal cations, including Hg^2+^,^[20]^ Cu^2+^,[^18^, ^21^] Cr^3+^ and Al^3+^,^[22]^ Cr_2_O_7_^2−^ and MnO_4_^−^ anions,^[23]^ as well as glucose.^[24]^ The mode of detection in these sensing applications varies, ranging from fluorescence quenching through coordination,^[20]^ non‐coordinative electron transfer^[23]^ or radical cation formation,^[18]^ to fluorescence enhancement.^[22]^

**Figure 1:**
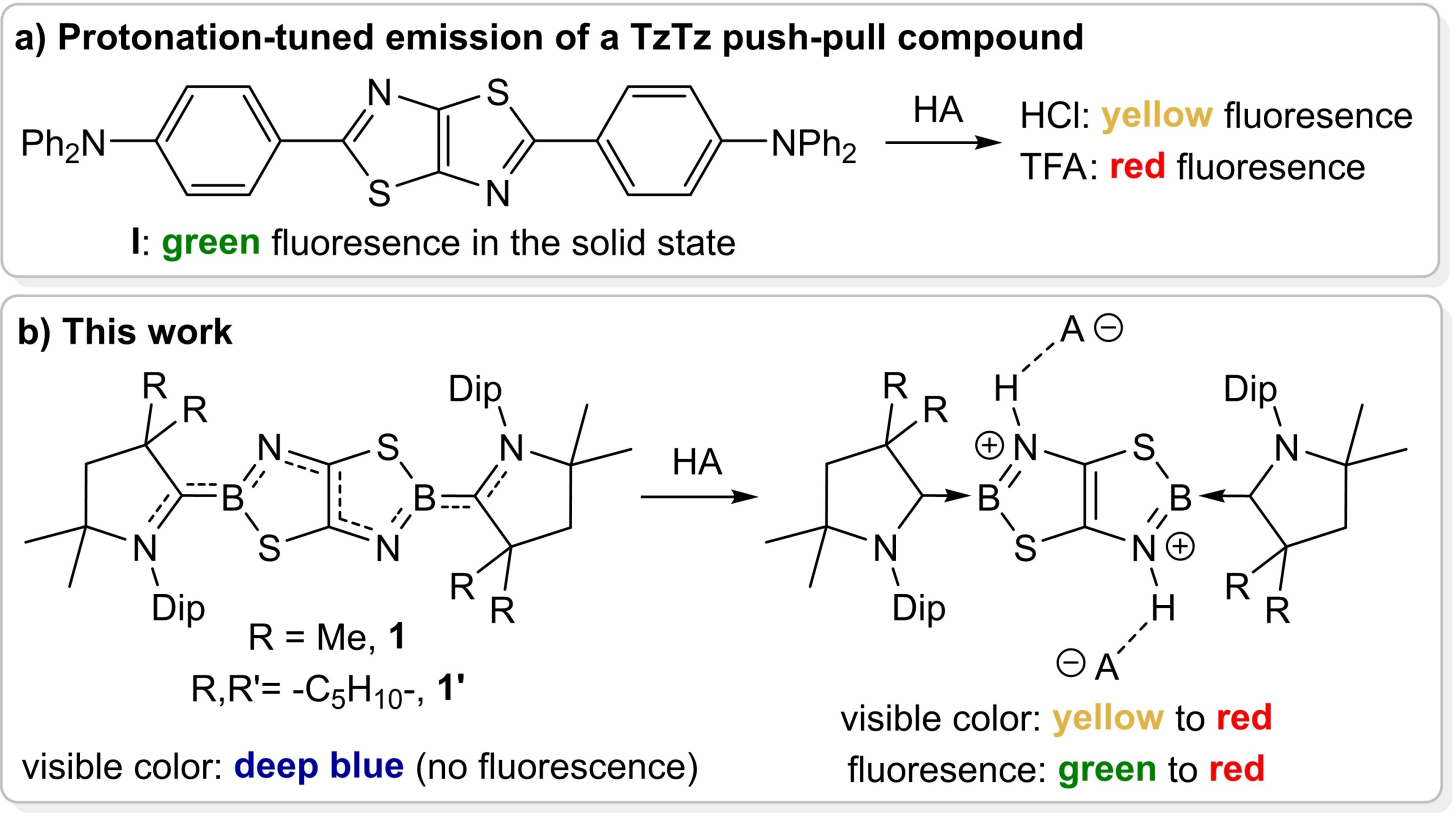
Anion‐dependent tuning of the optical properties of protonated TzTz and TzbTzb heterocycles. TFA=trifluoroacetic acid (HO_2_CCF_3_); Dip=2,6‐*i*Pr_2_C_6_H_3_.

The Lewis‐basic nature of the thiazole nitrogen atom can also be used to great effect, as its protonation significantly shifts both the absorption and emission wavelengths of TzTz‐based compounds.[^25^, ^26^] This has been applied, in particular, to TzTz compounds substituted with 2‐hydroxyaryl groups, which undergo excited‐state intramolecular proton transfers through tautomerization between the thiazolium‐keto and the thiazole‐enol forms.[^27^, ^28^] Furthermore, the TzTz push‐pull compound **I** depicted in Figure 1a has been shown to undergo strong changes in its fluorescence color upon protonation, both in solution and in the solid state, dependent on the nature of the conjugate base anion.^[29]^

Recent work has demonstrated that the incorporation of boron centers into conjugated scaffolds is an efficient strategy for opening up new chemical space and modifying the optoelectronic properties of the resulting compounds and materials.[^30^, ^31^, ^32^] The electrophilic nature of boron makes boron‐containing chromophores particularly apt at sensing anions, with the majority of organoboron‐based sensors being based on triarylboranes.[^33^, ^34^, ^35^, ^36^, ^37^, ^38^, ^39^, ^40^]

We have recently reported the first boron‐doped TzTz analogue, the thiazaborolo[5,4‐*d*]thiazaboroles (TzbTzb) **1** and **1′** (Figure 1b), in which the 2‐ and 2′‐positions of the TzTz core have been replaced by boron atoms.^[41]^ Stabilized by two π‐accepting cyclic alkyl(amino)carbene (CAAC) ligands^[42]^ at the boron centers, these compounds display moderate aromaticity and strong absorptions around 675 nm, giving them a deep blue colour. The replacement of the covalent exocyclic C−C bonds with C_CAAC_→B donor bonds makes these compounds both isosteric and isoelectronic to their TzTz counterparts. We show herein that doubly protonated TzbTzb dications display a pronounced dependence of their colorimetric and fluorescence properties upon the nature of the hydrogen‐bonded counteranions, covering a larger spectral range than their TzTz analogues. DFT‐based computations on these systems reveal that the hydrogen bonding of the counteranions to the protonated nitrogen centers of **1′** affects intramolecular TzbTzb‐to‐CAAC charge transfer (ICT) character involved in the S_0_→S_1_ π‐π* transition, enabling a fine‐tuning of their absorption and emission spectral features.

## Results and Discussion

**Scheme 1:**
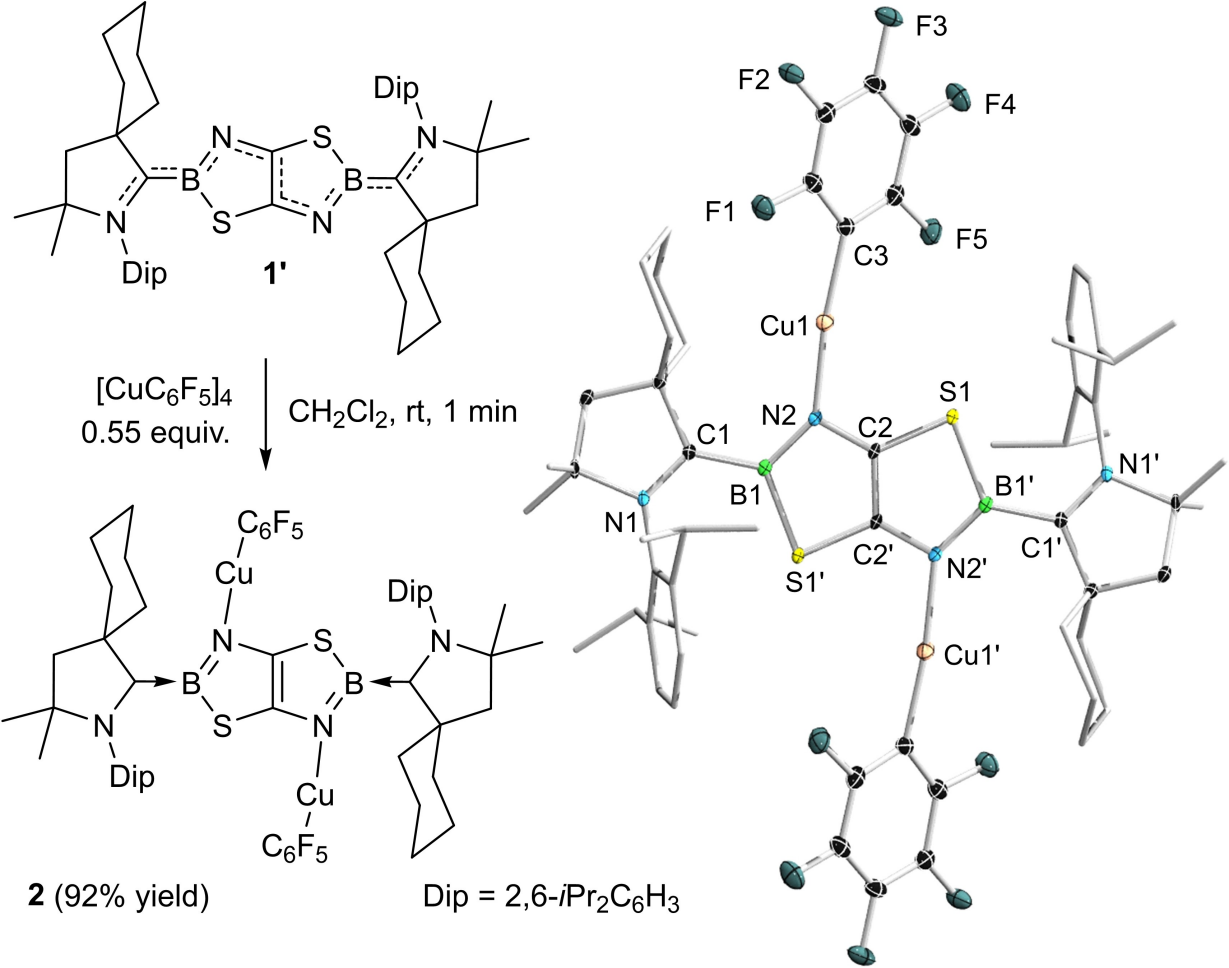
Coordination of Cu(C_6_F_5_) to **1′**. Crystallographically‐derived molecular structure of **2**. Atomic displacement ellipsoids at 50 % probability level. Ellipsoids on the CAAC ligand periphery and hydrogen atoms omitted for clarity. See Table 1 for selected bond lengths and angles.

Treatment of **1′** with 0.55 equivalents of [Cu(C_6_F_5_)]_4_ resulted in an immediate color change from blue to purple and quantitative formation of **2** (Scheme 1). While the ^11^B NMR spectrum remained virtually unchanged (**2**: δ_11B_=33.0 ppm, **1′**: δ_11B_=32.5 ppm), the ^19^F NMR spectrum showed three higher‐order multiplets at −111.16, −162.49 and −164.39, upfield‐shifted compared to [Cu(C_6_F_5_)]_4_ (δ_19F_=−107.2, −153.4, −162.3 ppm),^[43]^ indicating coordination to **1′**. Slow evaporation of a dichloromethane solution afforded deep blue crystals of **2** in 92 % isolated yield.

An X‐ray diffraction analysis revealed that coordination of Cu(C_6_F_5_) takes place at both endocyclic nitrogen atoms N2/N2’ (Scheme 1, Table 1). Compared to those of **1′**, the C1−B1 and N2−C2 bonds (1.577(3) and 1.377(2) Å, respectively) are longer by 2–3 %, whereas the N1−C1 and C2−C2′ bonds (1.316(2) and 1.377(4) Å, respectively) are shorter by 1–3 %, indicating a lower degree of π delocalization in **2**. The UV‐vis absorption spectrum of **2** in CH_2_Cl_2_ shows two maxima of equal intensity at 573 and 621 nm (Table 2, see Figure S47 in the Supporting Information), accounting for the deep purple color, blueshifted compared to **1′** (680 nm).

**Table 1:** Selected bond lengths (Å) and angles (°) for the crystallographically‐derived molecular structures of **1′**, **2** and **3‐X**.

	**1′**	**2**	**3‐Cl**	**3‐OTf**	**3‐OTf** **⋅** **HOTf**	**3‐BAr^F^**_**4**_ **⋅** **Et_2_O**	**3‐BAr^F^**_**4**_
N1−C1	1.325(2)	1.316(2)	1.305(3)	1.301(3)	1.299(2)	1.296(3)	1.300(2)
C1−B1	1.543(2)	1.577(3)	1.581(3)	1.578(3)	1.576(3)	1.579(4)	1.569(2)
B1−N2	1.419(2)	1.422(3)	1.415(3)	1.417(4)	1.410(3)	1.407(4)	1.417(2)
N2−C2	1.332(2)	1.377(2)	1.377(2)	1.386(3)	1.381(3)	1.380(3)	1.376(2)
C2−C2’	1.421(3)	1.377(4)	1.366(4)	1.347(5)	1.353(4)	1.358(5)	1.358(3)
C2’−S1	1.7481(16)	1.7371(19)	1.7324(18)	1.736(3)	1.735(2)	1.726(3)	1.7298(16)
B1−S1	1.8598(18)	1.851(2)	1.838(2)	1.817(3)	1.821(3)	1.809(3)	1.8251(19)
N2−H2^[a]^	–	–	0.88(2)	0.94(4)	0.82(2)	0.92(3)	0.86(2)
H2⋅⋅⋅Y	–	–	2.28(2)^[b]^	2.03(3)^[c]^	2.16(2)^[c]^	2.05(3)^[c]^	2.33(3)^[d]^
N1−C1−B1−N2	170.13(16)	163.33(18)	170.98(17)	147.6(3)	137.7(2)	163.3(3)	166.16(16)

[a] The N‐bound hydrogen atoms were detected in the inverse Fourier map and freely refined. [b] Y=Cl1. [c] Y=O1. [d] Y=F5.

**Table 2:** Photophysical data for **1′**, **2** and **3‐X** in CH_2_Cl_2_ and TD‐DFT results at the ωB97X‐D/def2‐SVP level of theory (vert.=vertical, adiab.=adiabatic).

Compound	λ_max‐abs_ [nm]	λ_max‐calcd_ [nm] [error (eV)]	S_0_→S_1_ Attribution	*f_osc_*	λ_max‐em_ [nm]^[b]^ [calcd.]	Φ_fluor_^[c]^
**1′**	680	579 [0.3] (vert.) 607 [0.2] (adiab.)	HOMO→LUMO (98.4 %)	0.9740	–	–
**2**	573, 621^[a]^	503 [0.3] (vert.)	HOMO→LUMO (96.9 %)	0.6651	–	–
**3‐Cl**	493, 470^[a]^	453 [0.2] (vert.) 490 [0.02] (adiab.)	HOMO‐4→LUMO (74.8 %) HOMO‐2→LUMO (20.0 %) HOMO→LUMO (2.1 %)	0.6810	580 [527]	n.d.^[d]^
**3‐OTf**	474	–	–	–	566	0.23
**3‐OTf** **⋅** **HOTf**	471	390 [0.5] (vert.)	HOMO→LUMO (93.5 %)	0.5227	562	0.27
**3‐BAr^F^**_**4**_ **⋅** **Et_2_O**	450, 473^[a]^	383 [0.5] (vert.) ^[e]^	HOMO‐10→LUMO (95.4 %)^[f]^	0.5812	539	0.33
**3**	–	373 (vert.) 408 (adiab.)	HOMO→LUMO (92.0 %)	0.5155	[446]	–

[a] Second maximum of similar intensity. [b] Excited at the wavelength of the respective absorption maximum. [c] Fluorescence quantum yield determined absolutely with an integrating sphere. [d] The compound was not sufficiently stable in solution to determine its quantum yield. [e] Due to soft‐ and hardware limitations, the system is not fully optimized (see Supporting Information for details). [f] HOMO‐10 is similar to the occupied orbitals shown in Figure 3 (see Figure S57).

**Scheme 2:**
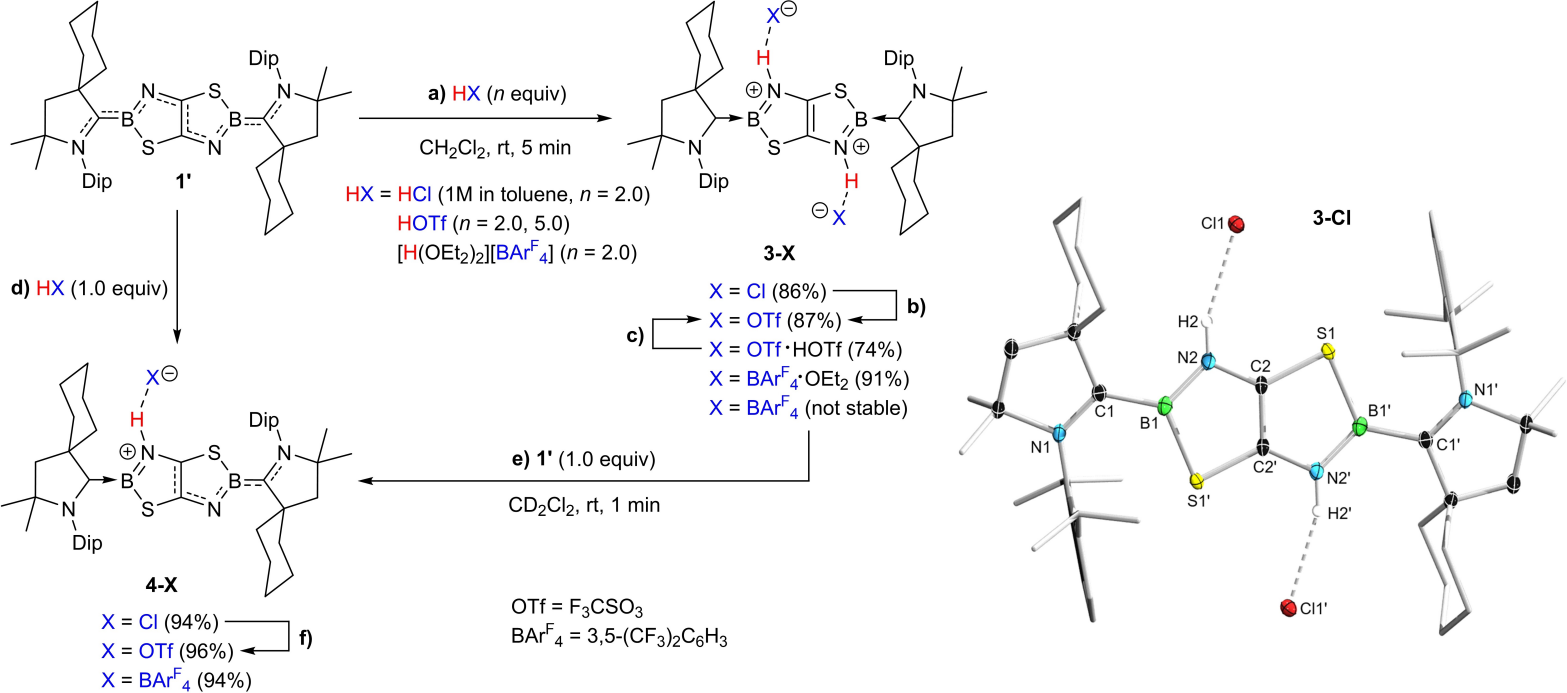
Double and single protonation of **1′** with selected Brønsted acids. Isolated yields in parentheses. **b)** AgOTf (2.0 equiv.), CD_2_Cl_2_, rt, 1 min; **c)** **1′** (1.0 equiv.), CD_2_Cl_2_, rt, 1 min; **f)** AgOTf (1.0 equiv.), CD_2_Cl_2_, rt, 1 min. Crystallographically‐derived molecular structure of the **3‐Cl**. Atomic displacement ellipsoids at 50 % probability level. Ellipsoids on the CAAC ligand periphery and hydrogen atoms, except those bound to nitrogen, are omitted for clarity.

**Figure 2:**
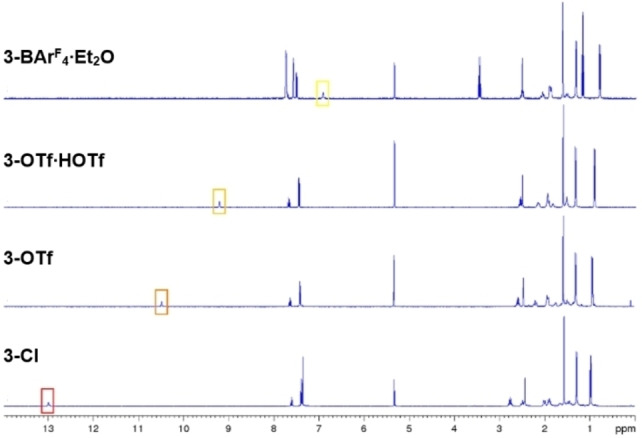
Stack‐plot of ^1^H NMR spectra of **3‐Cl**, **3‐OTf**, **3‐Otf** **⋅** **HOTf** and **3‐BAr^F^**_**4**_ **⋅** **Et_2_O** in CD_2_Cl_2_ showing the gradual upfield shift of the framed N*H* resonance.

Given the nucleophilic nature of the endocyclic nitrogen atoms N2/N2′ in **1′**, we envisaged that their protonation could lead to interesting changes in the optoelectronic properties of the B,N,S‐containing heterocycle. Indeed, treatment of **1′** with 2.0 equivalents of anhydrous HCl (0.1 M in toluene) resulted in an instant color change from blue to red. After workup, **3‐Cl** (δ_11B_=32.8 ppm) was isolated as a red solid in 86 % yield (Scheme 2a). The ^1^H NMR spectrum of **3‐Cl** in CD_2_Cl_2_ showed one symmetrical set of CAAC resonances as well as a broad, highly deshielded 2H singlet at 13.00 ppm, confirming the protonation of N2/N2′ and indicating a very electron‐poor TzbTzb heterocycle (Figure 2). Furthermore, an X‐ray crystallographic analysis of single crystals of **3‐Cl** (see Scheme 2 and Table 1) shows pronounced hydrogen bonding between Cl^−^ and the nitrogen‐bound protons (H2⋅⋅⋅Cl1 2.28(2) Å).

To our surprise, the product of twofold protonation of **1′** with 2.0 equivalents of HOTf, **3‐OTf**, was orange rather than red. Like that of **3‐Cl**, the solid‐state structure of **3‐OTf** (see Figure S52 in the Supporting Information) shows hydrogen bonding between one oxygen atom of OTf^−^ and the nitrogen‐bound protons (H2⋅⋅⋅O1 2.03(3) Å). It is worth mentioning that, while the addition of a large excess of HOTf to **1′** resulted in protonation of the CAAC ligands and decomposition of the TzbTzb heterocycle, the use of 5 equiv. HOTf led to the formation of a HOTf adduct of **3‐OTf**, **3‐OTf** **⋅** **HOTf**, in which the additional HOTf molecules interact with the OTf^−^ anions via O−H⋅⋅⋅O hydrogen bonding (see Figure S53 in the Supporting Information). The equimolar reaction of **1′** and **3‐OTf** **⋅** **HOTf** resulted in the selective formation of **3‐OTf** (Scheme 1c), which can alternatively be obtained by salt metathesis of **3‐Cl** with AgOTf (Scheme 1b). While the ^11^B NMR shift of **3‐OTf** and **3‐OTf** **⋅** **HOTf** (δ_11B_=33.1 and 33.5 ppm, respectively) barely differs from that of **3‐Cl**, the ^1^H NMR resonance of the N*H* protons at 10.50 and 9.20 ppm, respectively, is upfield‐shifted by 2.5 and 3.8 ppm, respectively, compared to **3‐Cl**, indicating a gradual increase in the electron density of the TzbTzb heterocycle (Figure 2).

**Figure 3:**
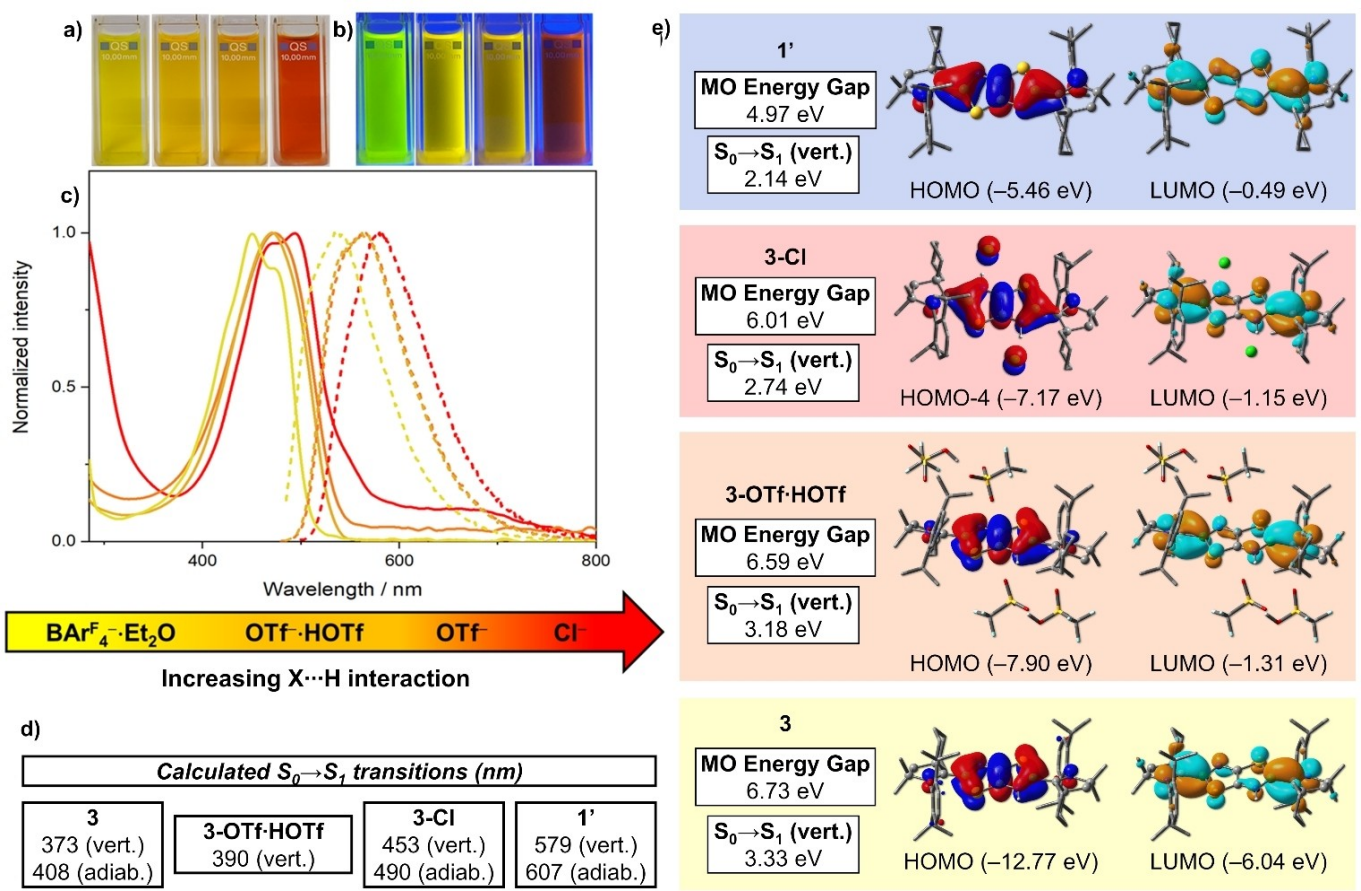
Photographs of solutions of (from left to right) **3‐BAr^F^**_**4**_ **⋅** **Et_2_O**, **3‐OTf** **⋅** **HOTf**, **3‐OTf** and **3‐Cl** under **a)** ambient light (left) and **b)** UV irradiation at 254 and 366 nm simultaneously (right). **c)** UV‐vis absorption (solid lines) and fluorescence (dashed lines) spectra of **3‐X** with X=Cl (red), OTf (dark orange), OTf ⋅ HOTf (light orange), and BAr^F^_4_ ⋅ Et_2_O (yellow) in CH_2_Cl_2_. **d)** Computed S_0_→S_1_ vertical/adiabatic transitions of the naked doubly protonated dication **3** and the neutral compounds **3‐X** (X=Cl, OTf ⋅ HOTf) and **1’**. **e)** Relevant MOs (isosurface: 0.03 a. u.) associated with the S_0_→S_1_ transitions of the aforementioned systems. The corresponding molecular orbital (MO) energy gaps and the S_0_→S_1_ vertical energies in eV are shown in the white boxes. All computations are at the ωB97X‐D/def2‐SVP level. Non‐participating hydrogen atoms are omitted for clarity.

Finally, the twofold protonation of **1′** with Brookhart's acid, [H(OEt_2_)_2_][BAr^F^_4_] (Ar^F^=3,5‐(CF_3_)_2_C_6_H_3_) resulted in the formation of yellow‐colored **3‐BAr^F^**_**4**_ **⋅** **Et_2_O** (δ_11B_=33.7 ppm). The solid‐state structure showed that in this case the N*H* protons are hydrogen‐bonded to the two diethyl ether molecules generated by the H(OEt_2_)_2_^+^ cation, rather than to the BAr^F^_4_^−^ anion (see Figure S54 in the Supporting Information). Again, the ^1^H NMR N*H* resonance is shifted another 2.3 ppm upfield from **3‐OTf** **⋅** **HOTf**, as expected from the weaker N−H⋅⋅⋅O interaction (Figure 2). Removal of Et_2_O *in vacuo* afforded ether‐free, yellow‐colored **3‐BAr^F^**_**4**_, which displays N−H⋅⋅⋅F hydrogen bonding to a CF_3_ group of the BAr^F^_4_^−^ anion in the solid state (see Figure S55 in the Supporting Information). The ^1^H NMR N*H* resonance at 6.84 ppm is similar to that of **3‐BAr^F^**_**4**_ **⋅** **Et_2_O**, suggesting that the N−H⋅⋅⋅OEt_2_ and N−H⋅⋅⋅F_3_C hydrogen bonds are of similar strength. These results show that the electron density and color of the diprotonated TzbTzb heterocycle can be easily tuned by varying the nature of the hydrogen‐bonded anion or donor solvent (see photographs of solutions of **3‐X** in Figure 3a).

The solid‐state IR spectra of **3‐X** showed a broad N−H stretching band in the 3000–3400 cm^−1^, which is shifted to higher frequencies in the order of X=Cl<OTf<OTf ⋅ HOTf<BAr^F^_4_ ⋅ Et_2_O, indicating a gradual strengthening of the N−H bond, concomitant with a weakening of the H⋅⋅⋅X hydrogen bond. This correlates well with the trend observed in the ^1^H NMR shifts of the N*H* protons.

The crystallographically‐derived solid‐state structures of **3‐X** were all centrosymmetric and display hydrogen bonding between the diprotonated core and either the two counteranions or the solvent (see Scheme 2 and Figures S52–S55 in the Supporting Information). The bond lengths in the π‐delocalized (N1−C1−B1−N2−C2)_2_ scaffold change significantly upon protonation of N2/N2′ (see Table 1). The N1−C1 (avg. 1.30 Å) and C1−B1 (avg. 1.58 Å) are indicative of a purely σ‐donating interaction between the CAAC ligands and the TzbTzb unit. While the B1−N2 bond length (avg. 1.41 Å) remains essentially unchanged, the N2−C2 bond (avg. 1.38 Å) is slightly lengthened and the B1−S1 (avg. 1.82 Å) and C2−C2′ bonds (avg. 1.34 Å) are shortened, the latter now being a double bond. This results in a disruption of the π delocalization, similar to that observed in the copper complex **2**. Similar changes in the endocyclic bond lengths were observed upon protonation of the related TzTz compound **I** (Figure 1a) with trifluoroacetic acid.^[29]^ Whereas the CAAC ligands in **1′** are oriented so as to maximize the π overlap between the N1−C1 bonds and the TzbTzb heterocycle, as shown by the N1−C1−B1−N2 torsion angle (170.13(16)°) tending towards 180°, the degree of rotation of the CAAC ligands away from coplanarity in **3‐X** increases, as seen in the N1−C1−B1−N2 torsion angle decreasing from 170.98(17)° to 137.7(2)°, in the order X = Cl>BAr^F^_4_>BAr^F^_4_ ⋅ Et_2_O>OTf>OTf ⋅ HOTf. The pronounced rotation of the CAAC ligands in the triflate derivatives is in agreement with the absence of B1‐to‐C1 π backbonding. The lack of steric or electronic trend in the variation of the N1−C1−B1−N2 torsion angle, however, suggests that this may simply be a crystal packing effect in the solid state rather than an electronic effect.

The UV‐vis spectra of **3‐X** recorded in CH_2_Cl_2_ each display one structured absorption band with a maximum in the λ_max_=450–493 nm range, gradually blueshifted in the order X = Cl>OTf≈OTf ⋅ HOTf>BAr^F^_4_ ⋅ Et_2_O (Figure 3c, Table 2). The absorption spectra of **3‐Cl** and **3‐BAr^F^**_**4**_ **⋅** **Et_2_O** display a second maximum, λ_2_, of slightly lower intensity, blueshifted by 20 nm for **3‐Cl** and redshifted by 20 nm for **3‐BAr^F^**_**4**_ **⋅** **Et_2_O**. The ether‐free species **3‐BAr^F^**_**4**_ was not sufficiently stable in solution to acquire photospectroscopic data. Remarkably, under UV irradiation, solutions of **3‐X** in CH_2_Cl_2_ were brightly fluorescent, with emission colors ranging from red for **3‐Cl** to green for **3‐BAr^F^**_**4**_ **⋅** **Et_2_O**, via yellow for **3‐OTf** and **3‐OTf** **⋅** **HOTf** (see photographs of irradiated solutions of **3‐X** in Figure 3a). The fluorescence spectra of **3‐X** showed an unstructured emission band, gradually blueshifted in the same sequence as the absorption spectra, from 580 for **3‐Cl** to 539 nm for **3‐BAr^F^**_**4**_ **⋅** **Et_2_O** (Table 2). This hypsochromic shift reflects the gradual decrease in the electron‐accepting strength of the diprotonated TzbTzb core, in line with the upfield shift of the ^1^H NMR resonances of the N*H* protons (Figure 2). A similar conjugate base‐dependence has been observed with the doubly protonated form of the TzTz compound **I** (Figure 1a), the absorption and emission maxima of which are bathochromically shifted by 27 nm upon switching from HCl (λ_max_=492 (absorption), 600 (emission) nm) to trifluoroacetic acid (λ_max_=517 (absorption), 627 (emission) nm) as the proton source.^[29]^ The isosteric and isoelectronic replacement of the exocyclic C−C bonds of **I** with the C_CAAC_→B donor bonds of **1** thus alters both the direction of the emission shift and its spectral range. In the TzTz case, the authors speculated that the emission shift reflects an increase in the electron‐accepting nature of the TzTz core as π overlap with the triarylamino groups decreases due to steric constraints imposed by the hydrogen‐bonded counteranion.^[29]^ In our case, since the comparison of the solid‐state structures of **3‐X** excludes steric effects, the modulation of the naked‐eye and fluorescence colors must be entirely dependent on the charge stabilization effect of the hydrogen‐bonded counteranions or diethyl ether.

In order to shed some light on the intriguing spectroelectronic behavior of **3‐X** we conducted TD‐DFT computations on selected systems amongst those described herein. We adopted the ωB97X‐D^[44]^ functional in combination with the def2‐SVP^[45]^ basis set, as charge transfer (CT) states may play a role and are considerably underestimated by DFT functionals that do not include range‐separated corrections, such as B3LYP.^[46]^ The computed vertical energies, Δ*E_vert_*, overestimate the measured absorption energies by 0.3 to 0.5 eV (Table 2), and motivated us to investigate photoinduced relaxation effects on the computationally accessible systems **1′**, **3**, and **3‐Cl**. Indeed, the computed adiabatic excitation energies, Δ*E_adiab_*, are 0.1 eV (**1′**) to 0.3 eV (**3**) lower in energy than the corresponding vertical ones, and fit much better with the experimental results. The systems **3‐BAr^F^**_**4**_ **⋅** **Et_2_O** and **3‐OTf** **⋅** **HOTf** are too large for geometry optimizations in the S_1_ state. However, considering that similar effects as those observed for **3** are also at play, the computed energies would differ by only about 0.2 eV from the experimental counterparts. Based on the better agreement of the adiabatic energies, and also taking into account the small geometric variations between the equilibrium geometries of S_0_ and S_1_ (see Table S1 in the Supporting Information), we attribute the maximum of the measured bands to 0–0 transitions between the S_0_ and the S_1_ states.

Inspection of the MOs (isosurface: 0.03 a. u.) involved in the S_0_→S_1_ transition (see Figure 3e) shows that in all cases a π‐π* excitation is observed. Interestingly, the intramolecular TzbTzb‐to‐carbene charge transfer (ICT) character depends on the charge state and, in the case of **3‐X**, on the strength of the X⋅⋅⋅H interaction. The HOMO of **1′** (Figure 3e) is extended from the TzbTzb core to the carbene C1 and C1′ atoms (see Scheme 2 for atom labels). This orbital has π(C1,B1,N2); π(C2,C2’) character, while nodal planes crossing the sulfur atoms are observed. In turn, the LUMO presents additional nodal planes between the B1−N2 and C2−C2’ nuclei, and features a smaller participation of the TzbTzb ring. The computed MO energy gap of **1′** and the S_0_→S_1_ vertical excitation energy are 4.97 eV and 2.14 eV, respectively, the smallest values among the systems studied herein. At the other end, the naked, diprotonated TzbTzb molecule **3** has the largest computed values for both properties (6.73 eV and 3.33 eV, respectively). Inspection of the corresponding MOs reveals that, while the LUMO of **3** is fairly similar to that of **1′**, the HOMO of **3** is mainly localized on the TzbTzb ring. As a consequence, the S_0_→S_1_ excitation of **3** has a larger ICT character than that of **1′**. This is corroborated by additional computations based on the interfragment charge transfer (IFCT)[^47^, ^48^, ^49^] method and the Λ diagnostic.^[50]^ The IFCT method estimates a larger net transfer of electrons from the TzbTzb core to the CAAC ligands in **3** (0.49) than in **1′** (0.39) (see Figure S59 for the corresponding charge density difference plots). Accordingly, the Λ diagnostic analysis indicates a larger hole‐electron overlap degree for the S_0_→S_1_ excitation of **1′** (0.78) than for **3** (0.57).

The larger HOMO‐LUMO gap of **3** relative to that of **1′** suggests a blueshifted S_0_→S_1_ transition for the conjugate acids of **1′** (**3‐X**) with respect to the neutral system. This is in agreement with the experimental findings. The computed S_0_→S_1_ ICT character, excitation energies, and orbital energy gaps of **3‐X** are in between those of **1′** and **3** and dependent on the effectiveness of the positive net charge stabilization of the diprotonated TzbTzb ring by X. As a consequence, less blueshifted transitions are found for systems featuring strongly coordinating counteranions, where the strong X⋅⋅⋅H interactions at play lead to a better charge equilibration that ultimately lowers the electron excitation energy. The computed blueshift for more weakly coordinating counteranions in the absorption and emission bands nicely reflects the experimental trends (see Figure 3 and Table 2), and can be fully explained by the present calculations. Finally, the secondary maxima observed for species such as **3‐Cl** and **3‐BAr^F^**_**4**_ **⋅** **Et_2_O** (Table 2) could probably be assigned to higher vibrational states of the S_1_ electronic state.

**Figure 4:**
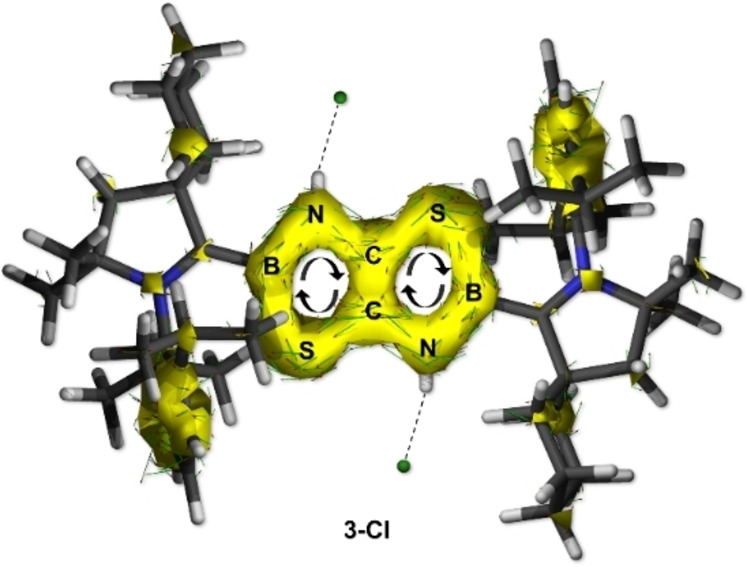
ACID plot of **3‐Cl** at the ωB97X‐D/def2‐SVP level of theory. Contour value: 0.02.

In light of the larger HOMO‐LUMO gap of **3** with respect to **1′** and the lack of B1‐to‐C1 π backbonding for both the diprotonated and metal‐coordinated species, we became interested in assessing the aromaticity of the “boron‐doped” thiazolothiazole derivatives described herein. Table 3 shows the computed nucleus‐independent chemical shift (NICS) values,[^51^, ^52^, ^53^, ^54^] namely NICS(0), NICS(1), and NICS_zz_(1), of **1′**, **2**, **3‐Cl**, and **3**, while Figure 4 shows the anisotropy of the induced current density (ACID)^[55]^ plot of **3‐Cl**. All the computed NICS values are negative, suggesting that the TzbTzb rings are, indeed, aromatic. Likewise, a diatropic clockwise ring current circulation around the TzbTzb core, typical of aromatic systems, is observed in **3‐Cl** (Figure 4). We conclude that, similarly to **1′**, all other TzbTzb compounds investigated in this work also feature aromaticity.

**Table 3:** Computed NICS(0), NICS(1), and NICS_zz_(1) values of **1′**, **2**, **3‐Cl** and **3** at the ωB97X‐D/def2‐SVP level of theory.

Compound	NICS(0)	NICS(1)	NICS_zz_(1)
**1′**	−7.7	−8.0	−14.6
**2**	−8.8	−7.8	−13.1
**3‐Cl**	−10.0	−8.3	−14.9
**3**	−10.4	−7.7	−14.7

Given the strong influence of the twofold protonation of **1′** on the color of the resulting compounds **3‐X**, we wondered whether the selective single protonation of **1′** would yield compounds with intermediate optoelectronic properties. While equimolar reactions between **1′** and HCl, HOTf or [H(OEt_2_)_2_][BAr^F^_4_] did yield the desired monoprotonated compounds **4‐X** (Scheme 2d), the difficulty of getting the stoichiometry exactly right at such small reaction scales (typically 10–20 mg of **1′**) to prevent the formation of small amounts of diprotonated **3‐X** marred the clean isolation of **4‐X** following this synthetic route. In contrast, the comproportionation of **1′** and **3‐X** in CD_2_Cl_2_ provided intense blue solutions of analytically pure **4‐X** (Scheme 2e). Alternatively, **4‐OTf** could be synthesized by salt metathesis of **4‐Cl** with one equiv. AgOTf (Scheme 2f). Whereas **4‐Cl** shows a single broad ^11^B NMR resonance at 32.0 ppm, **4‐OTf** and **4‐BAr^F^**_**4**_ **⋅** **Et_2_O** show two overlapping ^11^B NMR resonances at 32.5 and 31.5 ppm, and 32.2 and 31.2 ppm, respectively, for the two slightly different boron environments of the protonated and neutral Tzb moieties, respectively. While the ^1^H and ^13^C{^1^H} NMR spectra of **4‐X** show two sets of magnetically inequivalent and partly broadened CAAC resonances, suggesting fluxional behavior in solution, this could not be probed further due to the instability of these species in solution. For **4‐Cl** and **4‐OTf** the ^1^H NMR resonance of the N*H* proton (integrating for 1H) is shifted ca. 3.5 ppm upfield from the corresponding diprotonated species, **3‐Cl** and **3‐OTf**, to 9.42 and 7.13 ppm, respectively (see Figures S35–S37 in Supporting Information), as expected for the more electron‐rich monocationic heterocycle. In contrast, the N*H* resonances of **3‐BAr^F^**_**4**_ **⋅** **Et_2_O** and **4‐BAr^F^**_**4**_ **⋅** **Et_2_O** both appear at ca. 6.9 ppm, which suggests that N−H⋅⋅⋅OEt_2_ hydrogen bonding has little influence on the electron density of the heterocycle in solution, effectively leaving the mono‐ or dication “naked”. While **4‐X** and **3‐X** proved stable in the solid state under inert atmosphere over several months, the low stability of **4‐X** in solution over longer periods of time, even at −30 °C, prevented the isolation of single crystals suitable for X‐ray diffraction experiments. Unlike their diprotonated counterparts **3‐X**, compounds **4‐X** were not fluorescent and their UV‐vis spectra showed complex patterns of bands suggesting fluxional behavior.

## Conclusion

The protonation of the two Lewis‐/Brønsted‐basic nitrogen atoms of the doubly CAAC‐stabilized TzbTzb heterocycle of **1′** to the salts **3‐X** induces a stark color change from deep blue to yellow, orange or red, the absorption redshift increasing with the electron‐accepting strength of the fused heterocycle and the electron‐donating ability of the hydrogen‐bonded counteranions or solvent molecules X (BAr^F^_4_ ⋅ Et_2_O<OTf ⋅ HOTf≈OTf<Cl). Furthermore, the doubly protonated TzbTzb salts are fluorescent in solution, their emission color varying from green to yellow to red as the electron‐donating strength of X increases, the emission shift and spectral range differing significantly from their diprotonated TzTz counterparts. In contrast, the monoprotonated TzbTzb heterocycles are unstable and display no fluorescence behavior. DFT calculations show that the coordination of X subtly influences the relative energy levels of the aromatic TzbTzb core in **3‐X**. Modulation of the X⋅⋅⋅H interaction enables fine‐tuning of the HOMO‐LUMO gap and the S_0_→S_1_ transition, which influence the absorption and emission features of **3‐X** and make these compounds potentially suitable for applications in anion sensing. The study also highlights the potential of the isosterism between exocyclic covalent C−C bonds and C_carbene_→B bonds for altering the optoelectronic properties of conjugated molecules.

## Experimental Section

Crystallographic data: Deposition Numbers 2128816 (**3‐OTf**), 2128817 (**3‐BAr** ^**F**^_**4**_ **⋅** **Et**_**2**_ **O**), 2128818 (**3‐OTf** **⋅** **HOTf**), 2128819 (**2**), 2128820 (**3‐Cl**), 2128821 (**3‐BAr** ^**F**^_**4**_) contain the supplementary crystallographic data for this paper. These data are provided free of charge by the joint Cambridge Crystallographic Data Centre and Fachinformationszentrum Karlsruhe Access Structures service.

## Conflict of interest

The authors declare no conflict of interest.

1

## Supporting information

As a service to our authors and readers, this journal provides supporting information supplied by the authors. Such materials are peer reviewed and may be re‐organized for online delivery, but are not copy‐edited or typeset. Technical support issues arising from supporting information (other than missing files) should be addressed to the authors.

Supporting Information

## Data Availability

The data that support the findings of this study are available from the corresponding author upon reasonable request.
