# Supplementary material for: Modulation of the Naked‐Eye and Fluorescence Color of a Protonated Boron‐Doped Thiazolothiazole by Anion‐Dependent Hydrogen Bonding
Source: Chemistry. 2022 Jul 13;28(49):e202201398. doi: 10.1002/chem.202201398 (PMC9541717; doi:10.1002/chem.202201398)
Supplement: Supplementary file 1 — Supporting Information [file CHEM-28-0-s001.pdf]

Chemistry–A European Journal

Supporting Information

Modulation of the Naked-Eye and Fluorescence Color of a Protonated Boron-Doped Thiazolothiazole by Anion-Dependent Hydrogen Bonding

Stephan Hagspiel, Felipe Fantuzzi, Merle Arrowsmith, Annalena Gärtner, Maximilian Fest, Jonas Weiser, Bernd Engels, Holger Helten, and Holger Braunschweig*

Contents

Methods and materials S2

Synthetic procedures S3

NMR spectra of isolated compounds S9

IR spectra S47

UV-vis spectra S55

X-ray crystallographic data S57

Computational details S65

Cartesian coordinates (Å) S68

References S110

Methods and materials

All manipulations were performed either under an atmosphere of dry argon or *in vacuo* using standard Schlenk line or glovebox techniques. Deuterated solvents were dried over molecular sieves and degassed by three freeze-pump-thaw cycles prior to use. All other solvents were distilled and degassed from appropriate drying agents. Both deuterated and non-deuterated solvents were stored under argon over activated 4 Å molecular sieves. NMR spectra were acquired either on a Bruker Avance 500 or a Bruker Avance 400 NMR spectrometer. Chemical shifts (δ) are reported in ppm and internally referenced to the carbon nuclei ($^{13}C\{^1H\}$) or residual proton signals (^1H) of the solvent. Heteronuclei NMR spectra are referenced to external standards (^{11}B: $BF_3{\cdot}OEt_2$; ^{19}F: Cl_3CF). NMR multiplicities are given as: s (singlet), d (doublet), t (triplet), q (quartet), sept (septet), m (multiplet), br (broad). Solid-state IR spectra were recorded on a Bruker FT-IR spectrometer ALPHA II inside a glovebox. UV-vis spectra were measured on a METTLER TOLEDO UV-vis-Excellence UV5 spectrophotometer. Emission spectra were recorded using an Edinburgh Instruments FLSP920 spectrometer equipped with a double monochromator for both excitation and emission, operating in right-angle geometry mode, and all spectra were fully corrected for the spectral response of the instrument. Fluorescence quantum yields were measured using a calibrated integrating sphere from Edinburgh Instruments combined with the FLSP920 spectrometer described above. Microanalyses (C, H, N, S) were performed on an Elementar vario MICRO cube elemental analyzer. High-resolution mass spectrometry (HRMS) data were obtained from a Thermo Scientific Exactive Plus spectrometer. *Note: both elemental analyses and HRMS were carried out for all new compounds but in some cases these decomposed too rapidly and only one type of analysis was possible.*

Solvents and reagents were purchased from Sigma-Aldrich or Alfa Aesar. Compound **1'**[1] and $[H(OEt_2)_2][BAr^F_4]$ (Brookhart's acid, BAr^F_4 = tetrakis[3,5-bis(trifluoromethyl)phenyl]borate)[2] were synthesized using literature procedures.

Synthetic procedures

[(CAAC)BN(CuC$_6$F$_5$)CS]$_2$, 2:

1' (50.0 mg, 63.4 µmol) and [CuC$_6$F$_5$]$_4$ (32.1 mg, 34.9 µmol, 0.550 eq.) were combined in 1 mL of dichloromethane, whereupon an intense purple solution was obtained. Slow evaporation of the solvent yielded a large crop of dark blue crystals of **2** (72.9 mg, 58.3 µmol, 92% yield) suitable for X-ray diffraction analysis. ^{1}H NMR (500 MHz, CD$_2$Cl$_2$, 297 K): δ = 7.46 (t, ^3J = 7.7 Hz, 2H, *p*-Ar*H*), 7.30 (d, ^3J = 7.8 Hz, 4H, *m*-Ar*H*), 3.16–3.11 (m, 4H, Cy-C*H*$_2$), 2.71 (sept, ^3J = 6.7 Hz, 4H, *i*Pr-C*H*), 2.35 (s, 4H, C*H*$_2$), 1.97 (d, ^2J = 12.9 Hz, 4H, Cy-C*H*$_2$), 1.83 (d, ^2J = 12.9 Hz, 4H, Cy-C*H*$_2$), 1.78–1.61 (m, 2H, Cy-C*H*$_2$), 1.59–1.43 (m, 4H, Cy-C*H*$_2$), 1.48 (s, 12H, NC(C*H*$_3$)$_2$), 1.41–1.29 (m, 2H, Cy-C*H*$_2$), 1.24 (d, ^3J = 6.6 Hz, 12H, *i*Pr-C*H*$_3$), 1.00 (d, ^3J = 6.7 Hz, 12H, *i*Pr-C*H*$_3$) ppm. ^{13}C{^{1}H} NMR (126 MHz, CD$_2$Cl$_2$, 297 K): δ = 204.2 (C$_{carbene}$, identified by HMBC), 149.9 (*C*F), 145.4 (*o*-Ar*C*), 137.3 (*C*F), 132.3 (*i*-Ar*C*), 131.4 (*p*-Ar*C*), 127.1 (*m*-Ar*C*), 125.8 (*C*F), 80.4 (N*C*(CH$_3$)$_2$), 58.5 (*C*(C$_5$H$_{10}$)), 46.3 (*C*H$_2$), 38.5 (Cy-*C*H$_2$), 29.9 (NC(*C*H$_3$)$_2$), 29.7 (*i*Pr-*C*H), 26.4 (*i*Pr-*C*H$_3$), 25.0 (Cy-*C*H$_2$), 24.7 (*i*Pr-*C*H$_3$), 22.5 (Cy-*C*H$_2$) ppm. *Note: the C$_{NCS}$ and C$_{CuC}$ resonances were not detected.* ^{11}B NMR (128 MHz, CD$_2$Cl$_2$, 297 K): δ = 33.0 (br) ppm. ^{19}F NMR (471 MHz, CD$_2$Cl$_2$, 297 K): δ = –111.16 (dm, $^3J_{FF}$ = 22.8 Hz, 2F, *o*-C*F*), –162.49 (t, $^3J_{FF}$ = 19.6 Hz, 1F, *p*-C*F*), –164.39 (ddm, $^3J_{FF}$ = 22.8, 19.6 Hz, 2F, *m*-C*F*) ppm. Elemental analysis for [C$_{60}$H$_{70}$B$_2$Cu$_2$F$_{10}$N$_4$S$_2$] (M$_w$ = 1250.06): calcd. C 57.65, H 5.64, N 4.48, S 5.13%; found C 57.35, H 5.64, N 4.54, S 4.86%.

[{(CAAC)BNHCS}$_2$][Cl]$_2$, 3-Cl:

To a solution of **1'** (100 mg, 0.13 µmol) in 3 mL of dichloromethane HCl (2.60 mL, 0.10 M in toluene, 0.26 µmol, 2.00 eq.) was added dropwise under vigorous stirring at room temperature. The resulting red solution was treated with 15 mL of pentane to induce precipitation. The suspension was filtered and the resulting solid was washed with pentane. Drying under atmospheric pressure yielded **3-Cl** (93.9 mg, 0.11 mmol, 86% yield) as a red solid. Single crystals suitable for X-ray diffraction analysis were obtained by vapor diffusion of hexane into a saturated dichloromethane solution. ^{1}H NMR (500 MHz, CD$_2$Cl$_2$, 297 K): δ = 13.00 (br, 2H, N*H*), 7.60 (t, ^3J = 7.8 Hz, 2H, *p*-Ar*H*), 7.38 (d, ^3J = 7.9 Hz, 4H, *m*-Ar*H*), 2.76 (sept, ^3J = 6.6 Hz, 4H, *i*Pr-C*H*), 2.51–2.45 (m, 4H, Cy-C*H*$_2$), 2.43 (s, 4H, C*H*$_2$), 2.00 (br d, ^3J = 12.8 Hz, 4H, Cy-C*H*$_2$), 1.94–1.85 (m, 2H, Cy-C*H*$_2$), 1.90 (br d, ^3J = 12.6 Hz, 4H, Cy-C*H*$_2$), 1.69–1.64 (m, 2H, Cy-C*H*$_2$), 1.57 (s, 12H, NC(C*H*$_3$)$_2$), 1.50–1.41 (m, 4H, Cy-C*H*$_2$), 1.29 (d, ^3J = 6.6 Hz, 12H,

*i*Pr-C*H*$_3$), 0.98 (d, ^3J = 6.6 Hz, 12H, *i*Pr-C*H*$_3$) ppm. ^{13}C{^{1}H} NMR (126 MHz, CD_2Cl_2, 297 K): δ = 204.3 ($C_{carbene}$, identified by HMBC), 145.1 (*o*-Ar*C*), 132.7 (*i*-Ar*C*), 132.2 (*p*-Ar*C*), 131.3 (*p*-Ar*C*), 127.3 (*m*-Ar*C*), 83.4 (N*C*(CH$_3$)$_2$), 59.4 (*C*(C$_5$H$_{10}$)), 45.4 (*C*H$_2$), 36.0 (Cy-*C*H$_2$), 29.8 (NC(*C*H$_3$)$_2$), 29.6 (*i*Pr-*C*H), 26.3 (*i*Pr-*C*H$_3$), 24.8 (*i*Pr-*C*H$_3$), 23.9 (Cy-*C*H$_2$), 22.3 (Cy-*C*H$_2$) ppm. *Note: the C_{NCS} resonance was not detected.* ^{11}B NMR (128 MHz, CD_2Cl_2, 297 K): δ = 32.8 (br) ppm. Solid-state IR: ν(NH) = 3061 cm^{-1}. Elemental analysis for [$C_{48}H_{72}B_2Cl_2N_4S_2$] ($M_w$ = 861.77): calcd. C 66.90, H 8.42, N 6.50, S 7.44%; found C 65.24, H 8.44, N 6.17, S 8.06%. HRMS LIFDI for [$C_{48}H_{71}B_2N_4S_2$] = [M – 2Cl – H]$^+$: calcd. 789.5301; found 789.5279.

[{(CAAC)BNHCS}$_2$][OTf]$_2$, 3-OTf:

Route A. HOTf (19.0 mg, 126.8 μmol, 2.00 eq.) diluted in 0.5 mL of dichloromethane was added dropwise to a stirred solution of **1'** (50.0 mg, 63.4 μmol) in 2 mL of dichloromethane, whereupon the color changed from intense blue to bright orange. After the addition was completed the solvent was allowed to evaporate under atmospheric pressure, yielding orange crystals of **3-OTf** (60.0 mg, 55.2 μmol, 87% yield) suitable for X-ray diffraction analysis.

Route B. **3-Cl** (15.0 mg, 17.4 μmol) and AgOTf (8.95 mg, 34.8 μmol, 2.00 eq.) were combined in 0.5 mL of CD_2Cl_2, whereupon the color changed from red to orange, accompanied by formation of a colorless precipitate. After 5 min the suspension was filtered, yielding an intense orange solution of analytically pure **3-OTf** as determined by NMR-spectroscopic analysis.

Route C. **3-OTf·HOTf** (15.0 mg, 10.8 μmol) and **1'** (17.0 mg, 21.6 μmol, 2.00 eq.) were combined in 0.5 mL of CD_2Cl_2. The NMR spectra of the resulting orange solution of **3-OTf** were identical with those from Routes A and B. ^{1}H NMR (500 MHz, CD_2Cl_2, 297 K): δ = 10.50 (br, 2H, N*H*), 7.62 (t, ^3J = 7.8 Hz, 2H, *p*-Ar*H*), 7.40 (d, ^3J = 7.9 Hz, 4H, *m*-Ar*H*), 2.57 (sept, ^3J = 6.6 Hz, 4H, *i*Pr-C*H*), 2.45 (s, 4H, C*H*$_2$), 2.19 (dt, ^2J = 13.4 Hz, ^3J = 3.4 Hz, 4H, Cy-C*H*$_2$), 1.96–1.88 (m, 8H, Cy-C*H*$_2$), 1.79–1.72 (m, 2H, Cy-C*H*$_2$), 1.67–1.36 (m, 6H, Cy-C*H*$_2$), 1.57 (s, 12H, NC(C*H*$_3$)$_2$), 1.30 (d, ^3J = 6.6 Hz, 12H, *i*Pr-C*H*$_3$), 0.93 (d, ^3J = 6.6 Hz, 12H, *i*Pr-C*H*$_3$) ppm. ^{13}C{^{1}H} NMR (126 MHz, CD_2Cl_2, 297 K): δ = 202.2 ($C_{carbene}$, identified by HMBC), 144.8 (*o*-Ar*C*), 132.7 (*i*-Ar*C*), 132.1 (*p*-Ar*C*), 131.0 (*p*-Ar*C*), 127.5 (*m*-Ar*C*), 84.0 (N*C*(CH$_3$)$_2$), 59.3 (*C*(C$_5$H$_{10}$)), 45.4 (*C*H$_2$), 35.9 (Cy-*C*H$_2$), 29.8 (NC(*C*H$_3$)$_2$), 29.7 (*i*Pr-*C*H), 25.9 (*i*Pr-*C*H$_3$), 24.8 (*i*Pr-*C*H$_3$), 24.1 (Cy-*C*H$_2$), 21.9 (Cy-*C*H$_2$) ppm. *Note: the C_{NCS} and C_{CF3} resonances were not detected.* ^{11}B NMR (128 MHz, CD_2Cl_2, 297 K): δ = 33.1 (br) ppm. ^{19}F NMR (471 MHz, CD_2Cl_2, 297 K): δ = –78.5 (s) ppm. Solid-state IR: ν(NH) = 3170 cm^{-1}. Elemental analysis for

[$C_{50}H_{72}B_2F_6N_4O_6S_4$] ($M_w$ = 1089.00): calcd. C 55.15, H 6.66, N 5.14, S 11.78%; found C 54.48, H 6.73, N 5.08, S 11.73%.

[{(CAAC)BNHCS}$_2$][OTf(HOTf)]$_2$, 3-OTf·HOTf:

To a solution of **1'** (50.0 mg, 63.4 µmol) in 2 mL of dichloromethane an excess of HOTf (ca. 5.00 eq.) was added dropwise. The solvent of the resulting yellow solution was allowed to evaporate under atmospheric pressure, yielding yellow single crystals of **3-OTf·HOTf** (65.2 mg, 46.9 µmol, 74% yield). ^{1}H NMR (500 MHz, CD_2Cl_2, 297 K): δ = 15.60 (very br, 2H, *H*OTf), 9.20 (br, 2H, N*H*), 7.66 (t, ^3J = 7.8 Hz, 2H, *p*-Ar*H*), 7.43 (d, ^3J = 7.9 Hz, 4H, *m*-Ar*H*), 2.53 (sept, ^3J = 6.7 Hz, 4H, *i*Pr-C*H*), 2.48 (s, 4H, C*H*$_2$), 2.17–2.11 (m, 4H, Cy-C*H*$_2$), 1.95–1.87 (m, 8H, Cy-C*H*$_2$), 1.84–1.78 (m, 2H, Cy-C*H*$_2$), 1.58 (s, 12H, NC(C*H*$_3$)$_2$), 1.55–1.47 (m, 6H, Cy-C*H*$_2$), 1.30 (d, ^3J = 6.6 Hz, 12H, *i*Pr-C*H*$_3$), 0.89 (d, ^3J = 6.6 Hz, 12H, *i*Pr-C*H*$_3$) ppm. ^{13}C{^{1}H} NMR (126 MHz, CD_2Cl_2, 297 K): δ = 200.5 ($C_{carbene}$, identified by HMBC), 144.6 (*o*-Ar*C*), 133.0 (*i*-Ar*C*), 131.6 (*p*-Ar*C*), 130.9 (*p*-Ar*C*), 127.8 (*m*-Ar*C*), 119.7 (q, $^1J_{13C,19F}$ = 318 Hz, *C*F$_3$), 83.3 (N*C*(CH$_3$)$_2$), 59.3 (*C*(C$_5$H$_{10}$)), 45.4 (*C*H$_2$), 36.3 (Cy-*C*H$_2$), 29.8 (NC(*C*H$_3$)$_2$), 29.8 (*i*Pr-*C*H), 25.7 (*i*Pr-*C*H$_3$), 24.7 (*i*Pr-*C*H$_3$), 24.3 (Cy-*C*H$_2$), 21.8 (Cy-*C*H$_2$) ppm. *Note: the C_{NCS} resonance was not detected.* ^{11}B NMR (128 MHz, CD_2Cl_2, 297 K): δ = 33.5 (br) ppm. ^{19}F NMR (471 MHz, CD_2Cl_2, 297 K): δ = –78.3 (s) ppm. Solid-state IR: ν(NH+OH) = 3288 (br) cm^{-1}. Elemental analysis for [$C_{52}H_{74}B_2F_{12}N_4O_{12}S_6$] ($M_w$ = 1389.14): calcd. C 44.96, H 5.37, N 4.03, S 13.85%; found C 45.28, H 5.14, N 4.22, S 14.31%.

[{(CAAC)BNHCS}$_2$][BAr$^F_4$]$_2$, 3-BAr$^F_4$:

[H(OEt$_2$)$_2$][BAr$^F_4$] (Brookhart's acid) (128 mg, 127 µmol, 2.00 eq.) dissolved in 1 mL of Et$_2$O was added dropwise to a stirred suspension of **1'** (50.0 mg, 63.4 µmol) in 1 mL of Et$_2$O, whereupon the reaction mixture turned bright yellow. After five minutes of stirring the resulting solution was left undisturbed and the solvent was allowed to evaporate under atmospheric pressure, yielding a large crop of bright yellow crystals of **3-BAr$^F_4$·Et$_2$O** (154 mg, 57.7 µmol, 91% yield) suitable for X-ray diffraction analysis. NMR data for **3-BAr$^F_4$·Et$_2$O**: ^{1}H NMR (500 MHz, CD_2Cl_2, 297 K): δ = 7.72–7.68 (m, 18H, *o*-(3,5-C$_6$*H*$_3$(CF$_3$)$_2$) + *m*-Ar*H*), 7.56 (br, 8H, *p*-(3,5-C$_6$*H*$_3$(CF$_3$)$_2$), 7.48 (d, ^3J = 7.9 Hz, 4H, *p*-Ar*H*), 6.89 (br, 2H, N*H*), 3.44 (q, ^3J = 7.0 Hz, 8H, O(C*H*$_2$CH$_3$)$_2$), 2.53–2.45 (m, 8H, *i*Pr-C*H* + C*H*$_2$), 2.04 (dt, ^2J = 12.7 Hz, ^3J = 3.5 Hz, 4H, Cy-C*H*$_2$), 1.89–1.85 (m, 10H, Cy-C*H*$_2$), 1.59 (s, 12H, NC(C*H*$_3$)$_2$), 1.55–1.46 (m, 4H, Cy-C*H*$_2$), 1.36–1.26 (m, 2H, Cy-C*H*$_2$), 1.30 (d, ^3J = 6.7 Hz, 12H, *i*Pr-C*H*$_3$), 1.15 (t, ^3J = 7.0 Hz, 12H, O(CH$_2$C*H*$_3$)$_2$), 0.79 (d, ^3J = 6.7 Hz, 12H, *i*Pr-C*H*$_3$) ppm. ^{13}C{^{1}H} NMR

(126 MHz, CD_2Cl_2, 297 K): δ = 197.8 ($C_{carbene}$, identified by HMBC), 162.1 (1:1:1:1 q, $^1J_{BC}$ = 49.8 Hz, *i*-(3,5-$C_6H_3(CF_3)_2$)), 144.5 (*o*-Ar*C*), 135.2 (br, *o*-(3,5-$C_6H_3(CF_3)_2$)), 133.7 (*i*-Ar*C*), 130.9 (*p*-Ar*C*), 130.5 (*p*-Ar*C*), 129.3 (qq, $^2J_{CF}$ = 31.5 Hz, $^4J_{CF}$ = 2.9 Hz, *m*-(3,5-$C_6H_3(CF_3)_2$)), 128.2 (*m*-Ar*C*), 125.0 (q, $^1J_{CF}$ = 272.4 Hz, CF_3), 117.9 (sept, $^3J_{CF}$ = 4.0 Hz, *p*-(3,5-$C_6H_3(CF_3)_2$)), 85.1 (N*C*($CH_3)_2$), 66.1 (O($CH_2CH_3)_2$), 59.3 (*C*(C_5H_{10})), 45.5 (CH_2), 37.3 (Cy-CH_2), 29.9 (NC($CH_3)_2$), 29.7 (*i*Pr-*C*H), 25.5 (*i*Pr-CH_3), 24.7 (Cy-CH_2), 24.3 (*i*Pr-CH_3), 21.8 (Cy-CH_2), 15.5 (O($CH_2CH_3)_2$) ppm. *Note: the C_{NCS} resonance was not detected.* ^{11}B NMR (128 MHz, CD_2Cl_2, 297 K): δ = 33.7 (br, *B*NCS), –6.62 (s, *B*Ar^F_4) ppm. ^{19}F NMR (471 MHz, CD_2Cl_2, 297 K): δ = –62.8 (s) ppm. Solid-state IR: ν(NH) = 3396 cm^{-1}. *Note: solvent removal in vacuo and redissolving of Et_2O-free **3-BAr$^F_4$** in CD_2Cl_2 led to partial decomposition. Slow evaporation of this solution afforded a few single crystals of solvent-free **3-BAr$^F_4$** suitable for X-ray diffraction analysis.* NMR data for **3-BAr$^F_4$**: ^{1}H NMR (500 MHz, CD_2Cl_2, 297 K): δ = 7.72–7.68 (m, 18H, *o*-(3,5-$C_6H_3(CF_3)_2$) + *m*-Ar*H*), 7.56 (br, 8H, *p*-(3,5-$C_6H_3(CF_3)_2$), 7.48 (d, ^3J = 7.9 Hz, 4H, *p*-Ar*H*), 6.84 (br, 2H, N*H*), 2.53–2.45 (m, 8H, *i*Pr-C*H* + CH_2), 2.08–2.01 (m, 4H, Cy-CH_2), 1.91–1.85 (m, 10H, Cy-CH_2), 1.59 (s, 12H, NC(C$H_3)_2$), 1.57–1.44 (m, 4H, Cy-CH_2), 1.33–1.26 (m, 2H, Cy-CH_2), 1.30 (d, ^3J = 6.7 Hz, 12H, *i*Pr-CH_3), 0.79 (d, ^3J = 6.6 Hz, 12H, *i*Pr-CH_3) ppm. ^{13}C{^{1}H} NMR (126 MHz, CD_2Cl_2, 297 K): δ = 197.8 ($C_{carbene}$, identified by HMBC), 162.1 (1:1:1:1 q, $^1J_{BC}$ = 49.8 Hz, *i*-(3,5-$C_6H_3(CF_3)_2$)), 144.5 (*o*-Ar*C*), 135.2 (br, *o*-(3,5-$C_6H_3(CF_3)_2$)), 133.7 (*i*-Ar*C*), 130.9 (*p*-Ar*C*), 130.5 (*p*-Ar*C*), 129.3 (qq, $^2J_{CF}$ = 31.5 Hz, $^4J_{CF}$ = 2.9 Hz, *m*-(3,5-$C_6H_3(CF_3)_2$)), 128.2 (*m*-Ar*C*), 125.0 (q, $^1J_{CF}$ = 272.4 Hz, CF_3), 117.9 (sept, $^3J_{CF}$ = 4.0 Hz, *p*-(3,5-$C_6H_3(CF_3)_2$)), 85.1 (N*C*($CH_3)_2$), 59.3 (*C*(C_5H_{10})), 45.5 (CH_2), 37.3 (Cy-CH_2), 29.9 (NC($CH_3)_2$), 29.7 (*i*Pr-*C*H), 25.5 (*i*Pr-CH_3), 24.6 (*i*Pr-CH_3), 24.3 (Cy-CH_2), 21.8 (Cy-CH_2) ppm. *Note: the C_{NCS} resonance was not detected.* ^{11}B NMR (128 MHz, CD_2Cl_2, 297 K): δ = 33.9 (br, *B*NCS), –6.62 (s, *B*Ar^F_4) ppm. ^{19}F NMR (471 MHz, CD_2Cl_2, 297 K): δ = –62.8 (s) ppm. Elemental analysis for [$C_{112}H_{96}B_4F_{48}N_4S_2$] ($M_w$ = 2517.31): calcd. C 53.44, H 3.84, N 2.23, S 2.55%; found C 54.35, H 3.41, N 2.53, S 2.23%.

[(CAAC)(BNHCS)(BNCS)(CAAC)][Cl], 4-Cl:

Equimolar amounts of **3-Cl** (15.0 mg, 17.4 μmol) and **1'** (13.7 mg, 17.4 μmol) were combined in 0.5 mL of CD_2Cl_2, yielding an intense blue solution of **4-Cl** in analytically pure quality for NMR spectroscopic analysis. Slow evaporation of the solvent under atmospheric pressure afforded **4-Cl** (27.0 mg, 32.7 μmol, 94% yield) as a dark blue solid. ^{1}H NMR (500 MHz, CD_2Cl_2, 297 K): δ = 9.42 (br, 1H, N*H*), 7.64–7.58 (m, 2H, *p*-Ar*H*), 7.41–7.38 (m, 4H, *p*-Ar*H*), 3.18 (t, ^2J = 12.0 Hz, 2H, Cy-CH_2), 2.79–2.58 (m, 4H, *i*Pr-C*H*), 2.50–2.38 (m, 4H, Cy-CH_2 +

CH_2), 2.34 (s, 2H, CH_2), 2.01–1.63 (m, 10H, Cy-CH_2), 1.59–1.40 (m, 18H, Cy-CH_2 + NC(CH_3)$_2$), 1.31–1.23 (m, 12H, *i*Pr-CH_3), 0.98–0.86 (m, 12H, *i*Pr-CH_3) ppm. ^{13}C{^{1}H} NMR (126 MHz, CD_2Cl_2, 297 K): δ = 145.5 (*o*-Ar*C*), 145.4 (*o*-Ar*C*), 132.6 (*i*-Ar*C*), 132.0 (*p*-Ar*C*), 131.9 (*i*-Ar*C*), 131.2 (*p*-Ar*C*), 127.3 (*m*-Ar*C*), 127.1 (*m*-Ar*C*), 81.8 (N*C*(CH_3)$_2$), 79.1 (N*C*(CH_3)$_2$), 58.6 (*C*(C_5H_{10})), 58.4 (*C*(C_5H_{10})), 46.8 (*C*H_2), 45.9 (*C*H_2), 38.2 (Cy-*C*H_2), 37.6 (Cy-*C*H_2), 29.8 (NC(*C*H_3)$_2$), 29.6 (NC(*C*H_3)$_2$), 26.1 (*i*Pr-*C*H_3), 25.9 (*i*Pr-*C*H_3), 25.4 (Cy-*C*H_2), 25.0 (*i*Pr-*C*H_3), 24.6 (*i*Pr-*C*H_3), 22.8 (Cy-*C*H_2), 22.5 (Cy-*C*H_2) ppm. *Note: the $C_{carbene}$ and C_{NCS} resonances were not detected, even by HMBC.* ^{11}B NMR (128 MHz, CD_2Cl_2, 297 K): δ = 32.0 (br) ppm. *Note: both ^{11}B NMR signals overlapped.* Solid-state IR: ν(NH) = 3403 cm^{-1}. Elemental analysis for [$C_{48}H_{71}B_2ClN_4S_2$·(CH_2Cl_2)$_{0.5}$] (M_w = 867.78): calcd. C 67.13, H 8.36, N 6.46, S 7.39%; found C 67.17, H 8.42, N 6.48, S 7.31%. *Note: elemental analyses repeatedly contained 0.5 equivalents of residual CH_2Cl_2, which could not be removed as drying in vacuo resulted in partial decomposition of* ***4-Cl****.*

[(CAAC)(BNHCS)(BNCS)(CAAC)][OTf], 4-OTf:

Route A. 4-Cl (15.0 mg, 18.2 μmol) and AgOTf (4.67 mg, 18.2 μmol) were combined in 0.5 mL of CD_2Cl_2. After 1 min the resulting suspension was filtered, yielding an intense blue solution of **4-OTf** in analytically pure quality for NMR spectroscopic analysis. Slow evaporation of the solvent under atmospheric pressure afforded **4-OTf** (16.4 mg, 17.5 μmol, 96% yield) as a dark blue solid.

Route B. Equimolar amounts of **3-OTf** (10.0 mg, 9.18 μmol) and **1'** (14.5 mg, 18.4 μmol, 2.00 eq.) were combined in 0.5 mL of CD_2Cl_2. The NMR spectra of the resulting blue solution of **4-OTf** were identical with those from Route A. ^{1}H NMR (500 MHz, CD_2Cl_2, 297 K): δ = 7.69 (t, ^3J = 7.9 Hz, 1H, *p*-Ar*H*), 7.57 (t, ^3J = 7.9 Hz, 1H, *p*-Ar*H*), 7.47 (d, ^3J = 7.8 Hz, 1H, *m*-Ar*H*), 7.37 (d, ^3J = 7.8 Hz, 1H, *m*-Ar*H*), 7.13 (br, 1H, N*H*), 3.23–3.13 (m, 2H, Cy-CH_2), 2.61 (sept, ^3J = 6.6 Hz, 4H, *i*Pr-C*H*), 2.45 (s, 2H, CH_2), 2.40–2.31 (m, 3H, Cy-CH_2 + CH_2), 1.94–1.67 (m, 9H, Cy-CH_2), 1.58–1.43 (m, 18H, Cy-CH_2 + NC(CH_3)$_2$), 1.34–1.23 (m, 14H, Cy-CH_2 + *i*Pr-CH_3), 0.92 (s, 3H, *i*Pr-CH_3), 0.90 (s, 3H, *i*Pr-CH_3), 0.84 (s, 3H, *i*Pr-CH_3), 0.83 (s, 3H, *i*Pr-CH_3) ppm. ^{13}C{^{1}H} NMR (126 MHz, CD_2Cl_2, 297 K): δ = 145.4 (*o*-Ar*C*), 145.2 (*o*-Ar*C*), 132.6 (*p*-Ar*C*), 132.5 (*i*-Ar*C*), 131.8 (*i*-Ar*C*), 131.3 (*p*-Ar*C*), 127.7 (*m*-Ar*C*), 127.2 (*m*-Ar*C*), 119.9 (q, $^1J_{13C,19F}$ = 320 Hz, *C*F_3), 81.7 (N*C*(CH_3)$_2$), 79.8 (N*C*(CH_3)$_2$), 58.7 (*C*(C_5H_{10})), 58.4 (*C*(C_5H_{10})), 46.7 (*C*H_2), 46.2 (*C*H_2), 38.5 (Cy-*C*H_2), 38.0 (Cy-*C*H_2), 29.8 (NC(*C*H_3)$_2$), 29.7 (NC(*C*H_3)$_2$), 29.6 (NC(*C*H_3)$_2$), 26.0 (*i*Pr-*C*H_3), 25.6 (*i*Pr-*C*H_3), 25.4 (Cy-*C*H_2), 25.1 (*i*Pr-*C*H_3), 24.9 (*i*Pr-*C*H_3), 24.4 (Cy-*C*H_2), 22.7 (Cy-*C*H_2), 22.4 (Cy-*C*H_2) ppm. *Note: the $C_{carbene}$ and C_{NCS}*

resonances were not detected, even by HMBC. ^{11}B NMR (128 MHz, CD_2Cl_2, 297 K): δ = 32.5 (br), 31.5 (br) ppm. ^{19}F NMR (471 MHz, CD_2Cl_2, 297 K): δ = –78.8 (s) ppm. Solid-state IR: ν(NH) = 3401 cm^{-1}. Elemental analysis for [$C_{49}H_{71}B_2F_3N_4O_3S_3$·($CH_2Cl_2$)] ($M_w$ = 1023.85): calcd. C 58.66, H 7.62, N 5.97, S 10.24%; found C 58.81, H 7.22, N 5.56, S 9.74%. *Note: elemental analyses repeatedly contained one equivalent of residual CH_2Cl_2, which could not be removed as drying in vacuo resulted in partial decomposition of* ***4-OTf****.*

[(CAAC)(BNHCS)(BNCS)(CAAC)][BAr$^F_4$]·Et$_2$O, 4-BAr$^F_4$·Et$_2$O:

3-BAr$^F_4$·Et$_2$O (32.0 mg, 12.0 μmol) dissolved in 1 mL of dichloromethane was added dropwise to a suspension of **1'** (9.47 mg, 12.0 μmol) in 1 mL of Et_2O. The resulting blue solution was stirred for five minutes at room temperature. Slow evaporation of the solvent under atmospheric pressure yielded **4-BAr$^F_4$·Et$_2$O** (41.2 mg, 24.9 μmol, 94% yield) as a dark blue solid. ^{1}H NMR (500 MHz, CD_2Cl_2, 297 K): δ = 7.75–7.72 (m, 8H, *o*-(3,5-$C_6H_3(CF_3)_2$)), 7.69–7.64 (m, 1H, *p*-Ar*H*), 7.60–7.52 (m, 1H *p*-Ar*H*), 7.57 (br, 4H, *p*-(3,5-$C_6H_3(CF_3)_2$), 7.46 (d, ^3J = 7.8 Hz, 2H, *m*-Ar*H*), 7.36 (d, ^3J = 7.8 Hz, 2H, *m*-Ar*H*), 6.88 (br, 1H, N*H*), 3.44 (q, ^3J = 7.0 Hz, 4H, O(CH_2CH$_3$)$_2$), 3.21–3.16 (m, 2H, Cy-CH_2), 2.64–2.56 (m, 4H, *i*Pr-C*H*), 2.47–2.34 (m, 6H, Cy-CH_2 + CH_2), 1.97–1.69 (m, 10H, Cy-CH_2 + CH_2), 1.60–1.42 (m, 18H, Cy-CH_2 + NC(CH_3)$_2$), 1.30–1.25 (m, 12H, *i*Pr-CH_3), 1.16 (t, ^3J = 7.0 Hz, 6H, O(CH$_2$CH_3)$_2$), 0.92–0.87 (m, 6H, *i*Pr-CH_3), 0.82 (d, ^3J = 6.6 Hz, 12H, *i*Pr-CH_3) ppm. ^{13}C{^{1}H} NMR (126 MHz, CD_2Cl_2, 297 K): δ = 188.0 ($C_{carbene}$, identified by HMBC), 162.2 (1:1:1:1 q, $^1J_{BC}$ = 49.9 Hz, *i*-(3,5-*C*$_6$H$_3$(CF$_3$)$_2$)), 145.5 (*o*-Ar*C*), 145.1 (*o*-Ar*C*), 135.2 (br, *o*-(3,5-*C*$_6$H$_3$(CF$_3$)$_2$)), 132.7 (*p*-Ar*C*), 132.5 (*i*-Ar*C*), 131.8 (*i*-Ar*C*), 131.3 (*p*-Ar*C*), 129.3 (qq, $^2J_{CF}$ = 31.5 Hz, $^4J_{CF}$ = 2.9 Hz, *m*-(3,5-*C*$_6$H$_3$(CF$_3$)$_2$)), 127.8 (*m*-Ar*C*), 127.2 (*m*-Ar*C*), 125.0 (q, $^1J_{CF}$ = 272.4 Hz, *C*F$_3$), 117.9 (sept, $^3J_{CF}$ = 4.0 Hz, *p*-(3,5-*C*$_6$H$_3$(CF$_3$)$_2$)), 81.5 (N*C*(CH$_3$)$_2$), 80.0 (N*C*(CH$_3$)$_2$), 66.1 (O(*C*H$_2$CH$_3$)$_2$), 58.8 (*C*(C$_5$H$_{10}$)), 58.4 (*C*(C$_5$H$_{10}$)), 46.7 (*C*H$_2$), 46.3 (*C*H$_2$), 38.7 (Cy-*C*H$_2$), 38.0 (Cy-*C*H$_2$), 30.2 (*i*Pr-*C*H), 29.8 (NC(*C*H$_3$)$_2$), 29.8 (NC(*C*H$_3$)$_2$), 29.7 (NC(*C*H$_3$)$_2$), 29.2 (*i*Pr-*C*H), 26.5 (*i*Pr-*C*H$_3$), 26.0 (*i*Pr-*C*H$_3$), 25.6 (*i*Pr-*C*H$_3$), 25.4 (Cy-*C*H$_2$), 25.1 (Cy-*C*H$_2$), 24.9 (*i*Pr-*C*H$_3$), 24.6 (*i*Pr-*C*H$_3$), 24.3 (*i*Pr-*C*H$_3$), 22.8 (Cy-*C*H$_2$), 22.4 (Cy-*C*H$_2$), 22.0 (Cy-*C*H$_2$), 21.6 (Cy-*C*H$_2$), 15.5 (O(CH$_2$*C*H$_3$)$_2$) ppm. *Note: the C_{NCS} resonance was not detected.* ^{11}B NMR (128 MHz, CD_2Cl_2, 297 K): δ = 32.2 (br, *B*NCS), 31.2 (br, *B*NCS), –6.61 (s, *B*Ar$^F_4$) ppm. ^{19}F NMR (471 MHz, CD_2Cl_2, 297 K): δ = –62.8 (s) ppm. Solid-state IR: ν(NH) = 3401 cm^{-1}. Elemental analysis for [$C_{80}H_{83}B_3F_{24}N_4S_2$] ($M_w$ = 1653.08): calcd. C 58.13, H 5.06, N 3.39, S 3.88%; found C 57.67, H 5.54, N 3.43, S 3.32%.

NMR spectra of isolated compounds

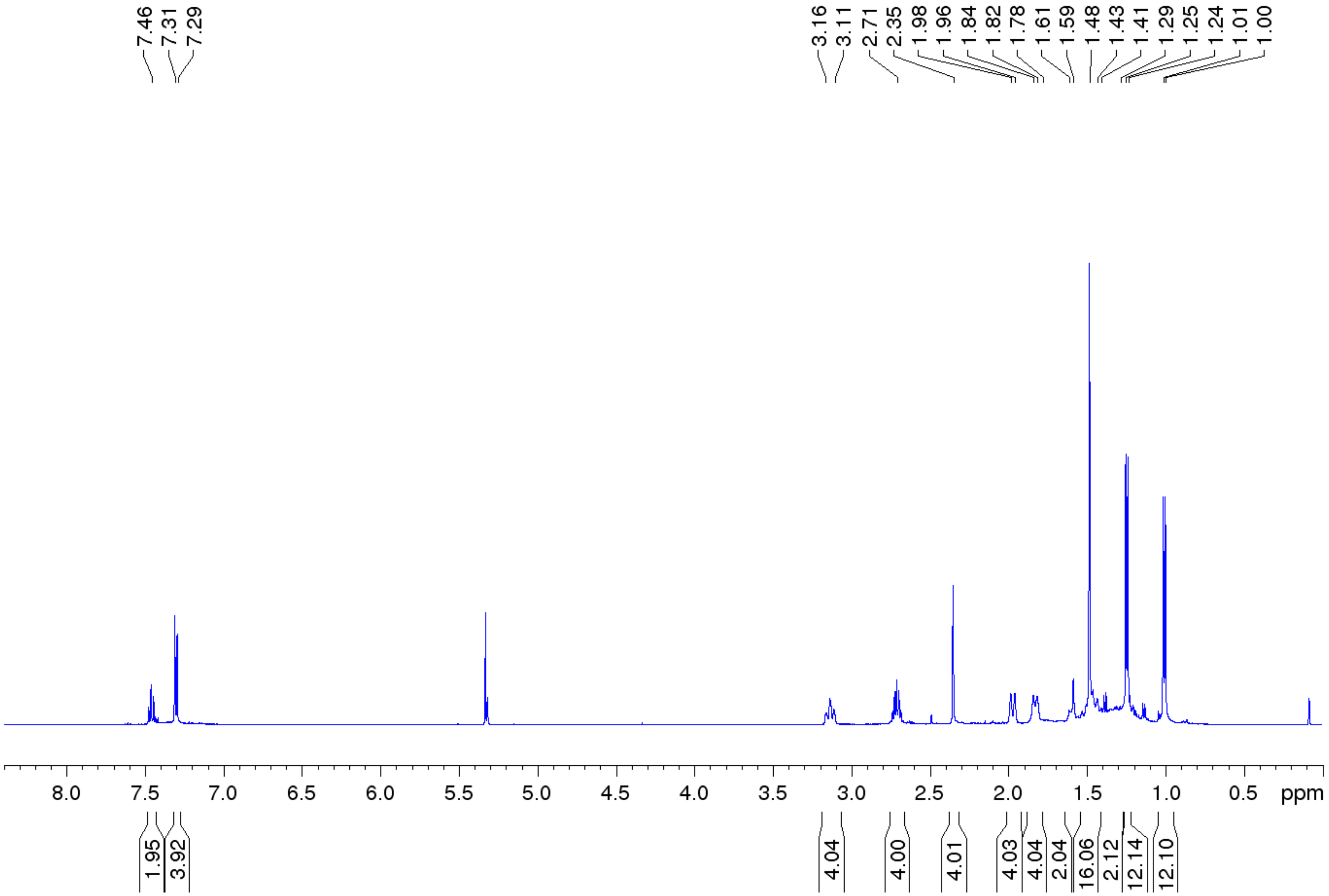

Figure S1. ^{1}H NMR spectrum of **2** in CD_2Cl_2.

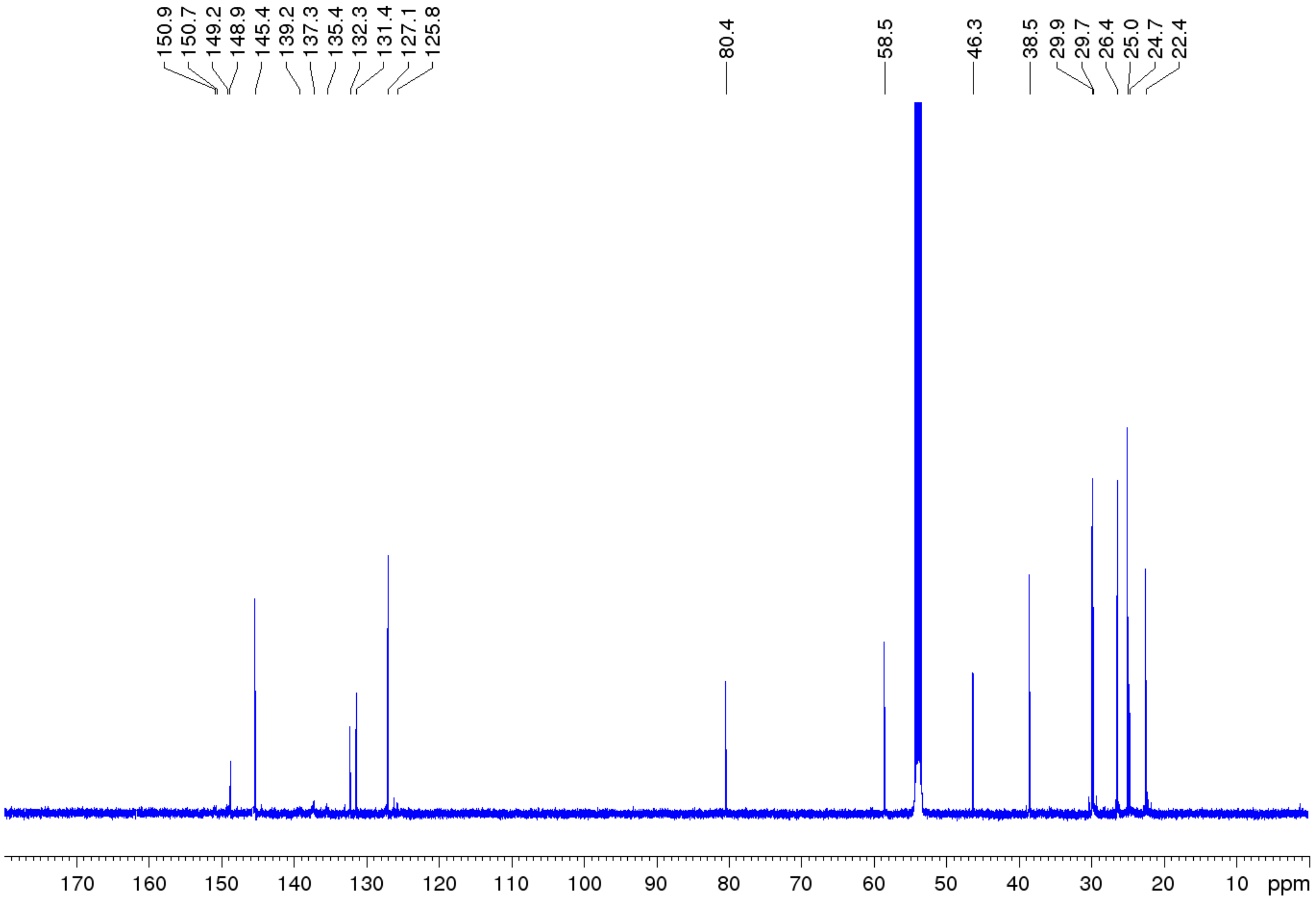

Figure S2. $^{13}C\{^1H\}$ NMR spectrum of **2** in CD_2Cl_2.

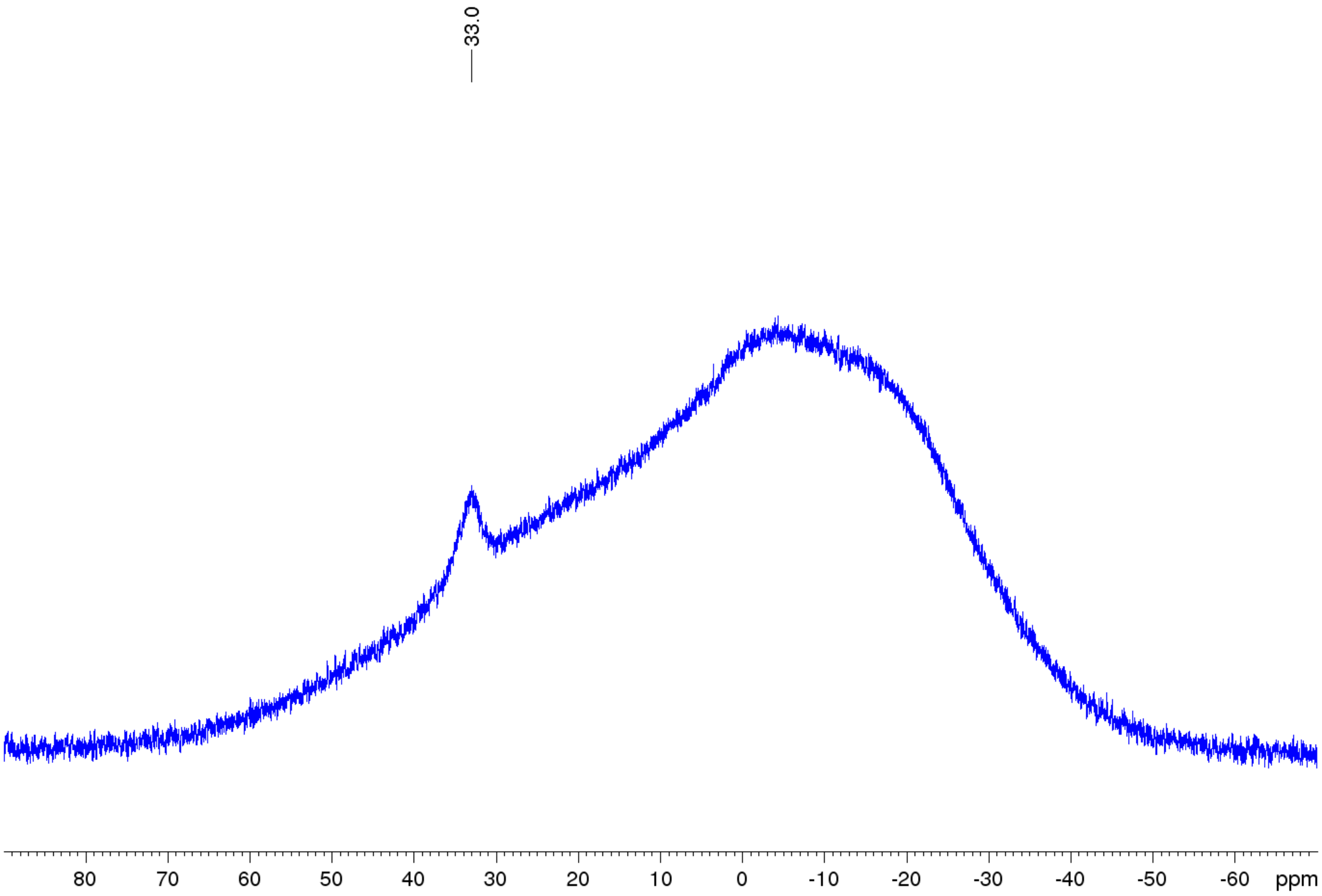

Figure S3. ^{11}B NMR spectrum of **2** in CD_2Cl_2.

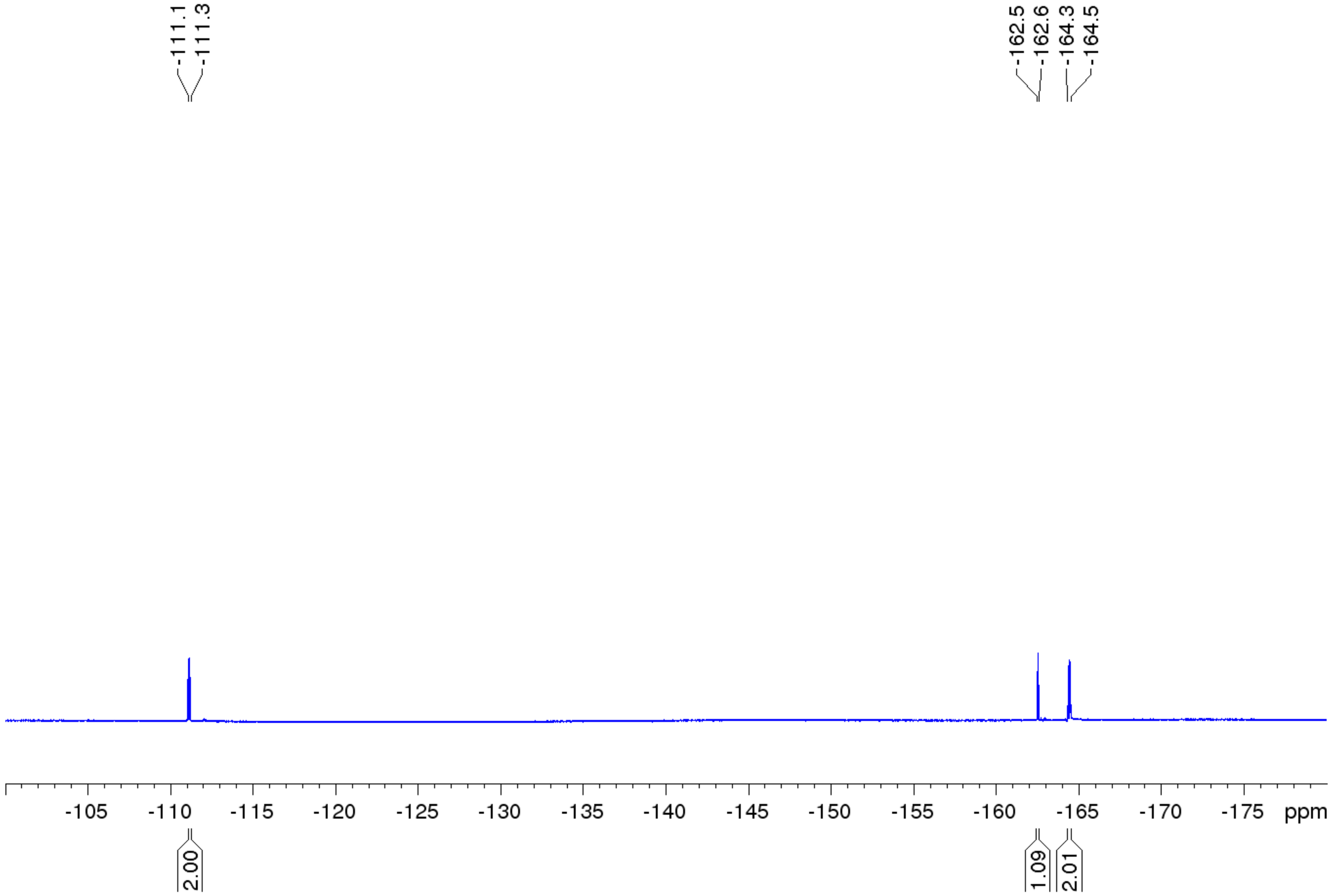

Figure S4. ^{19}F NMR spectrum of **2** in CD_2Cl_2.

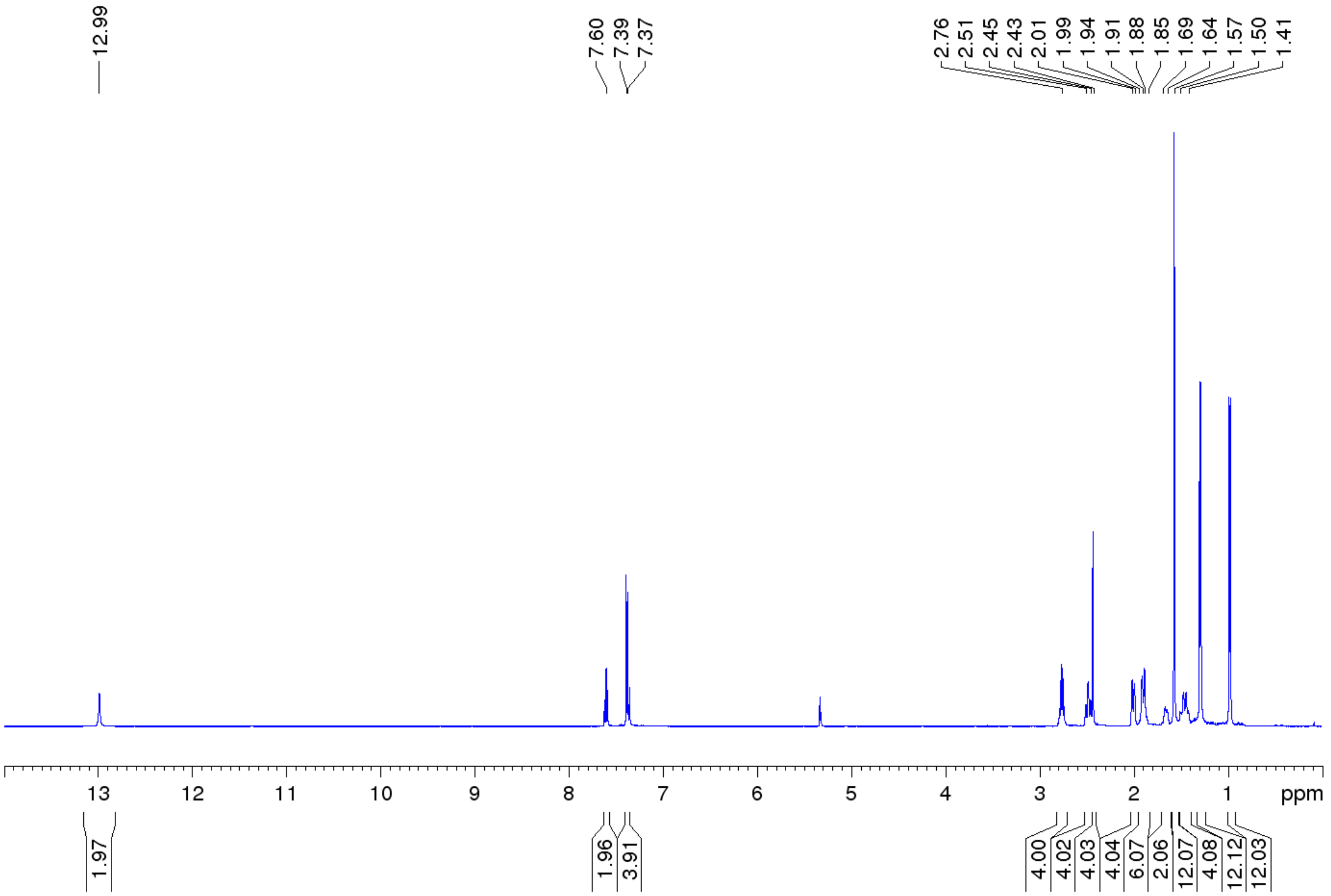

Figure S5. ^{1}H NMR spectrum of **3-Cl** in CD_2Cl_2.

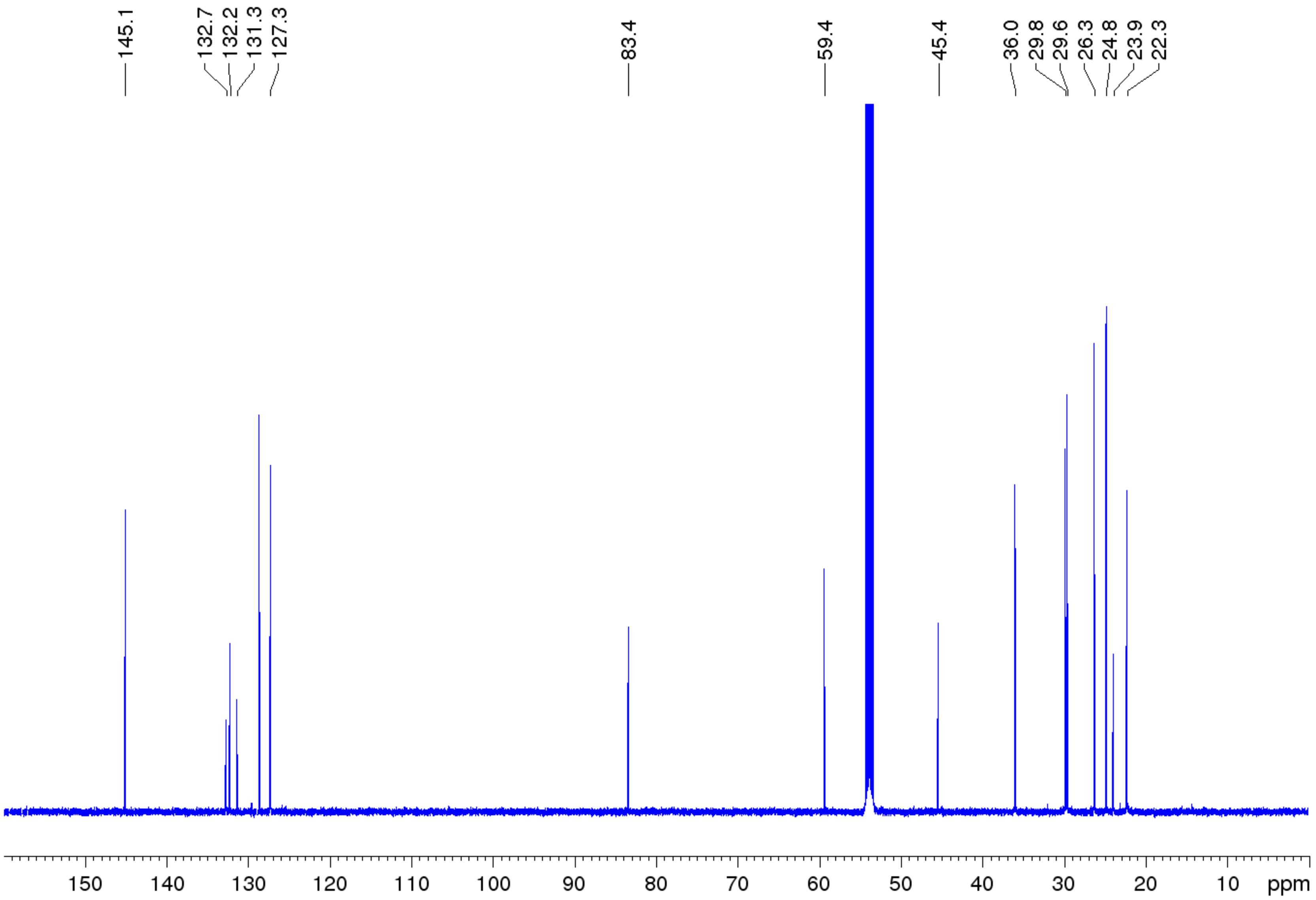

Figure S6. $^{13}C\{^1H\}$ NMR spectrum of **3-Cl** in CD_2Cl_2.

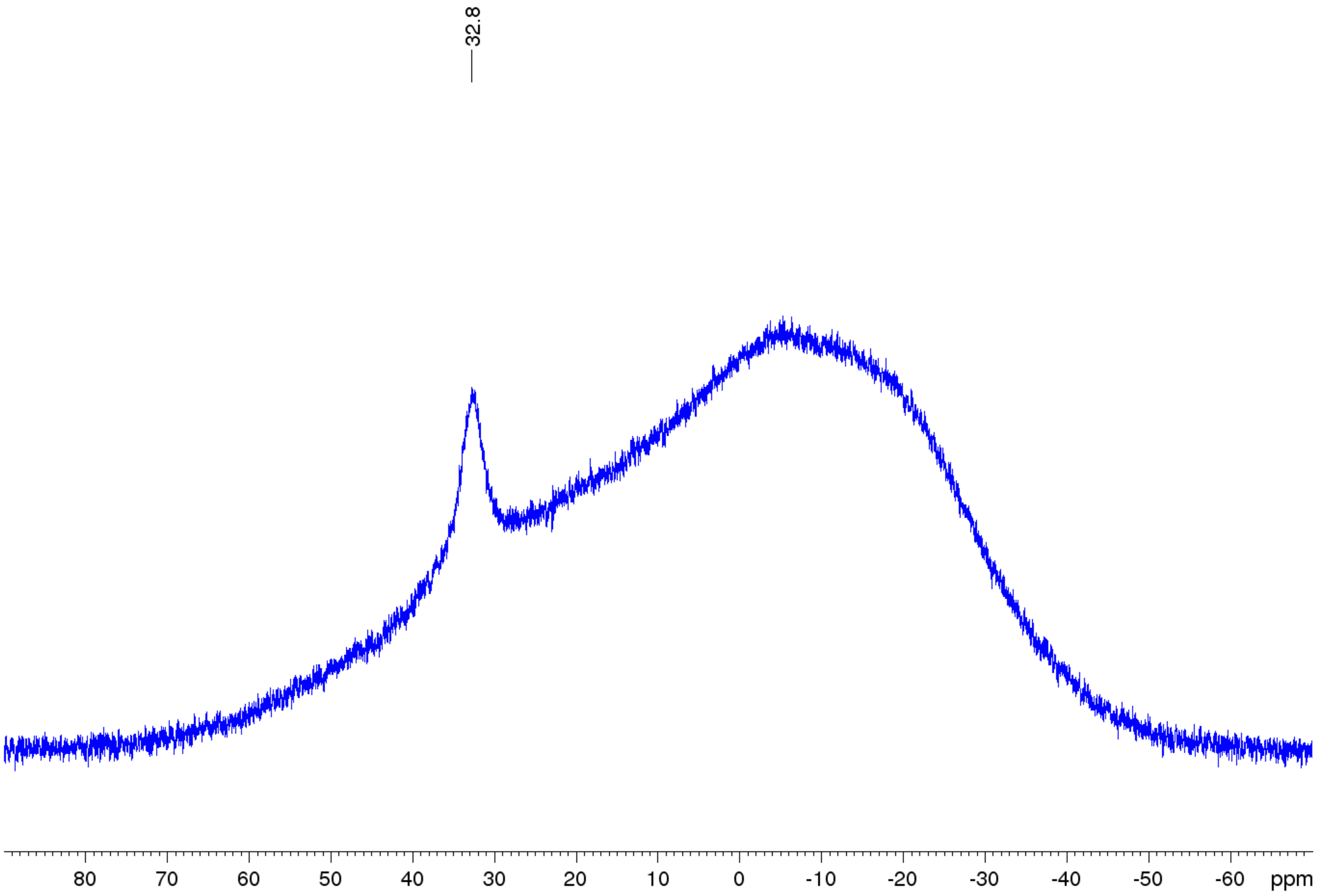

Figure S7. ^{11}B NMR spectrum of **3-Cl** in CD_2Cl_2.

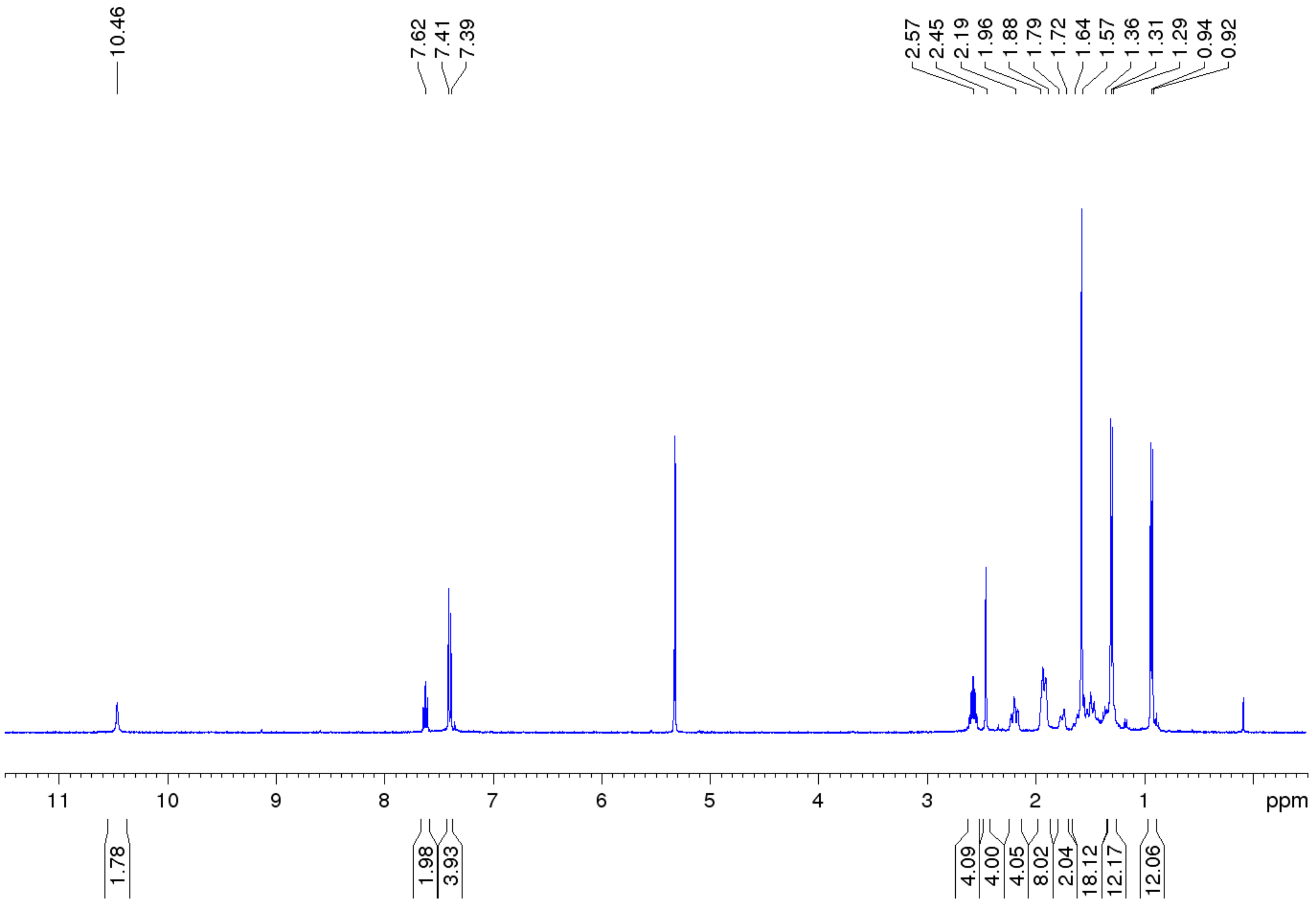

Figure S8. ^{1}H NMR spectrum of **3-OTf** in CD_2Cl_2.

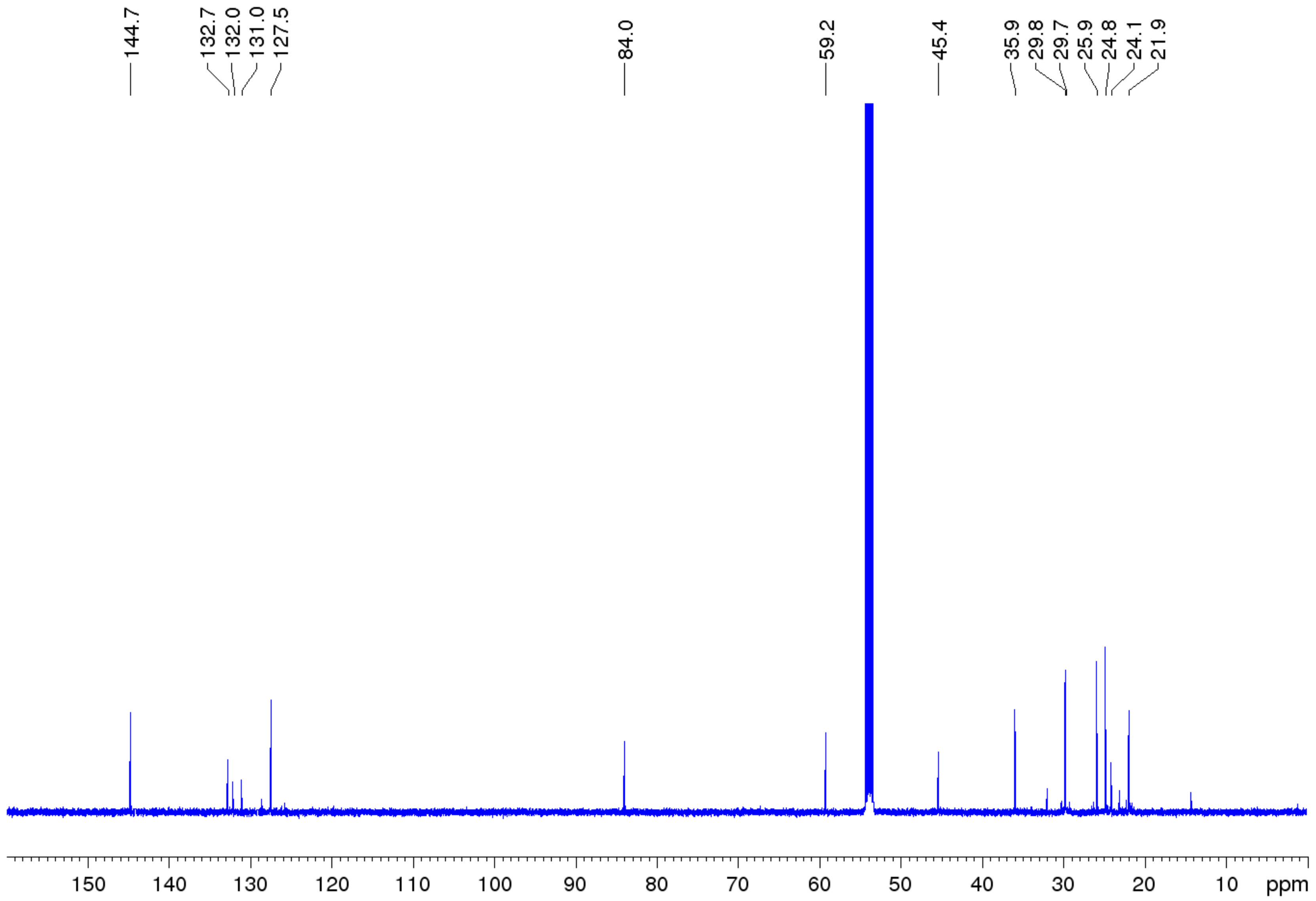

Figure S9. $^{13}C\{^{1}H\}$ NMR spectrum of **3-OTf** in CD_2Cl_2.

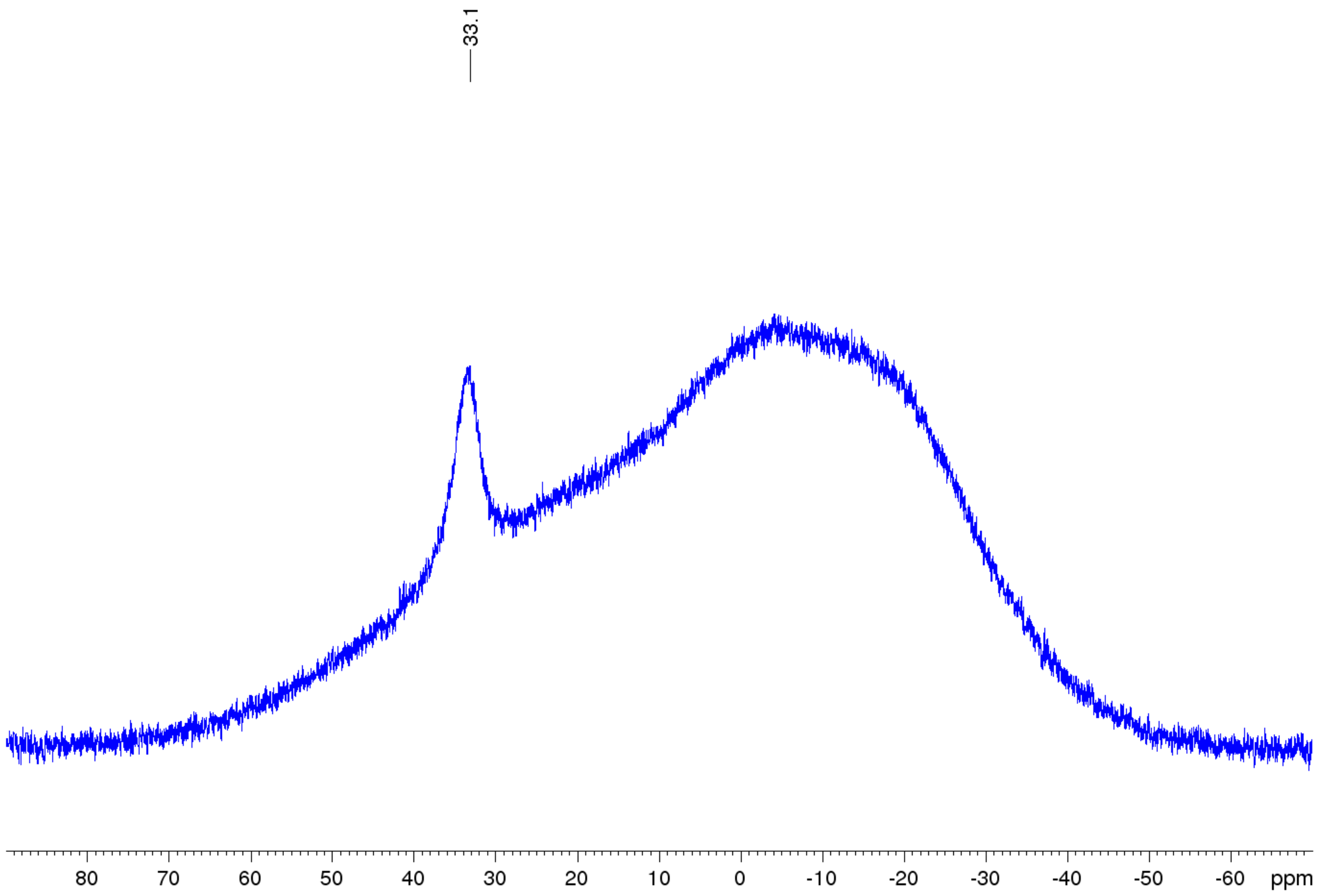

Figure S10. ^{11}B NMR spectrum of **3-OTf** in CD_2Cl_2.

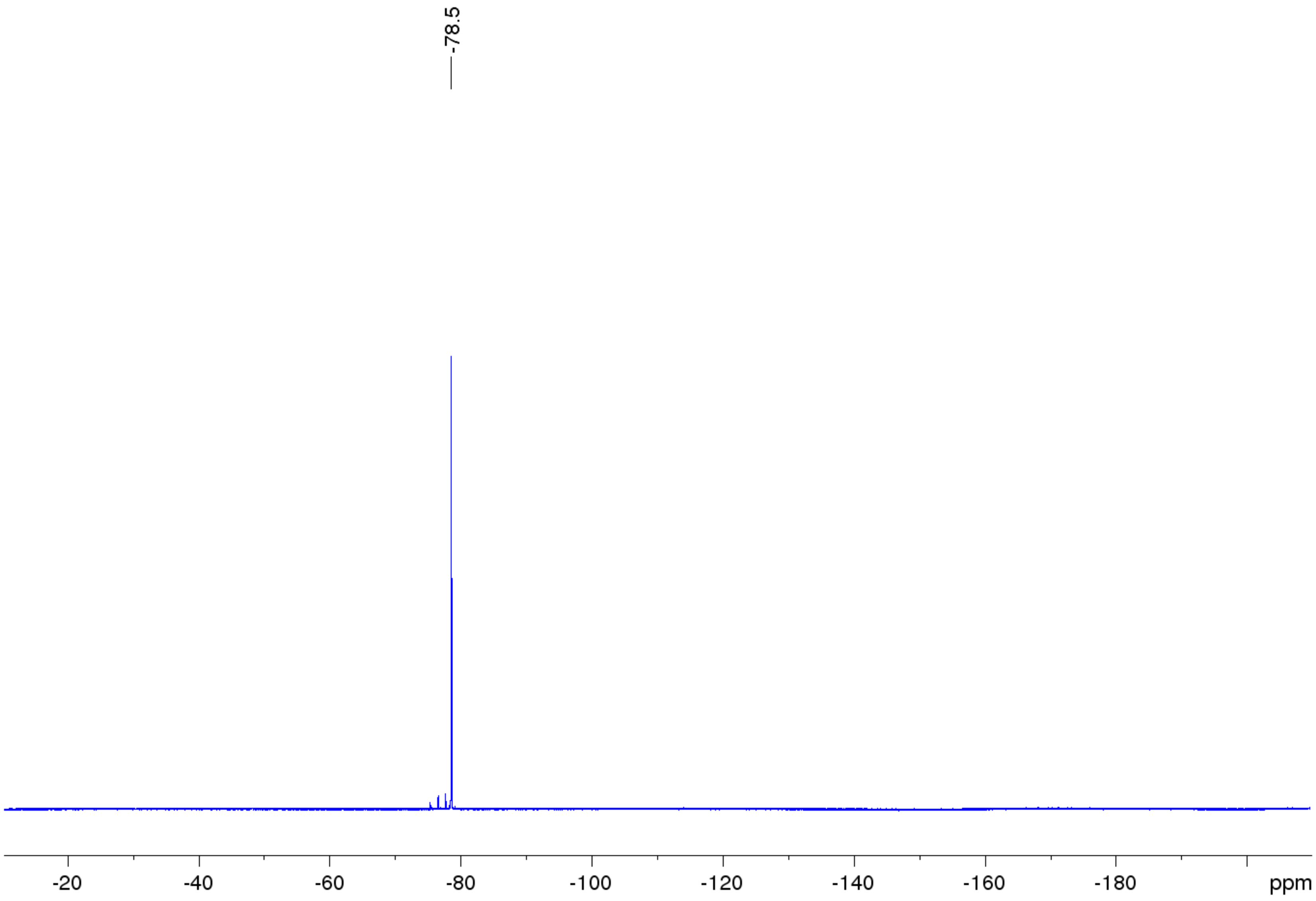

Figure S11. ^{19}F NMR spectrum of **3-OTf** in CD_2Cl_2.

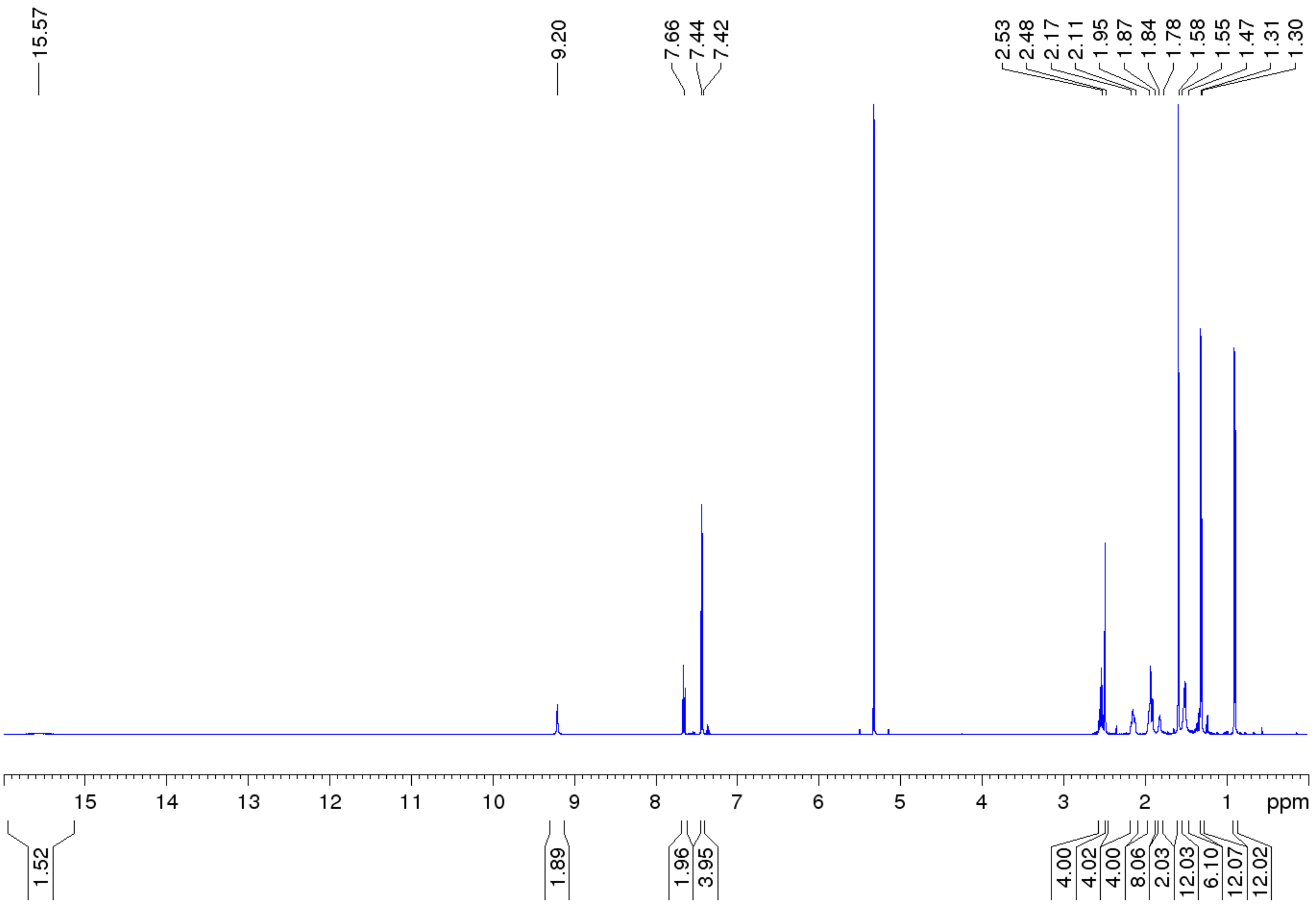

Figure S12. ^{1}H NMR spectrum of **3-OTf·HOTf** in CD_2Cl_2.

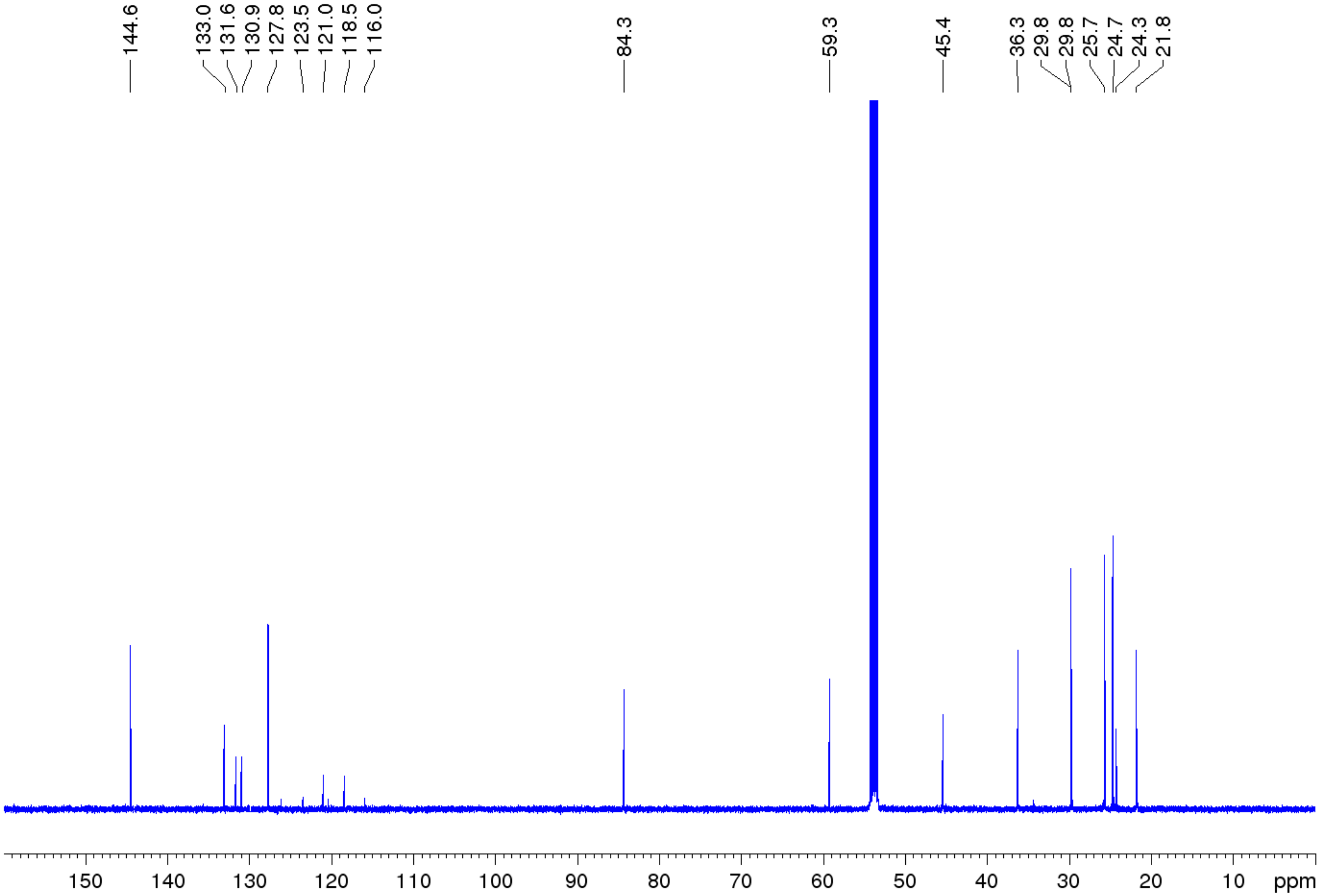

Figure S13. $^{13}C\{^1H\}$ NMR spectrum of **3-OTf·HOTf** in CD_2Cl_2.

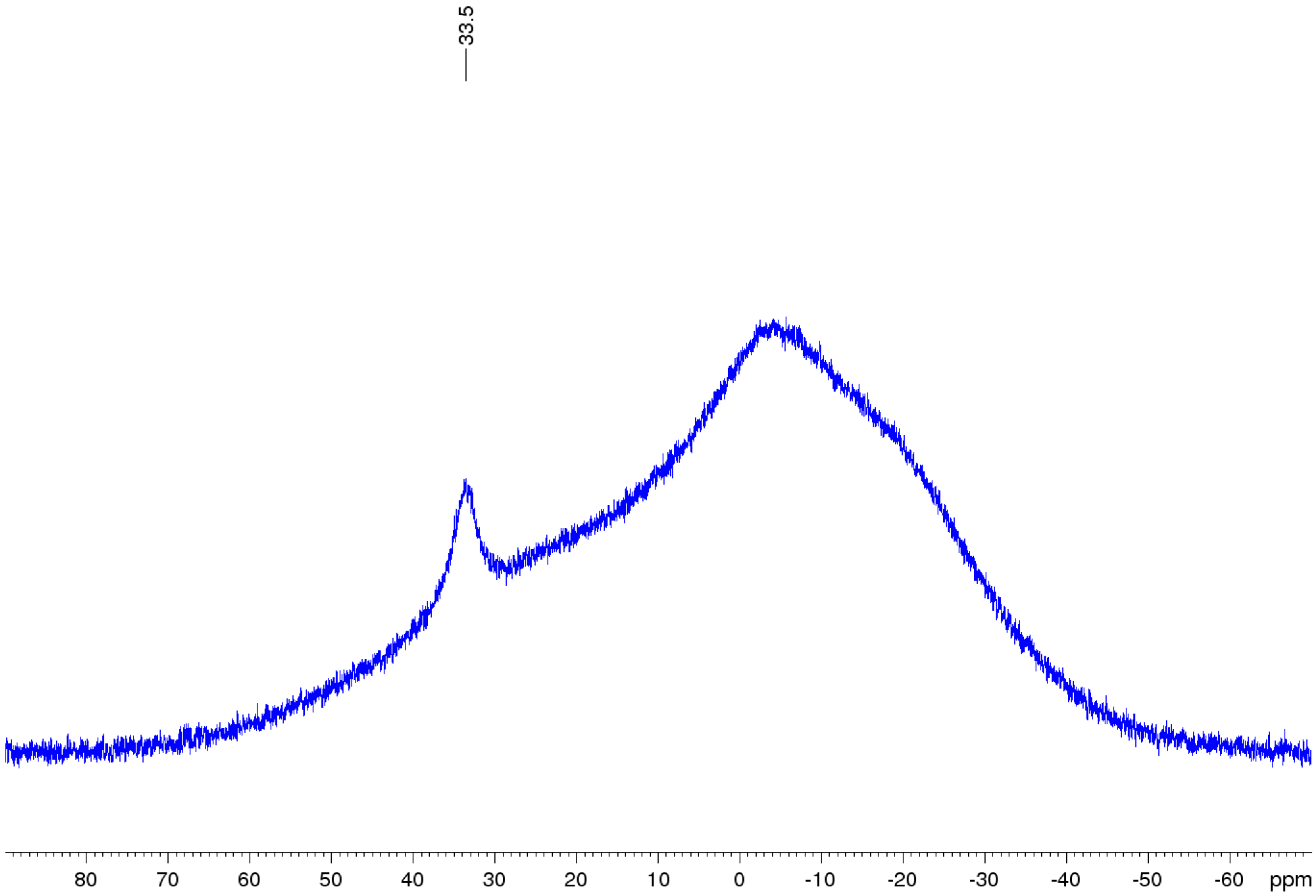

Figure S14. ^{11}B NMR spectrum of **3-OTf·HOTf** in CD_2Cl_2.

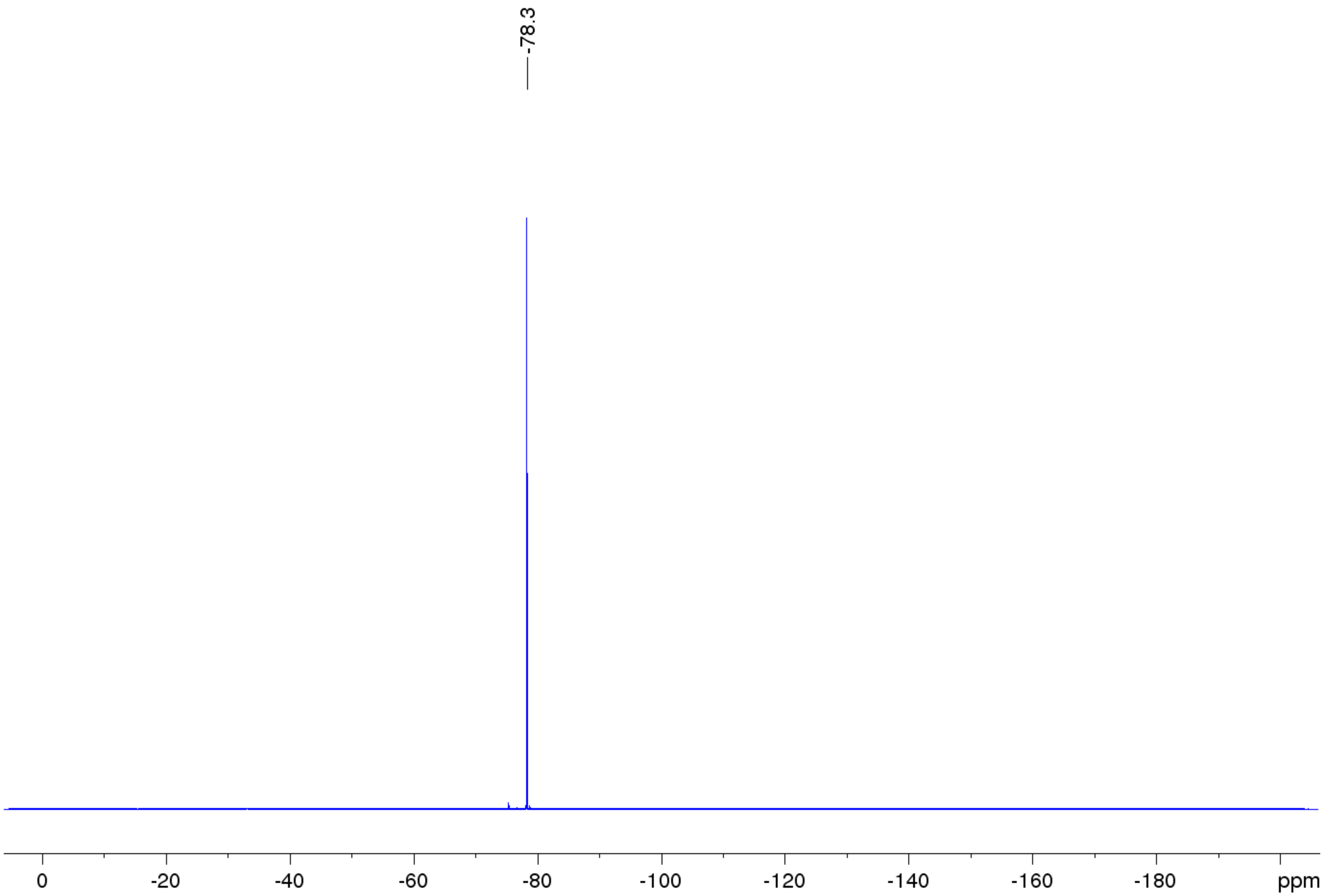

Figure S15. ^{19}F NMR spectrum of **3-OTf·HOTf** in CD_2Cl_2.

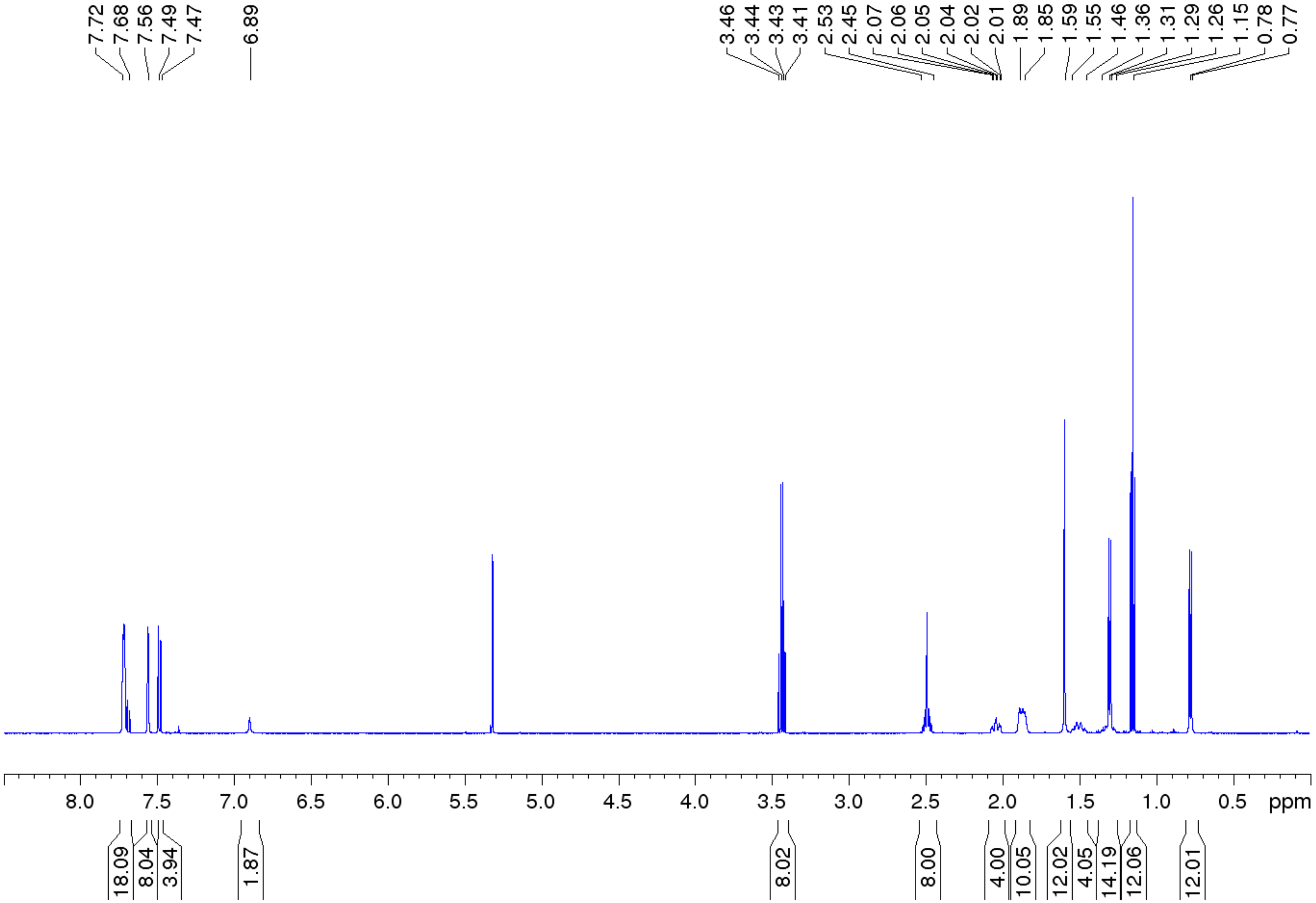

Figure S16. ^{1}H NMR spectrum of **3-BAr^{F}$_4$·Et$_2$O** in CD_2Cl_2.

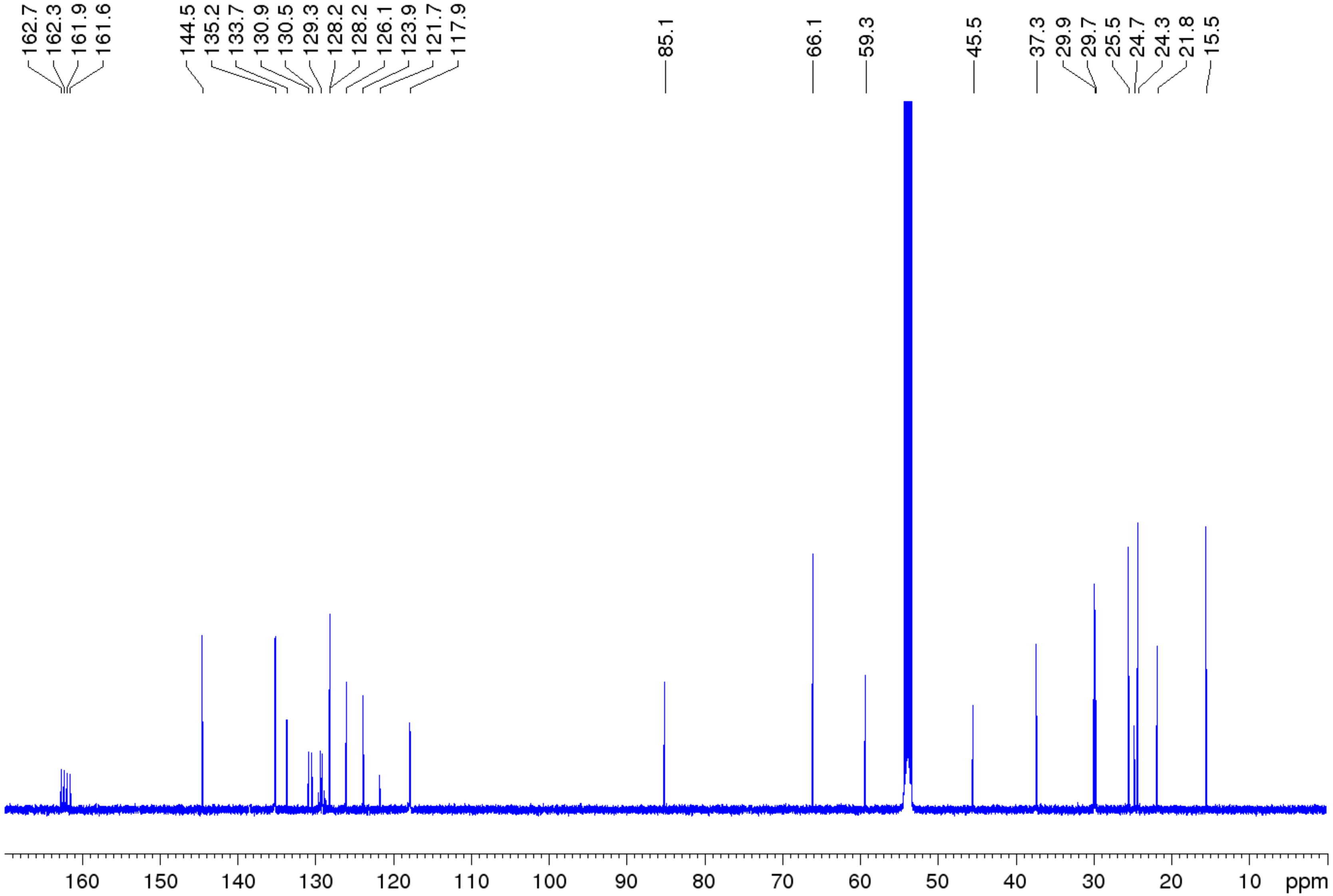

Figure S17. $^{13}C\{^{1}H\}$ NMR spectrum of **3-BAr$^{F}_{4}$·Et$_2$O** in CD_2Cl_2.

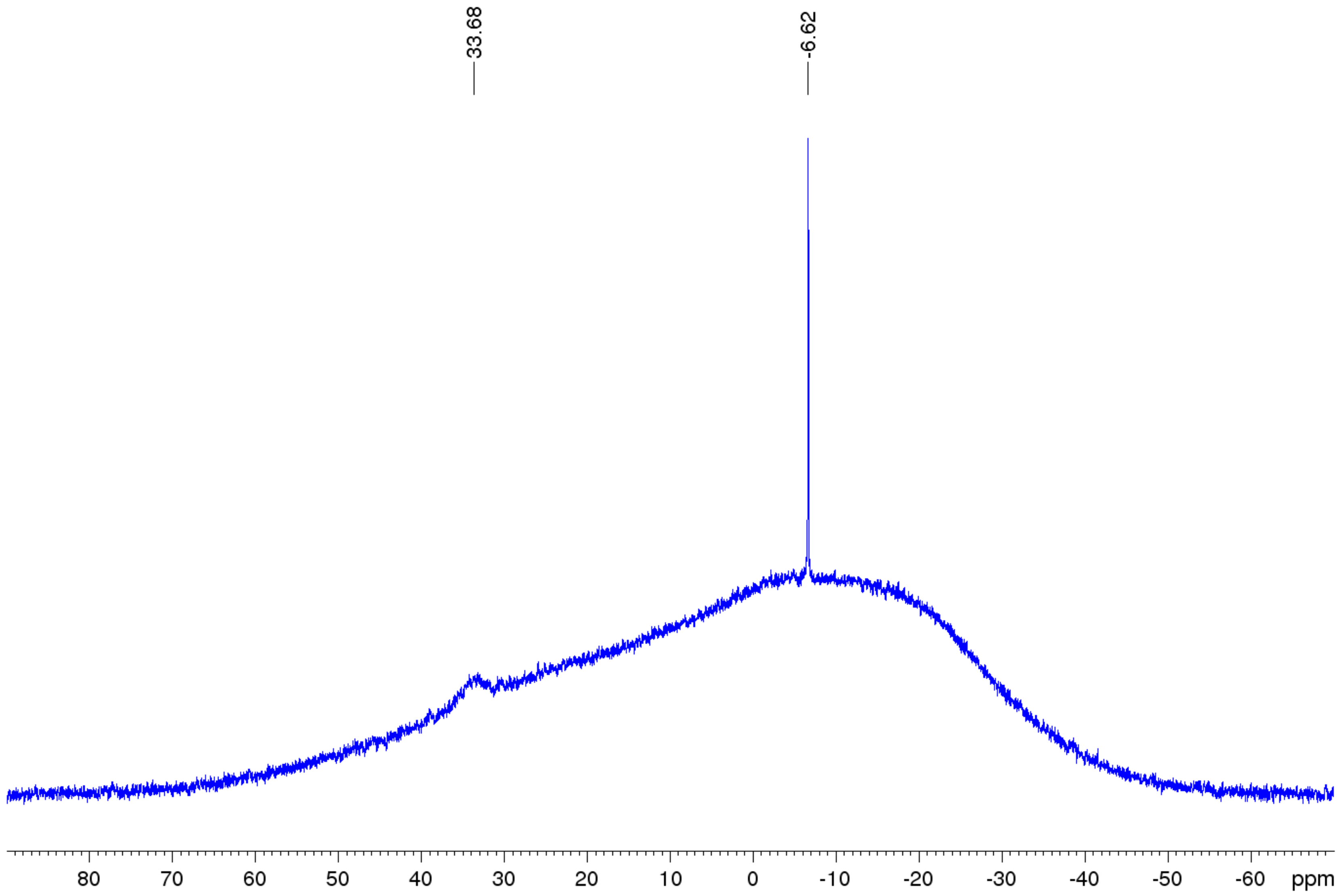

Figure S18. ^{11}B NMR spectrum of **3-BAr$^{F}{}_{4}$·Et$_{2}$O** in CD_2Cl_2.

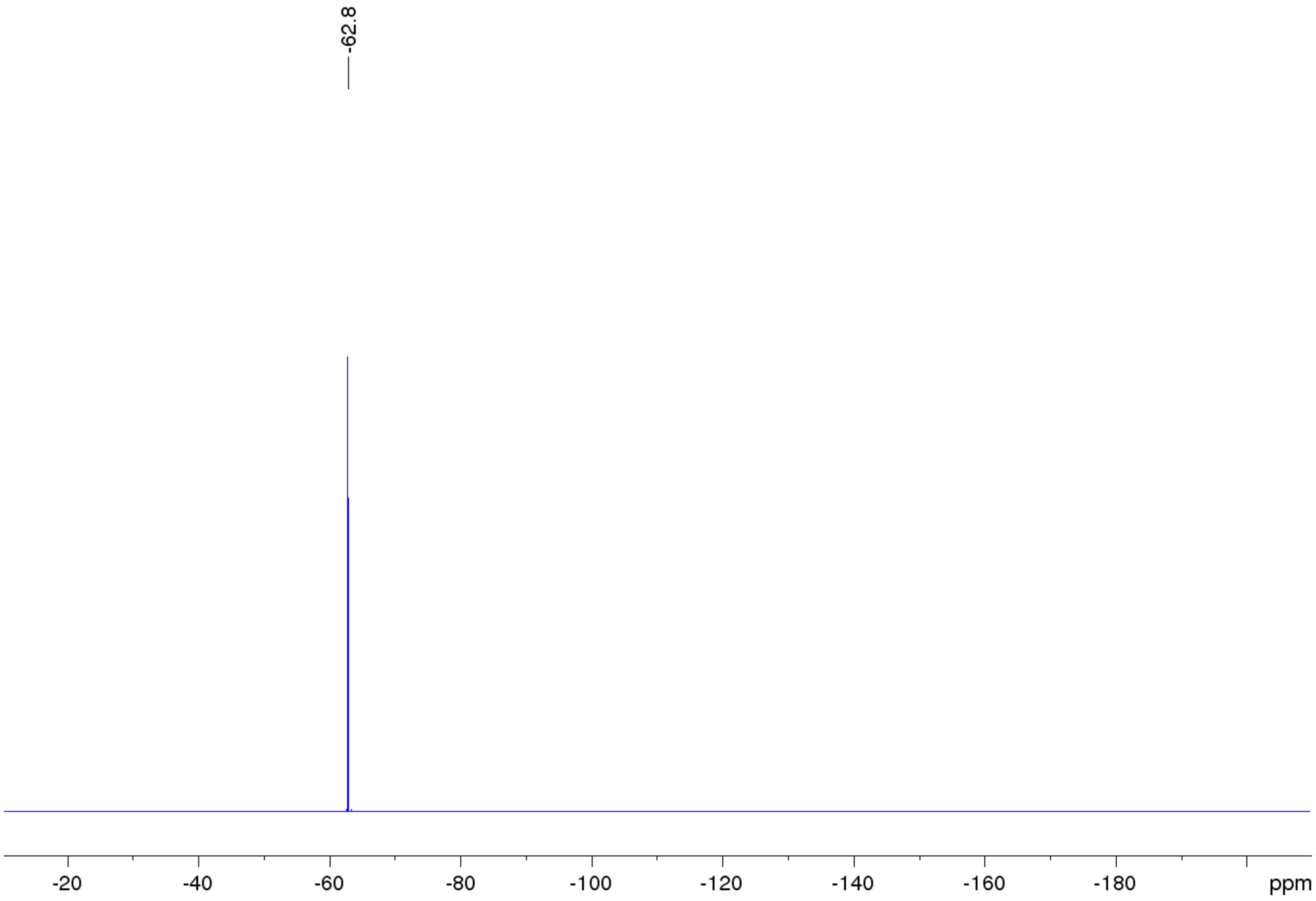

Figure S19. ^{19}F NMR spectrum of **3-BAr^{F}$_4$·Et$_2$O** in CD_2Cl_2.

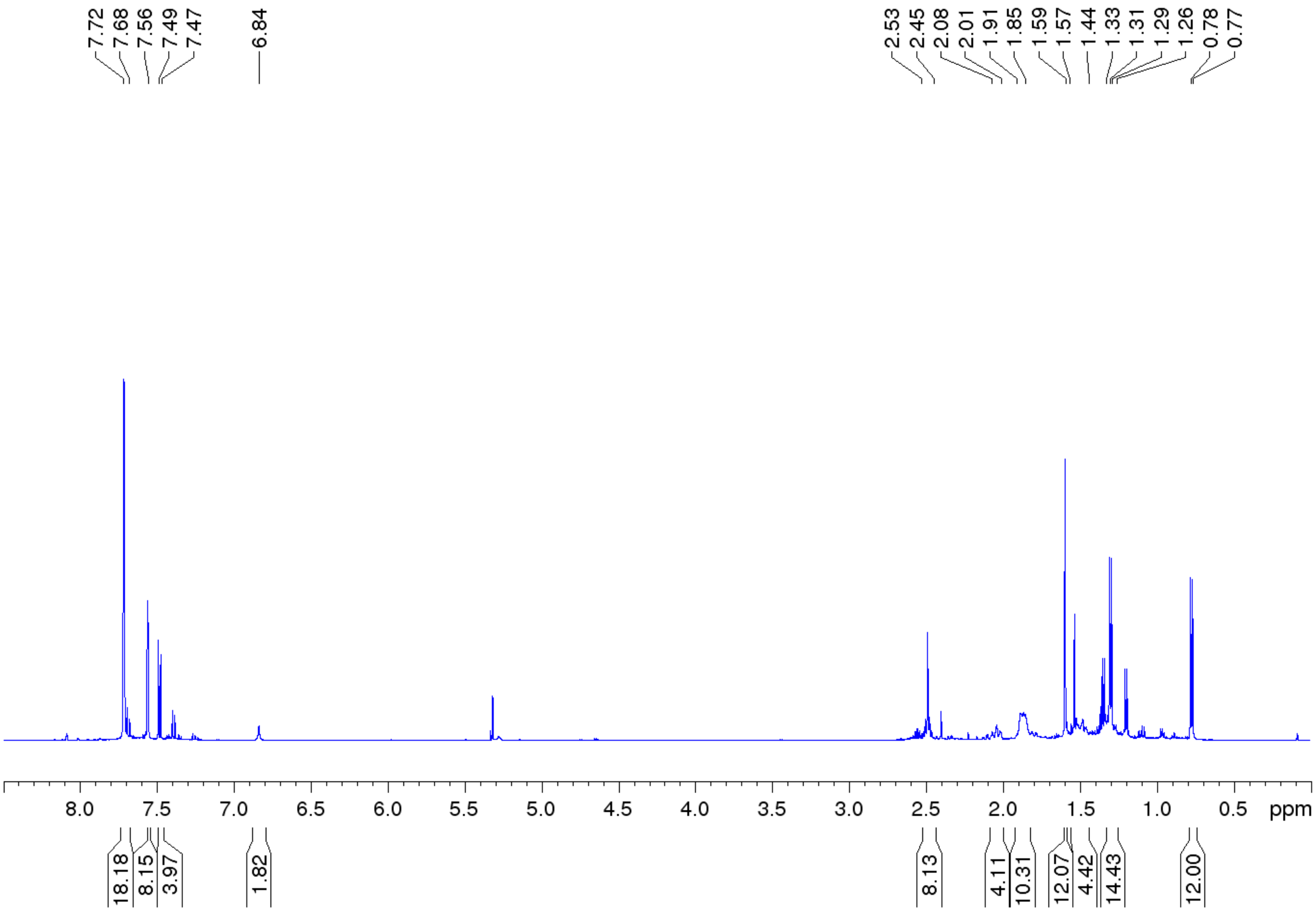

Figure S20. ^{1}H NMR spectrum of **3-BAr^{F}$_{4}$** in CD_2Cl_2. The additional resonances belong to an unidentified decomposition product.

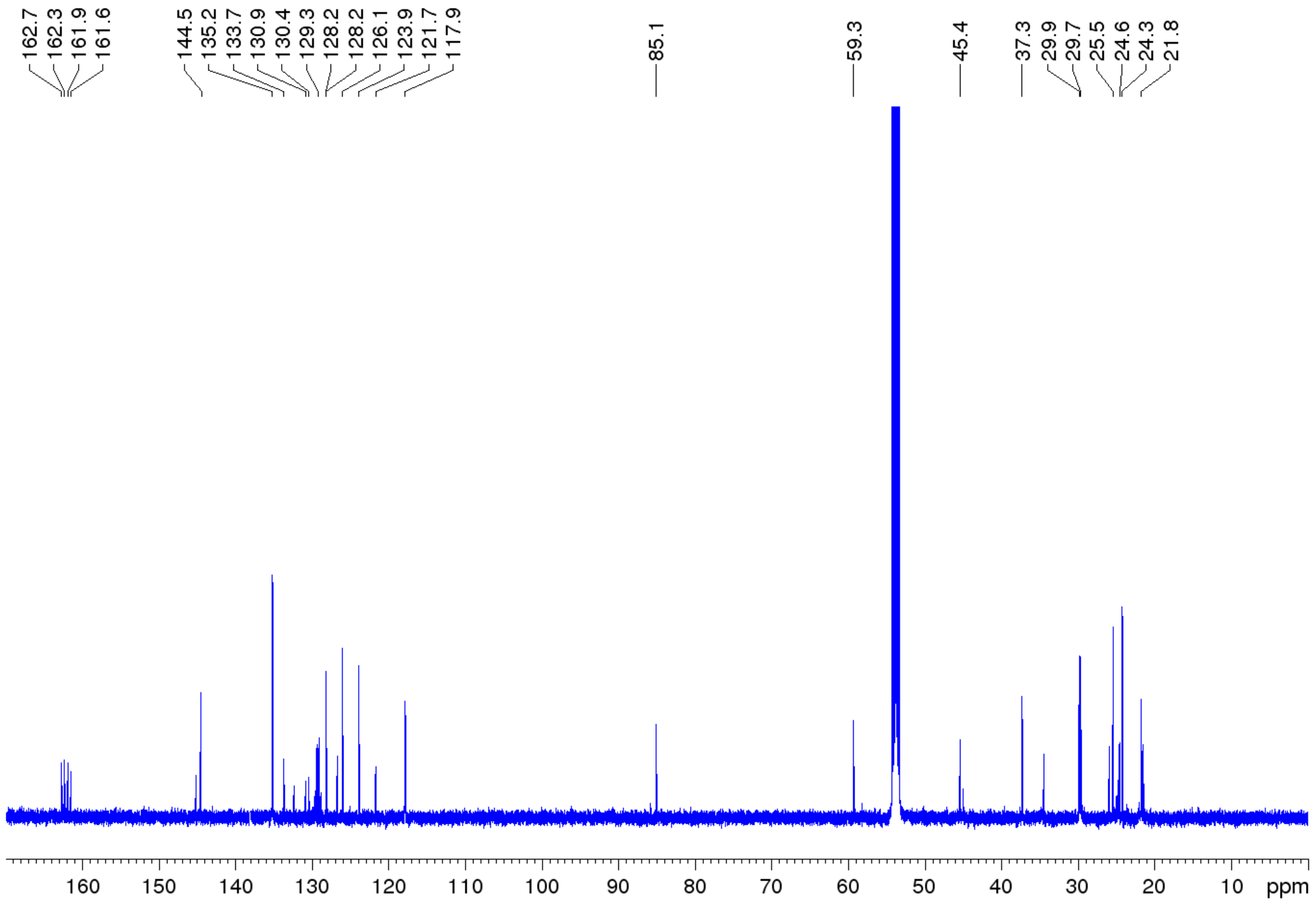

Figure S21. $^{13}C\{^1H\}$ NMR spectrum of **3-BAr^F$_4$** in CD_2Cl_2. The additional resonances belong to an unidentified decomposition product.

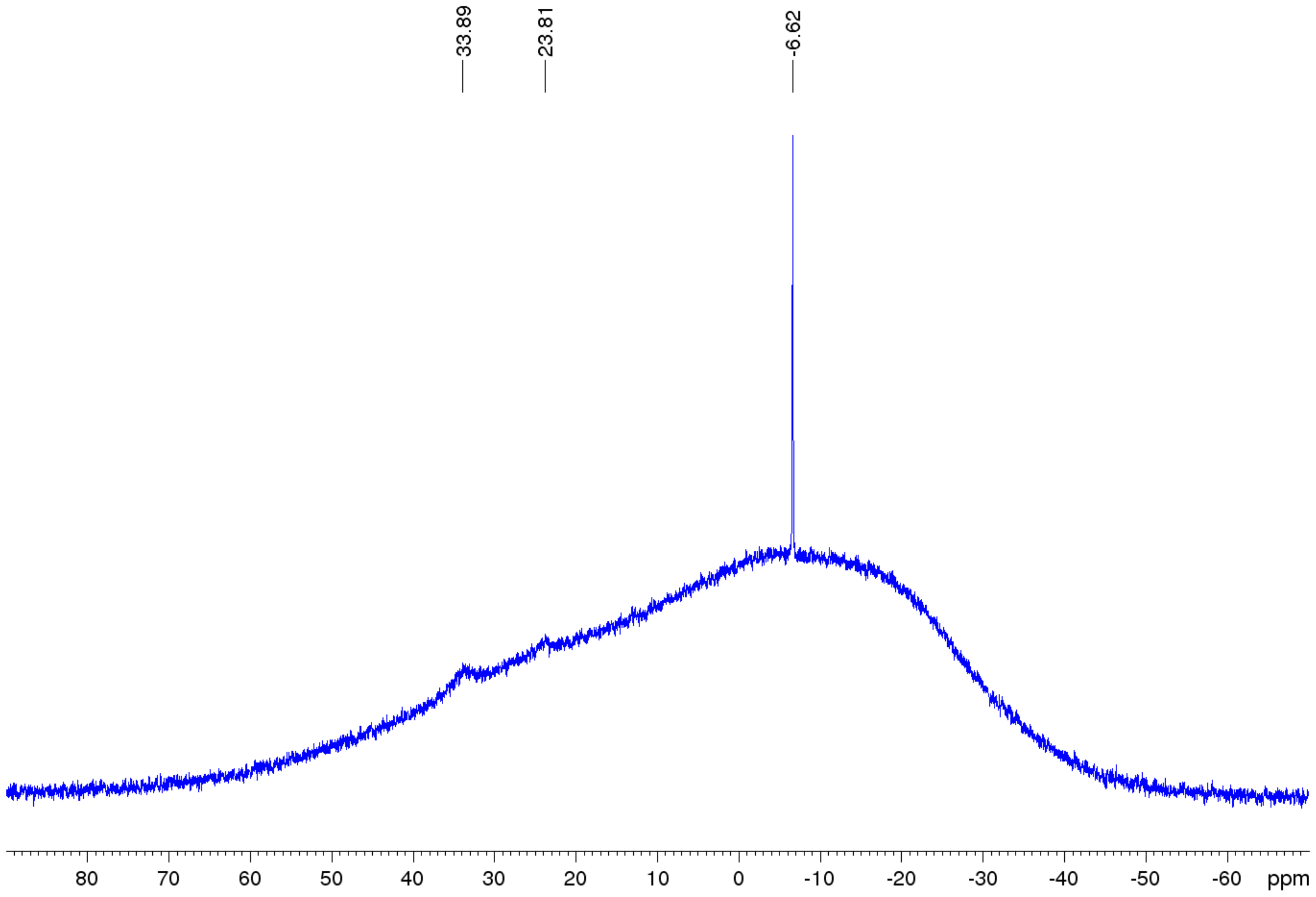

Figure S22. ^{11}B NMR spectrum of **3-BAr$^{F}_{4}$** in CD_2Cl_2. The additional resonance at 23.8 ppm belongs to an unidentified decomposition product.

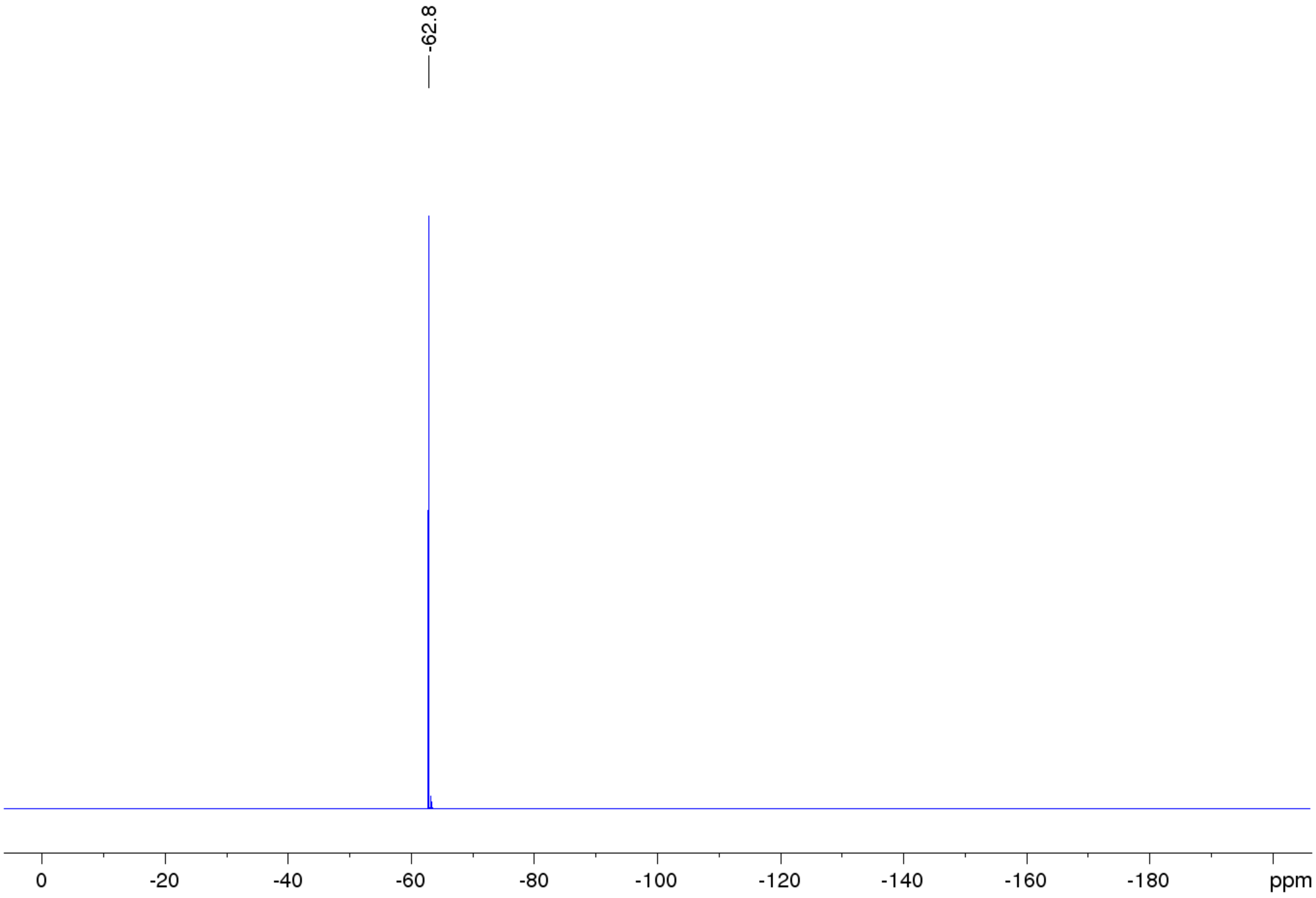

Figure S23. ^{19}F NMR spectrum of **3-BAr$^{F}_{4}$** in CD_2Cl_2.

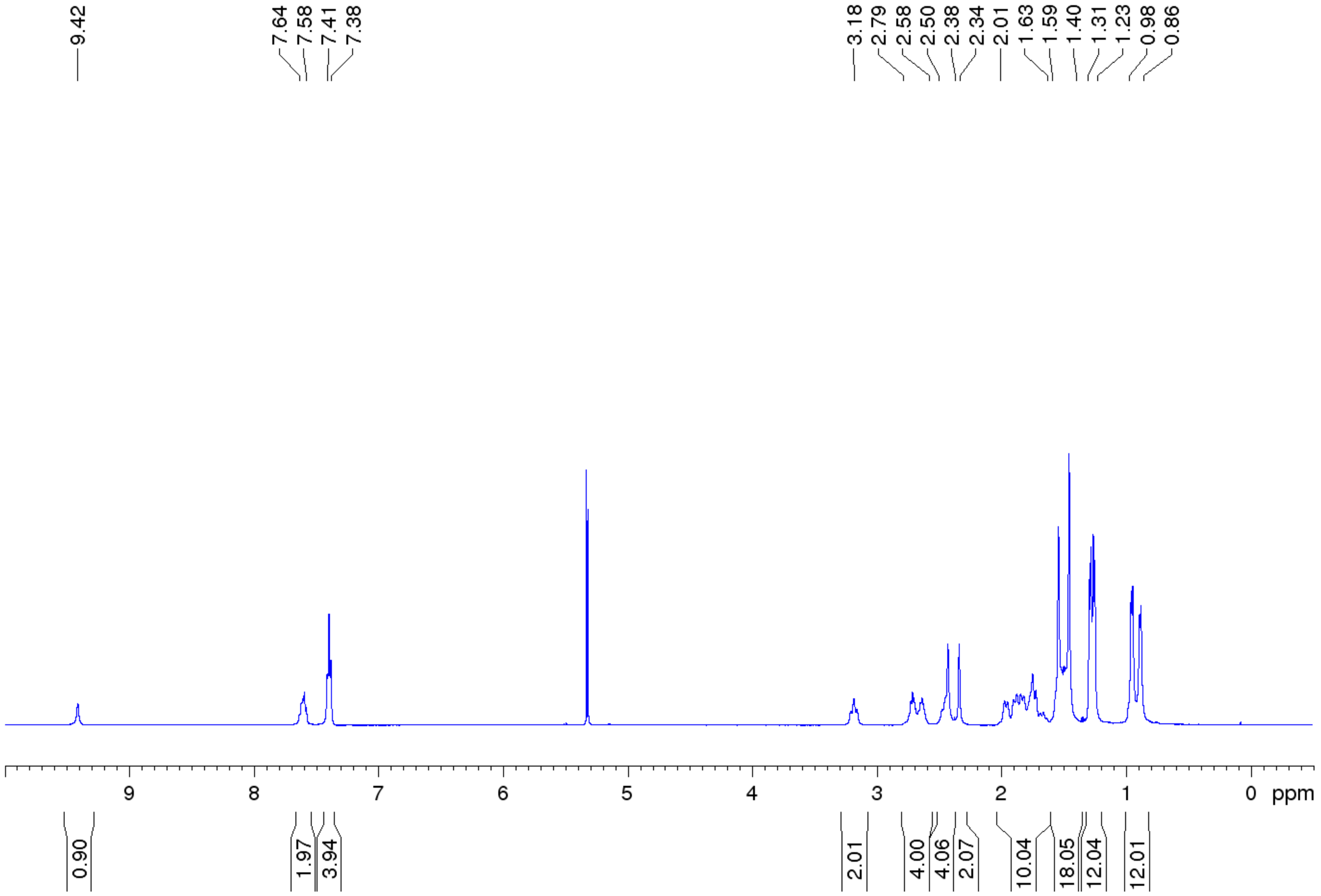

Figure S24. ^{1}H NMR spectrum of **4-Cl** in CD_2Cl_2.

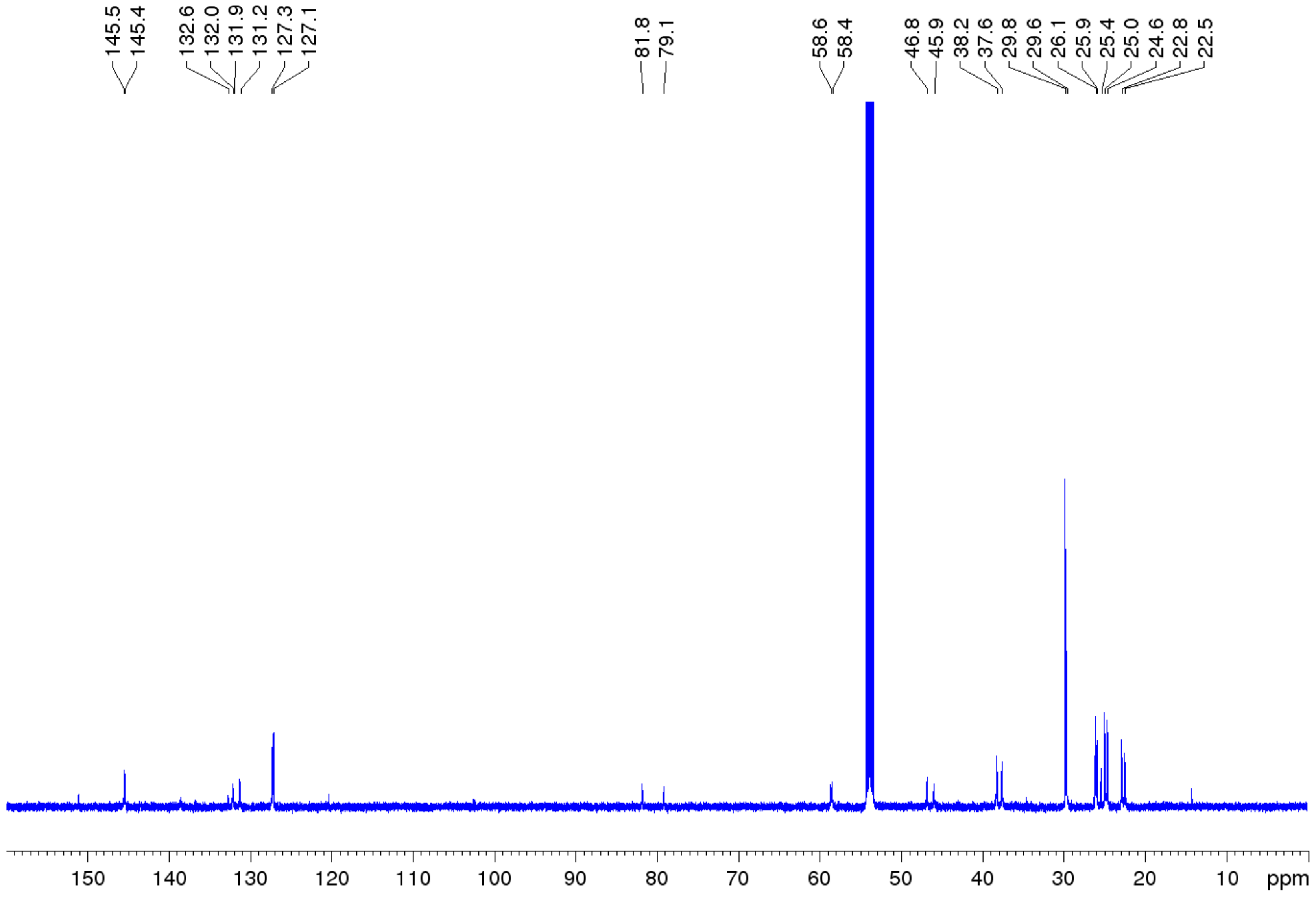

Figure S25. $^{13}C\{^1H\}$ NMR spectrum of **4-Cl** in CD_2Cl_2.

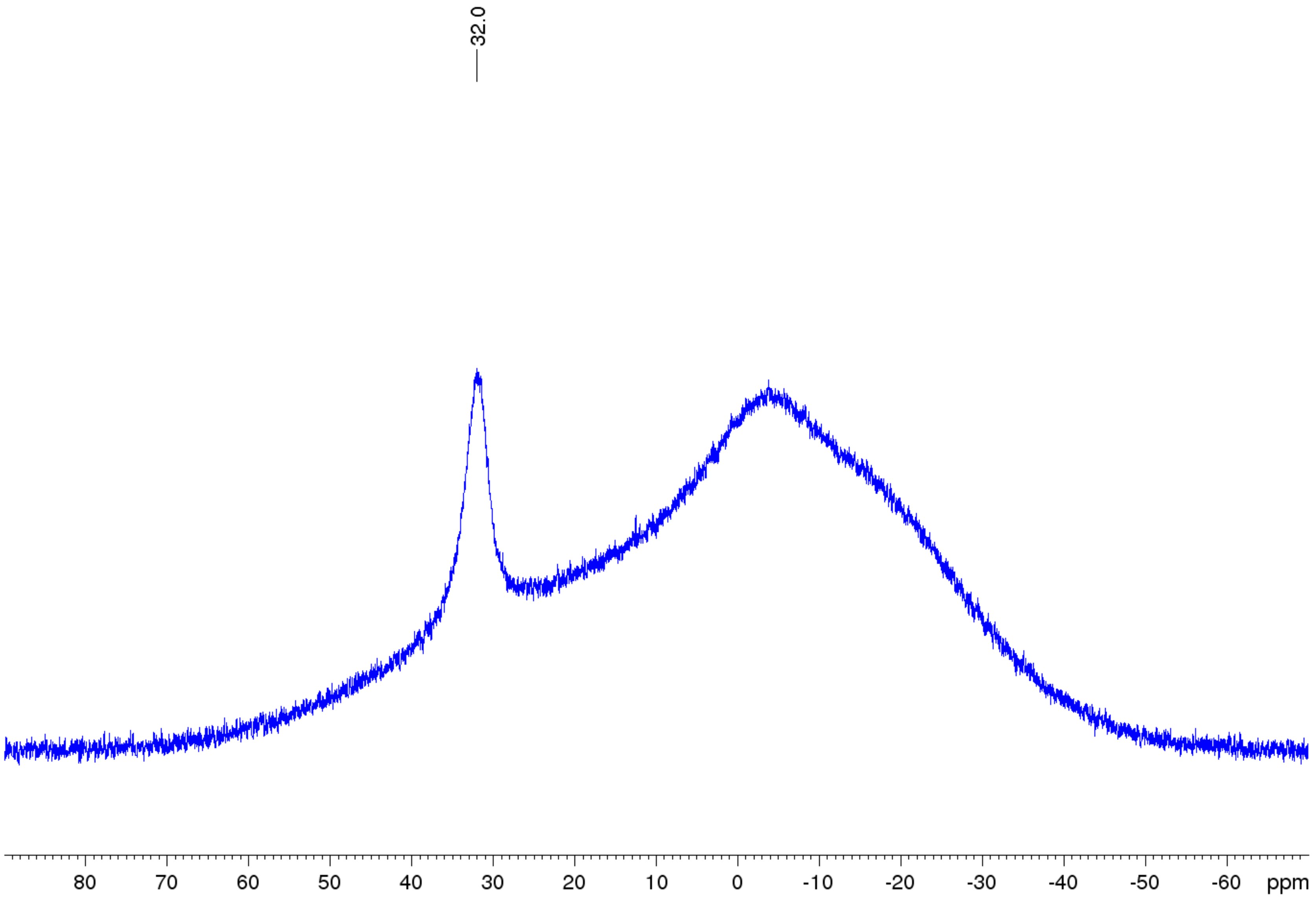

Figure S26. ^{11}B NMR spectrum of **4-Cl** in CD_2Cl_2.

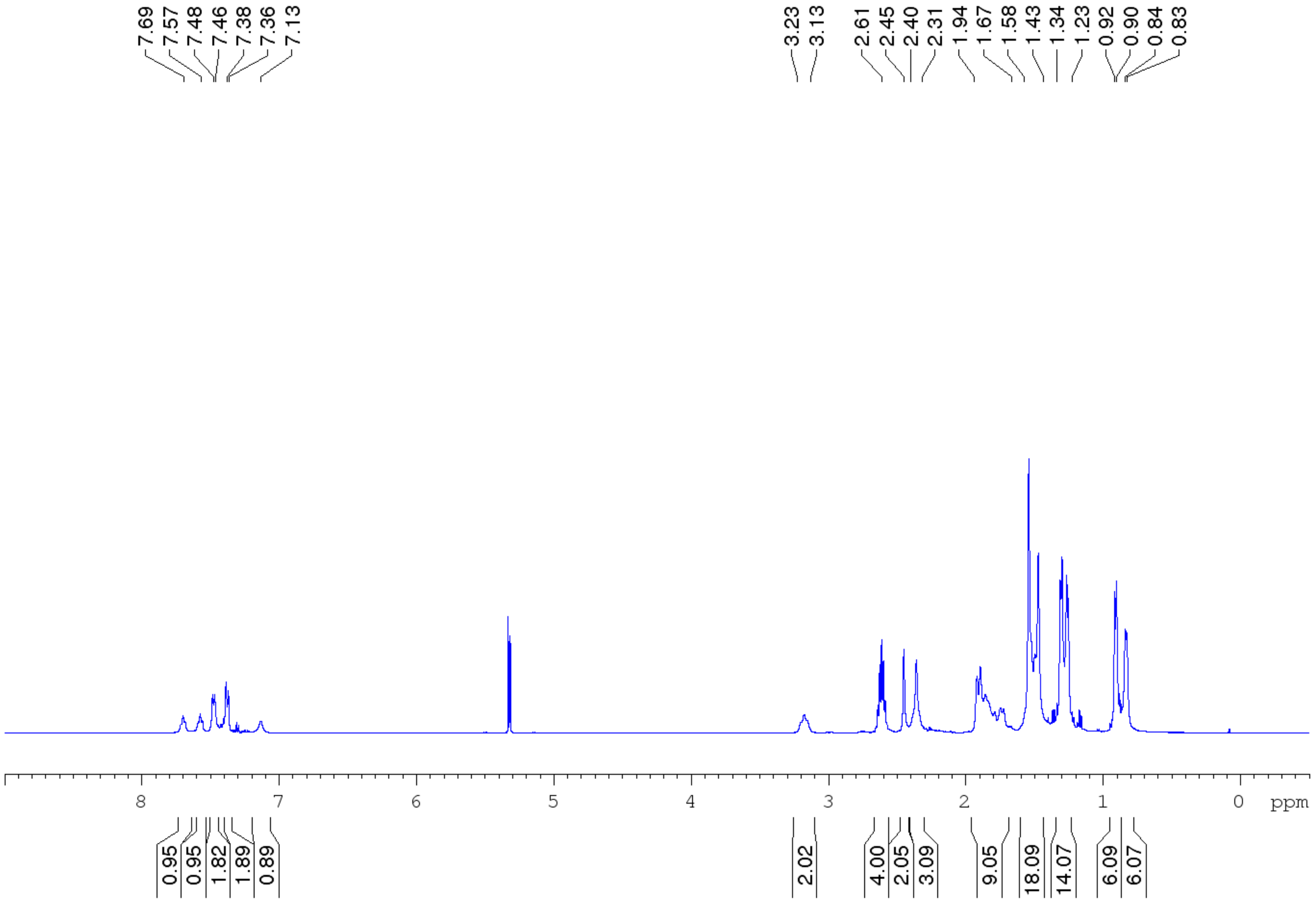

Figure S27. ^{1}H NMR spectrum of **4-OTf** in CD_2Cl_2.

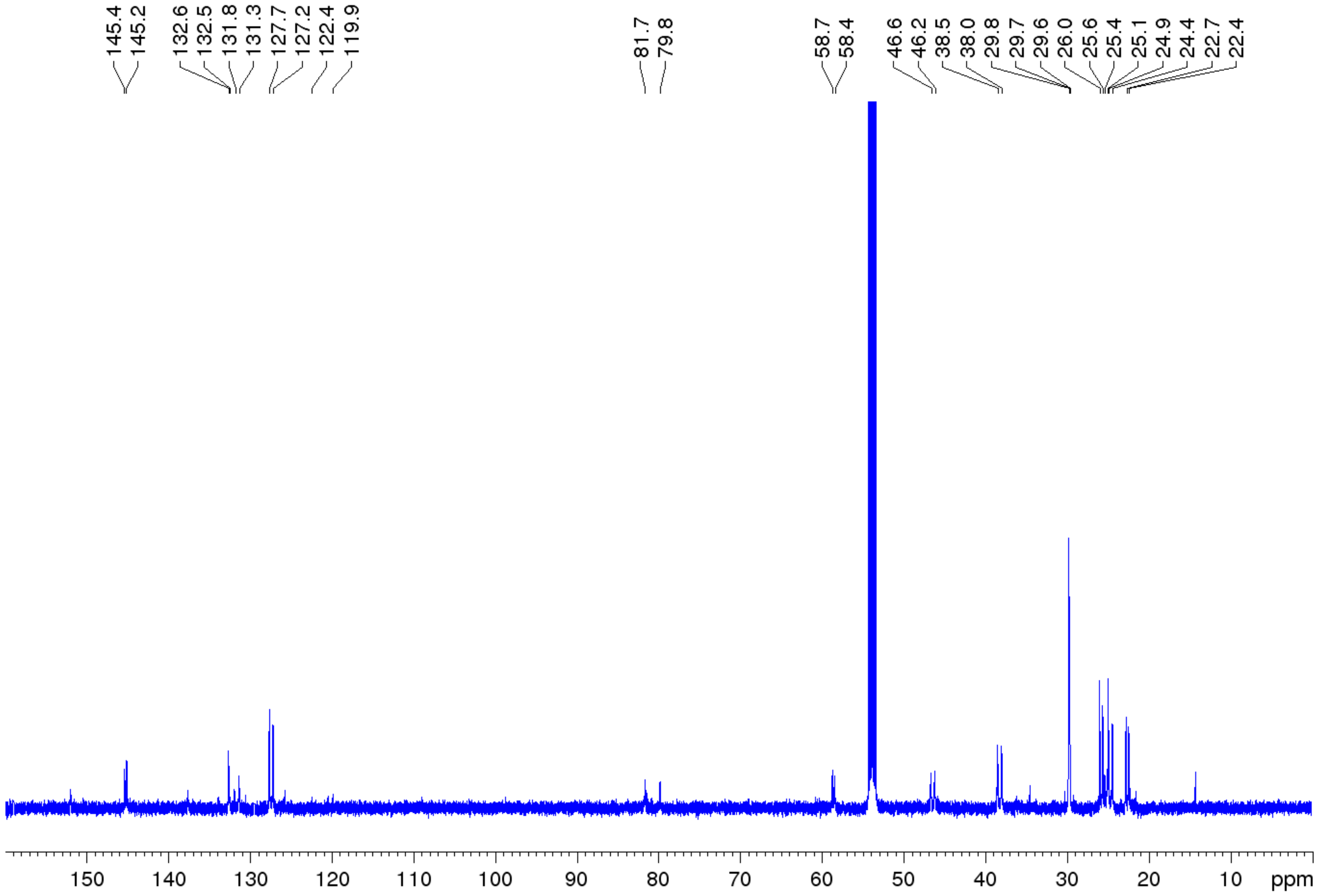

Figure S28. $^{13}C\{^1H\}$ NMR spectrum of **4-OTf** in CD_2Cl_2.

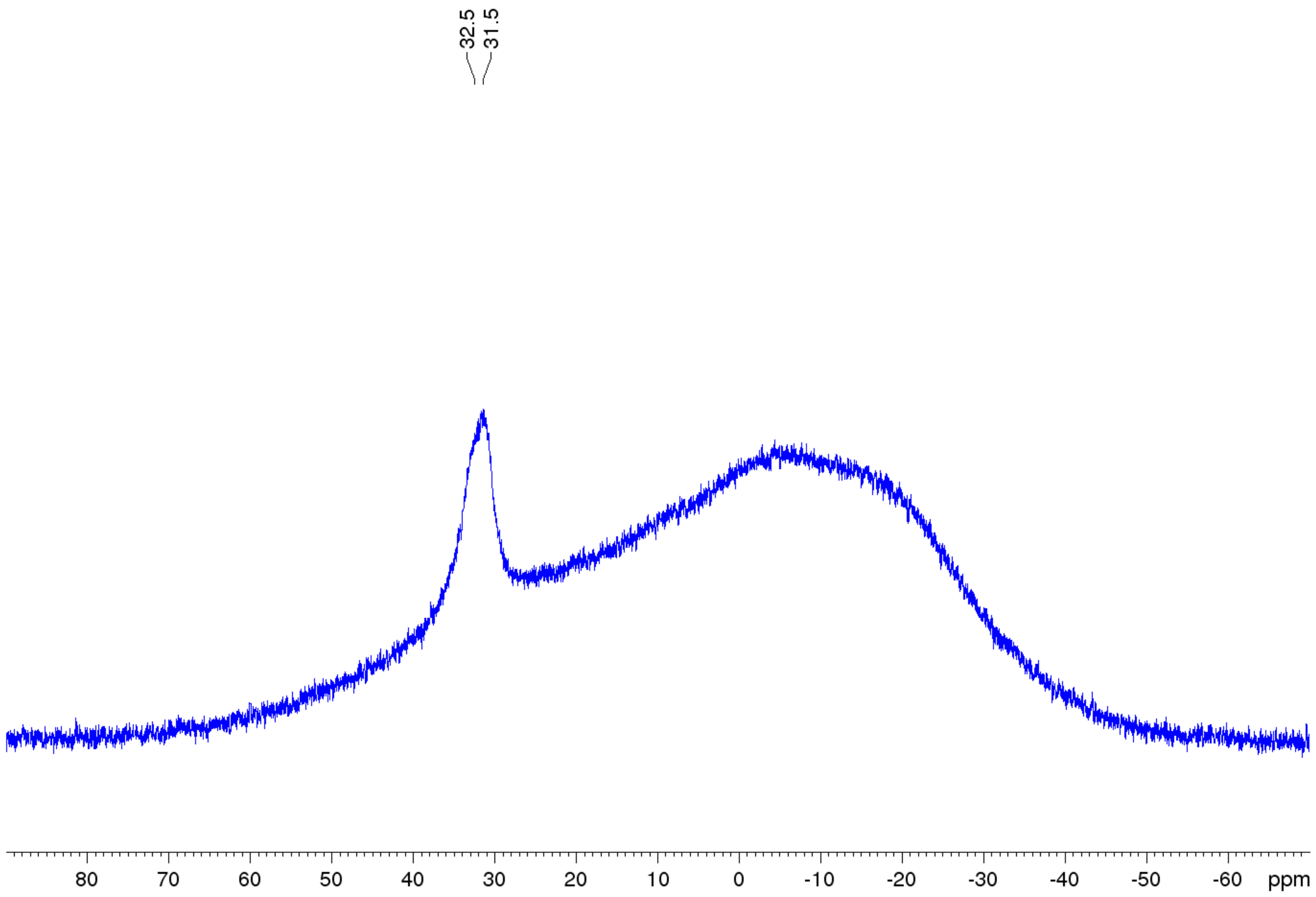

Figure S29. ^{11}B NMR spectrum of **4-OTf** in CD_2Cl_2.

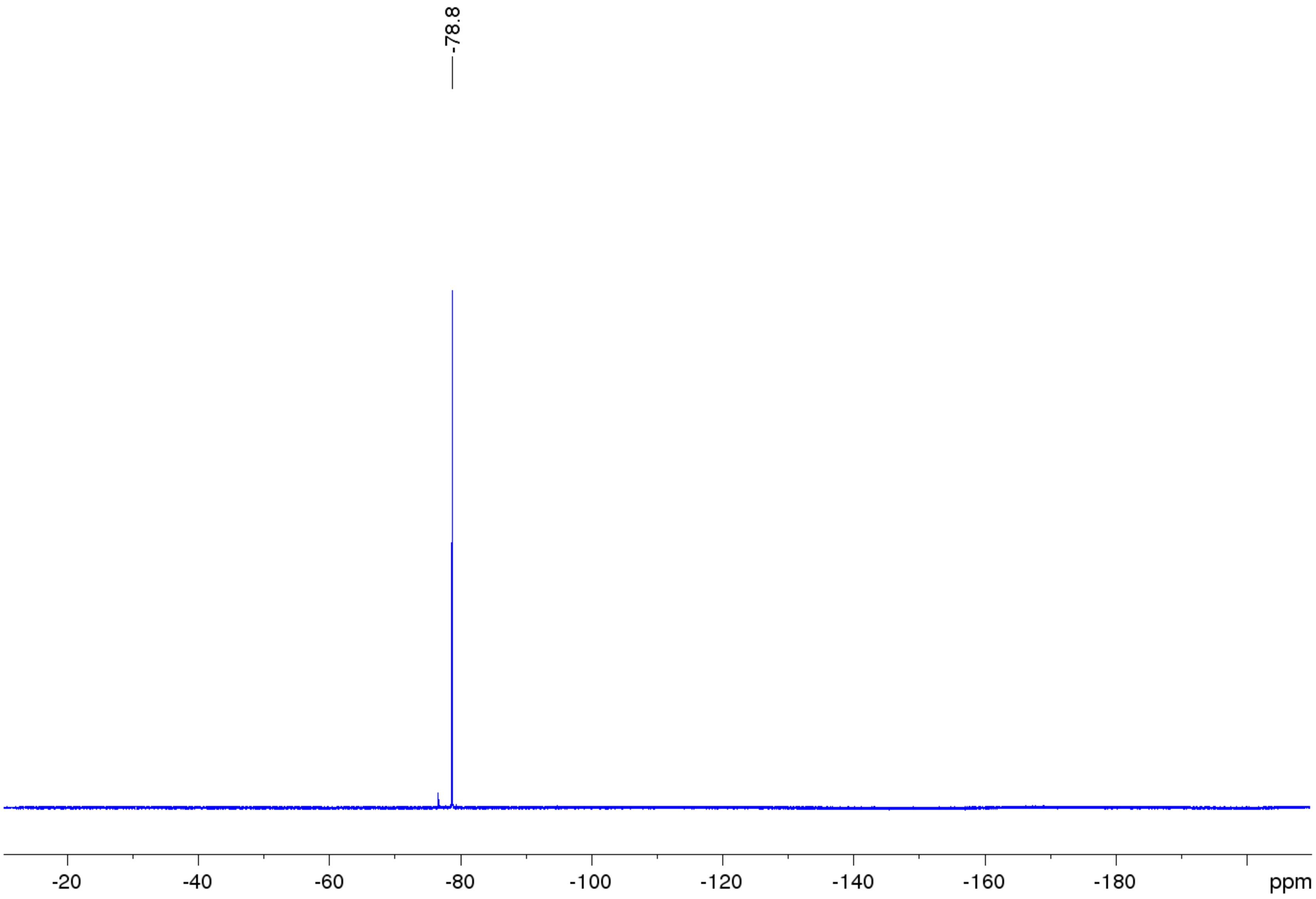

Figure S30. ^{19}F NMR spectrum of **4-OTf** in CD_2Cl_2

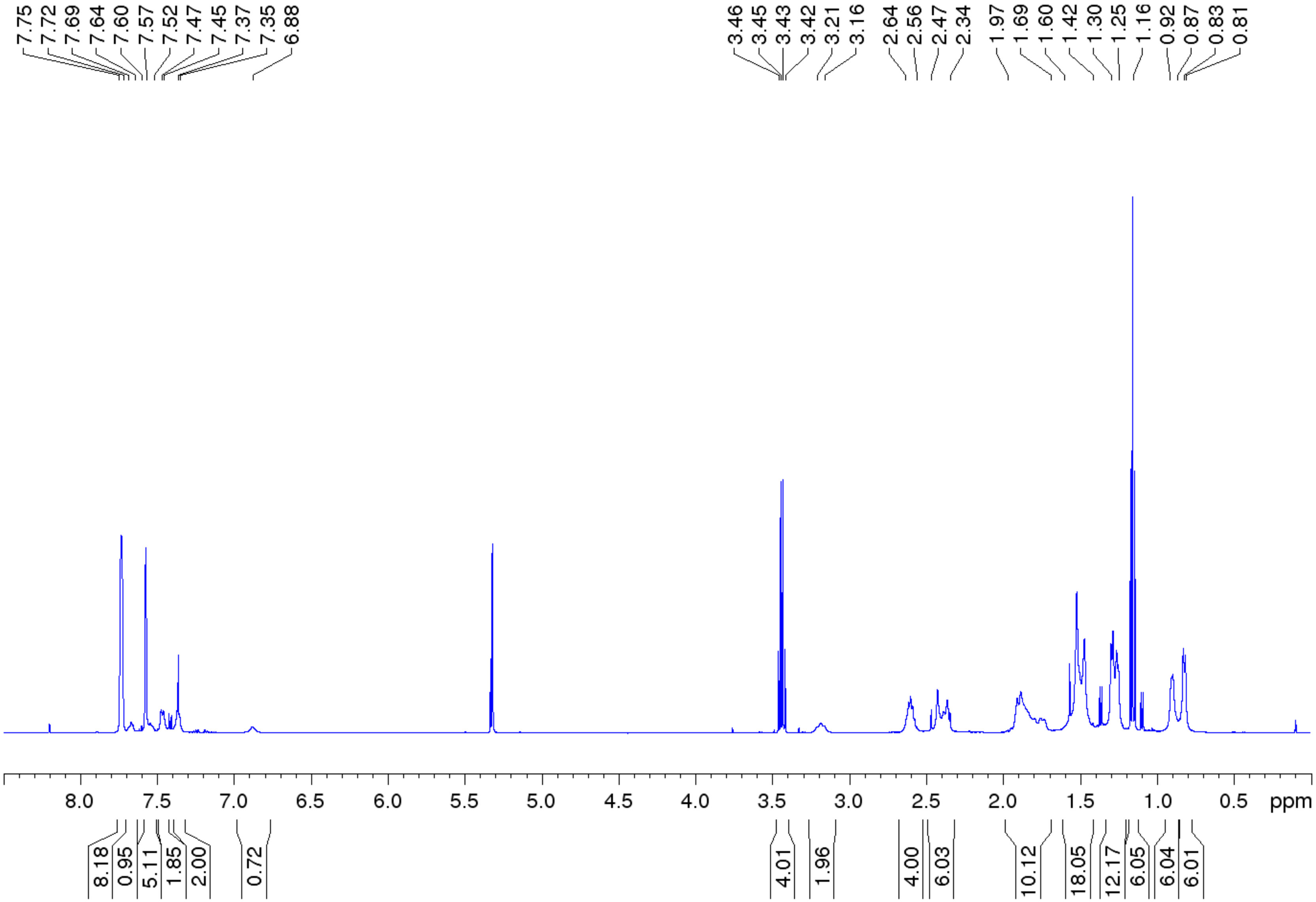

Figure S31. ^{1}H NMR spectrum of **4-BAr$^{F}_{4}$·Et$_2$O** in CD_2Cl_2.

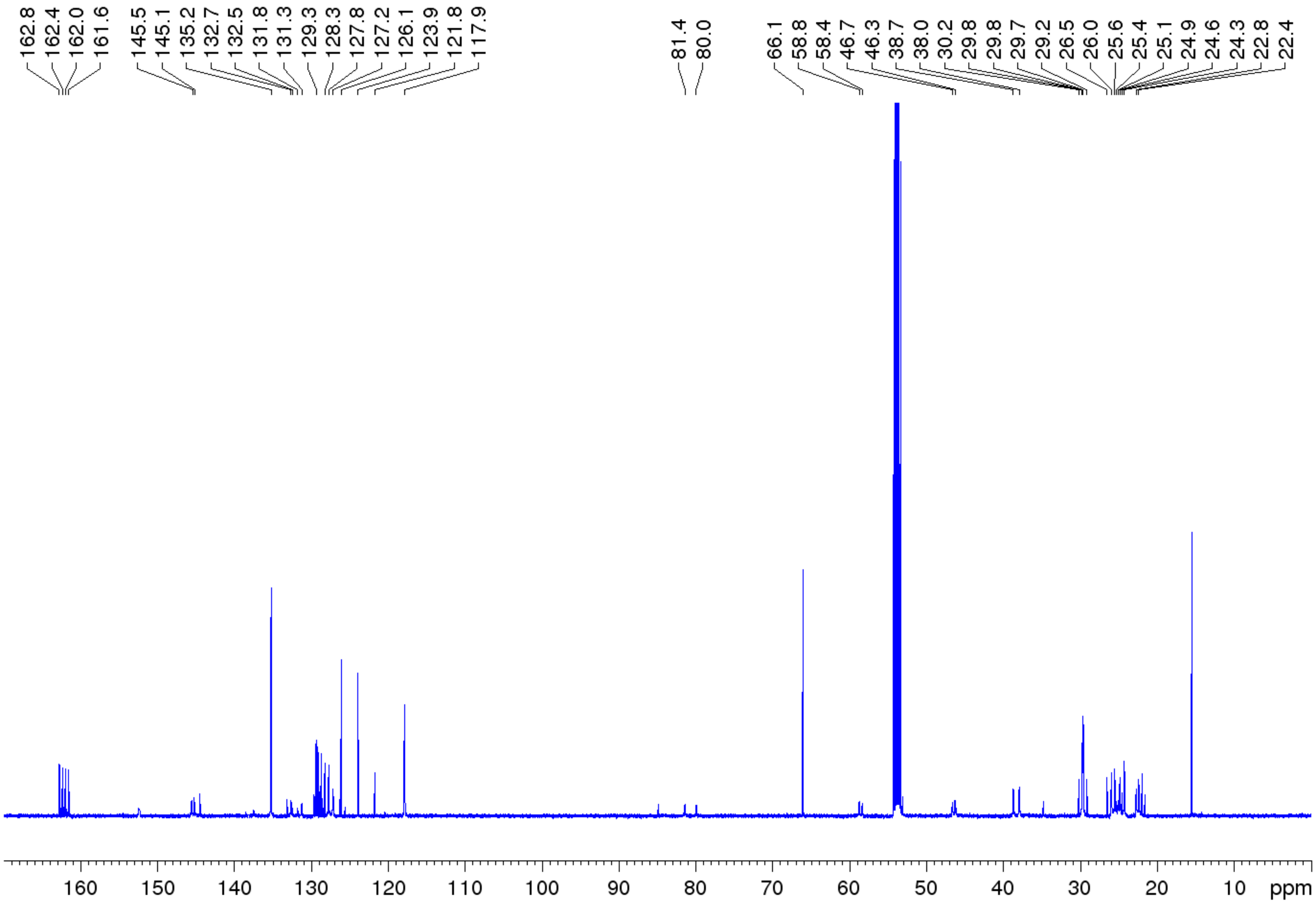

Figure S32. $^{13}C\{^{1}H\}$ NMR spectrum of **4-BAr$^{F}_{4}$·Et$_2$O** in CD_2Cl_2.

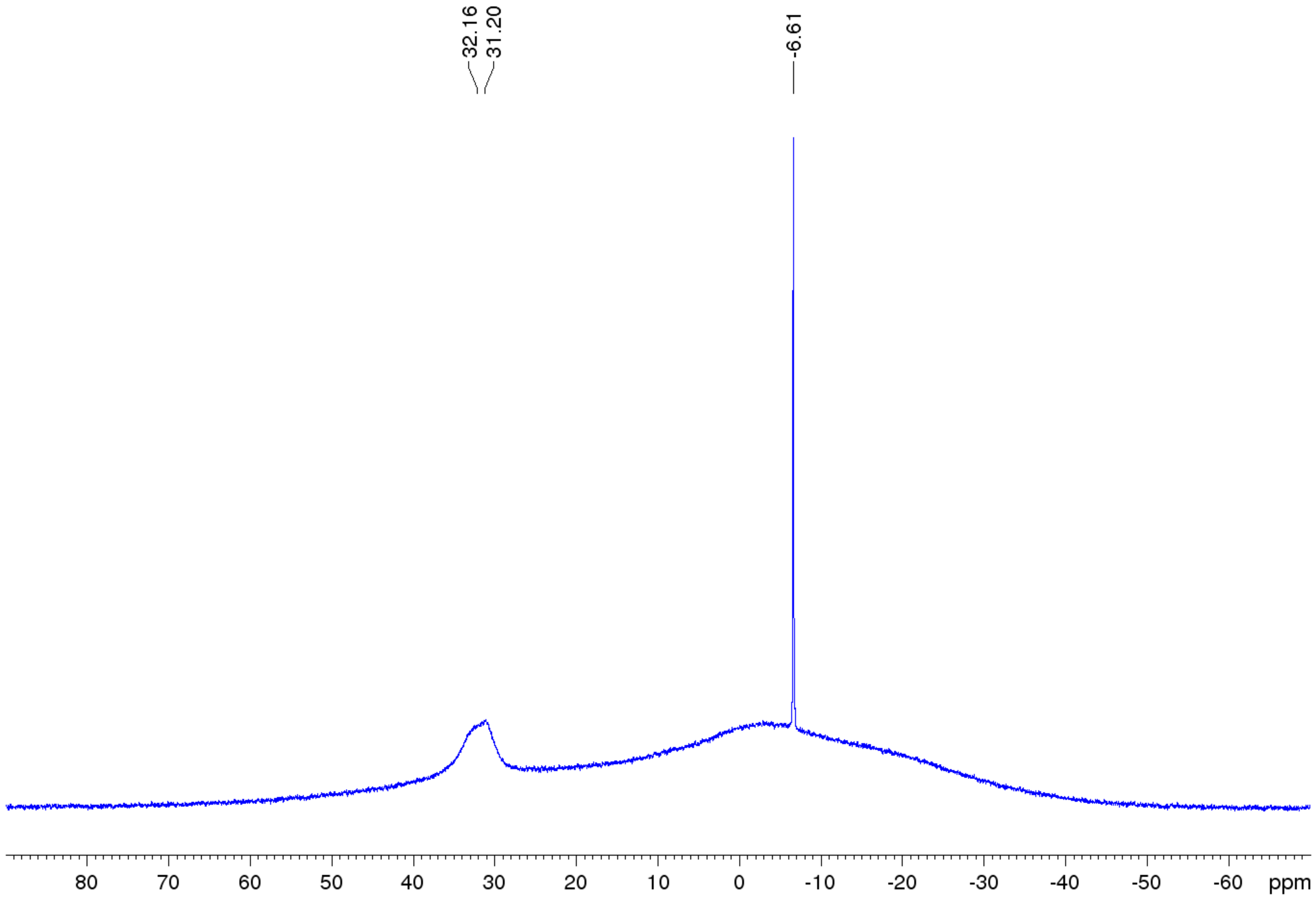

Figure S33. ^{11}B NMR spectrum of **4-BAr$^{F}_{4}$·Et$_2$O** in CD_2Cl_2.

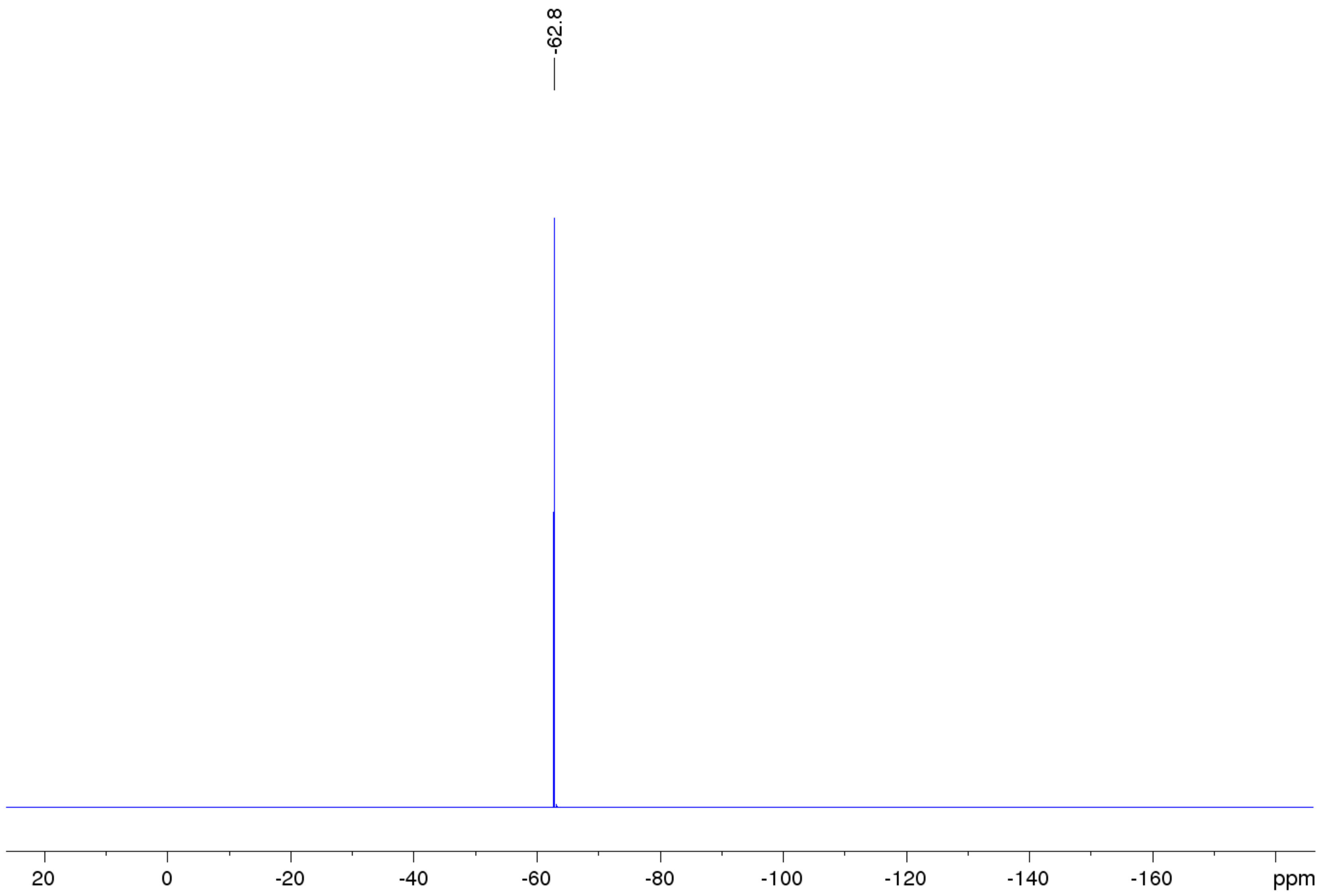

Figure S34. ^{19}F NMR spectrum of **4-BAr^{F}$_4$·Et$_2$O** in CD_2Cl_2.

Stack-plot of ^{1}H NMR spectra of 1', 4-Cl and 3-Cl

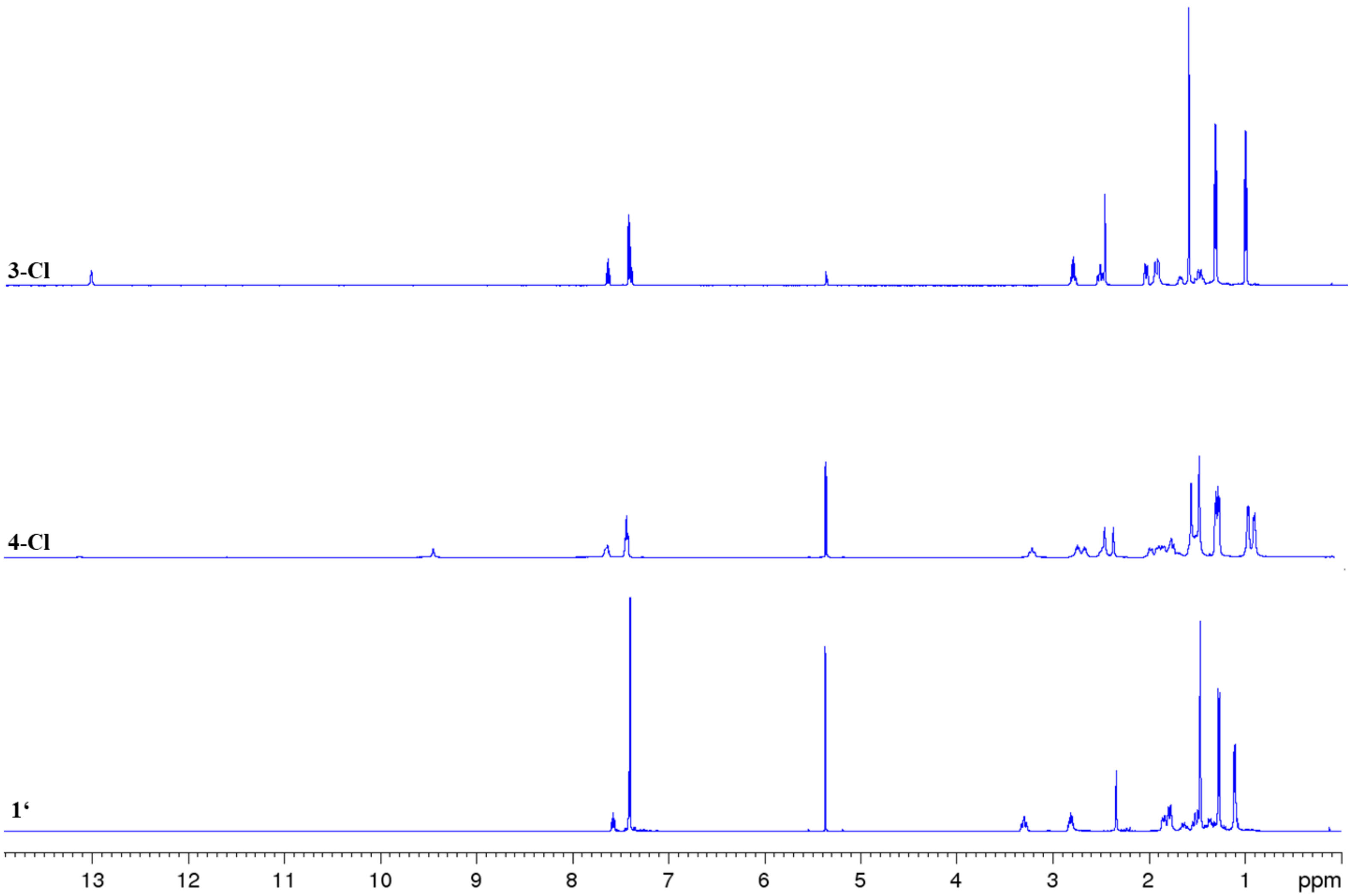

Figure S35. Stack-plot of ^{1}H NMR spectra of **1'**, **4-Cl** and **3-Cl** in CD_2Cl_2.

Stack-plot of ^{1}H NMR spectra of 1', 4-OTf and 3-OTf

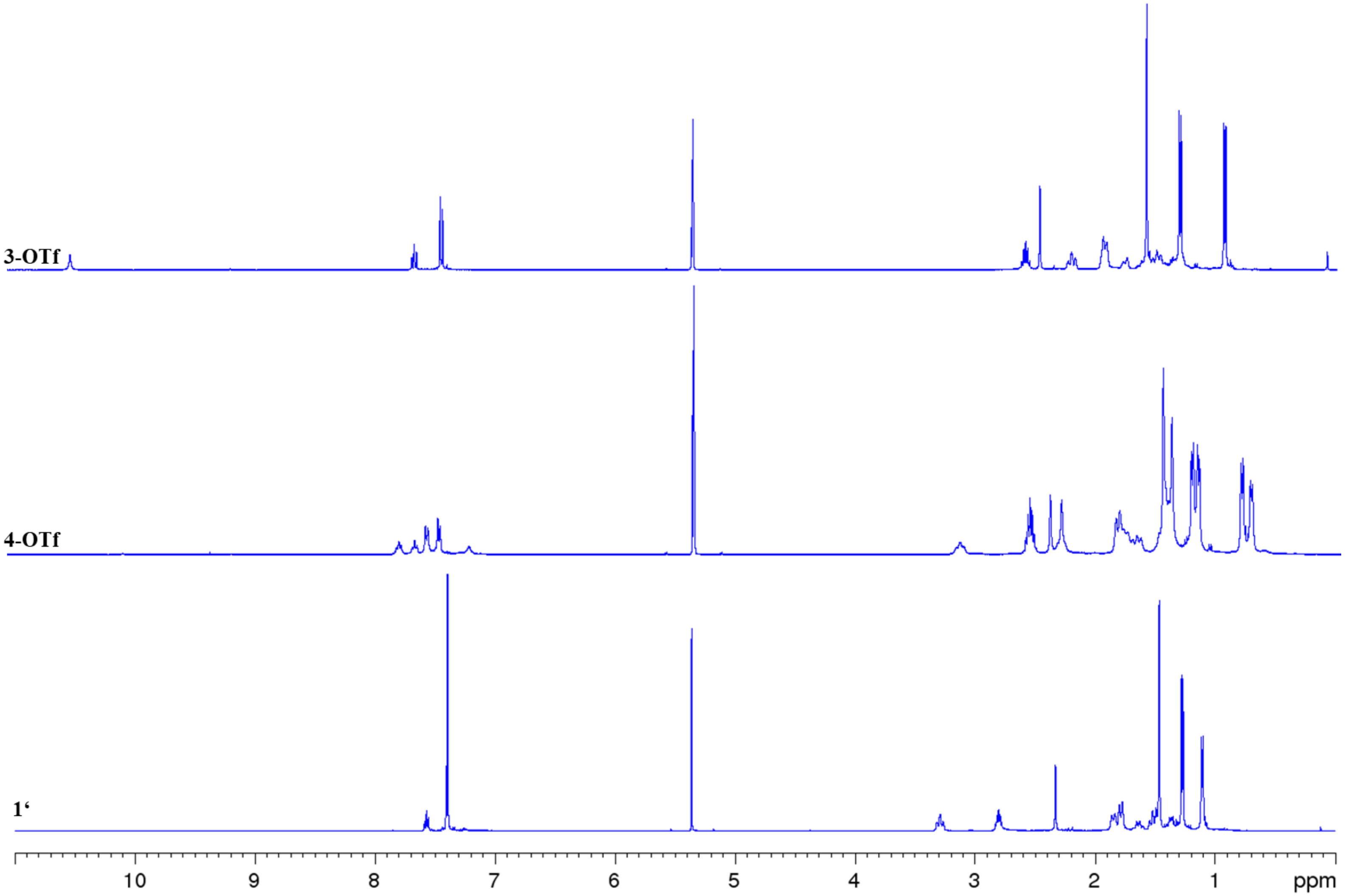

Figure S36. Stack-plot of ^{1}H NMR spectra of **1'**, **4-OTf** and **3-OTf** in CD_2Cl_2.

Stack-plot of ^{1}H NMR spectra of 1', 4-BAr$^F_4$·Et$_2$O and 3-BAr$^F_4$·Et$_2$O

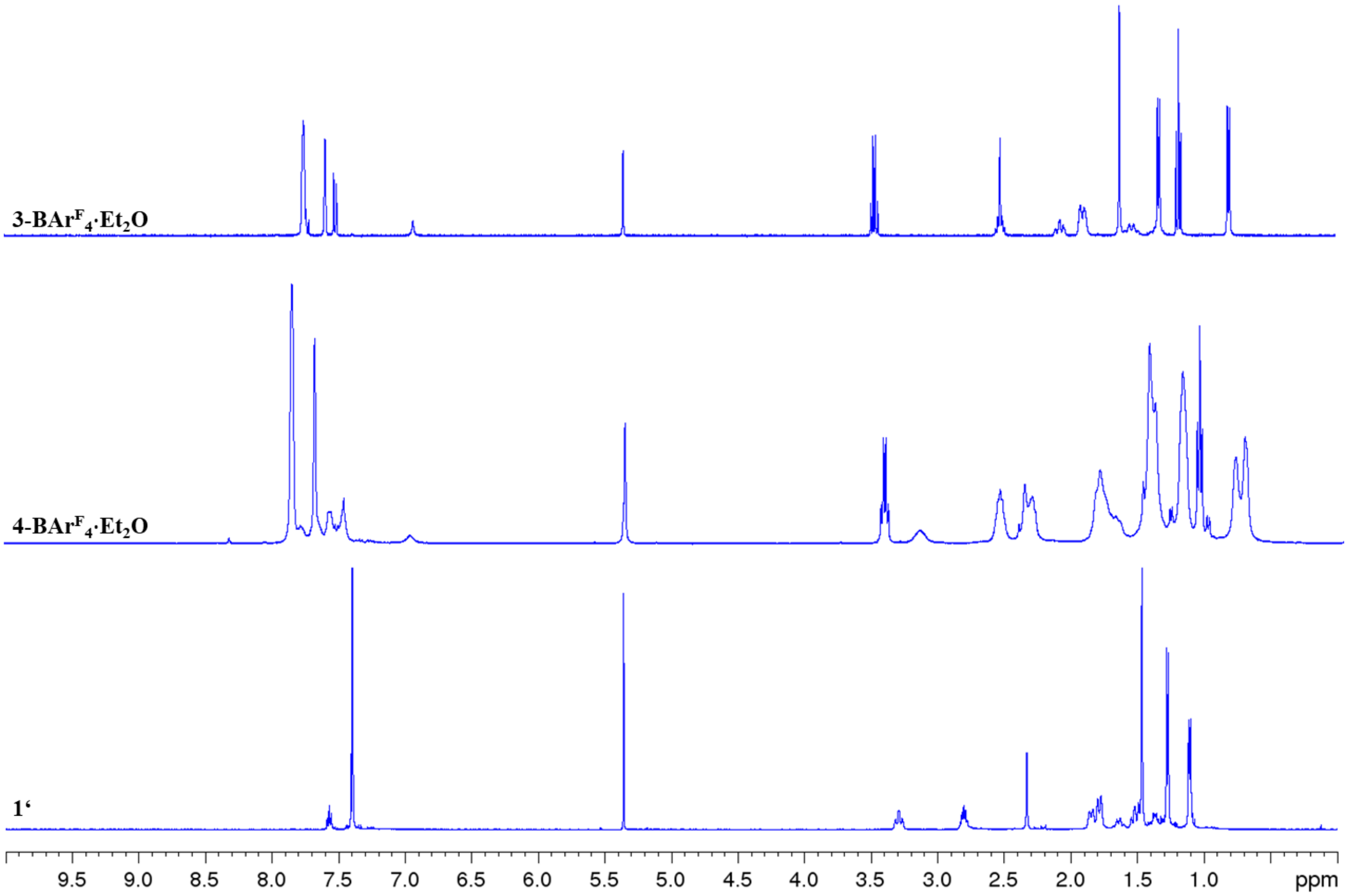

Figure S37. Stack-plot of ^{1}H NMR spectra of **1'**, **4-BAr$^F_4$·Et$_2$O** and **3-BAr$^F_4$·Et$_2$O** in CD_2Cl_2.

Stack-plot of ^{1}H NMR spectra of 3-Cl, 3-OTf, 3-OTf·HOTf and 3-BAr$^{F}_{4}$·Et$_2$O

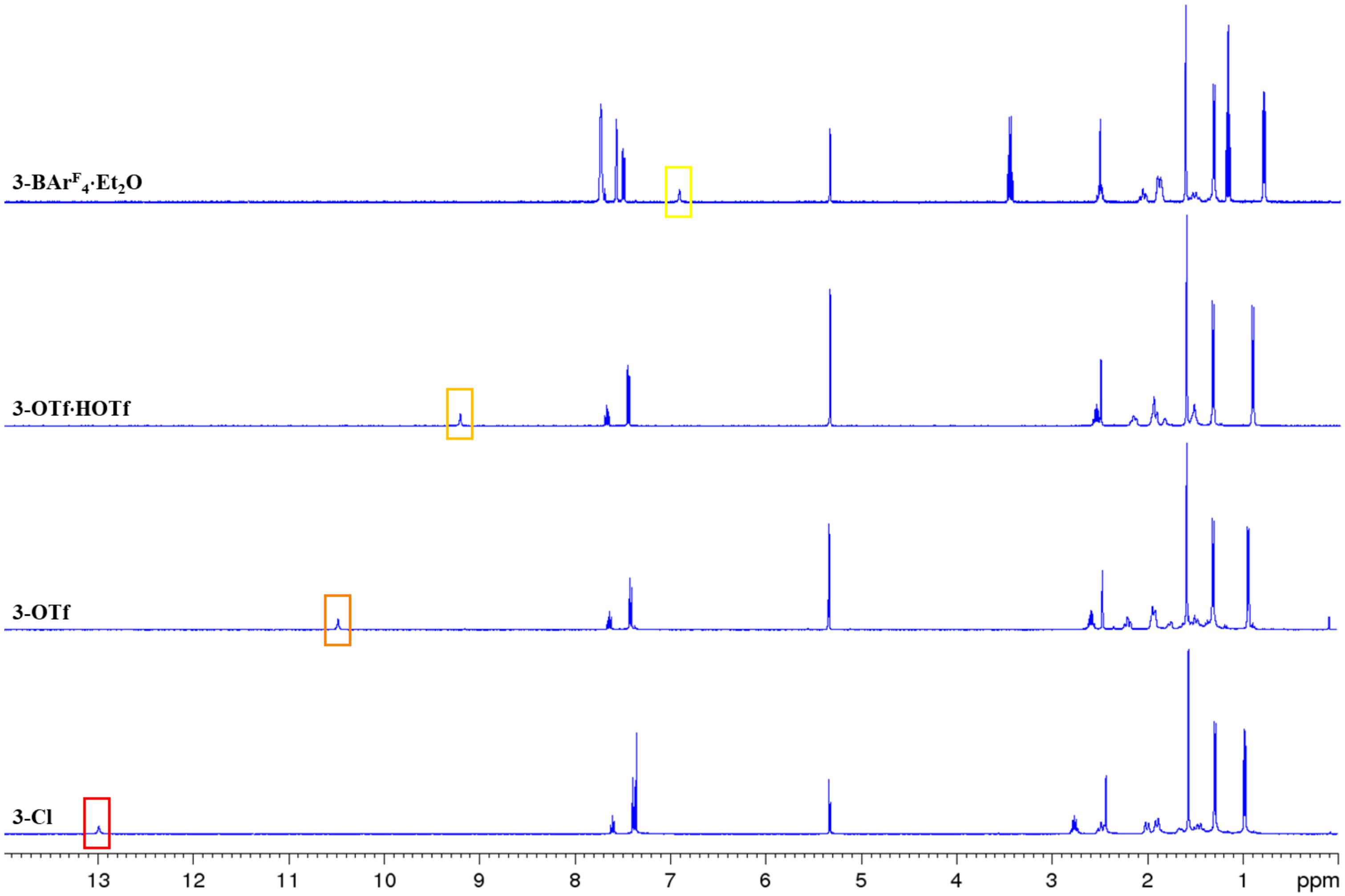

Figure S38. Stack-plot of ^{1}H NMR spectra of **3-Cl**, **3-OTf**, **3-OTf·HOTf** and **3-BAr$^{F}_{4}$·Et$_2$O** in CD_2Cl_2. The boxes mark the N–H resonance and their contours represent the color of the corresponding dication.

IR spectra

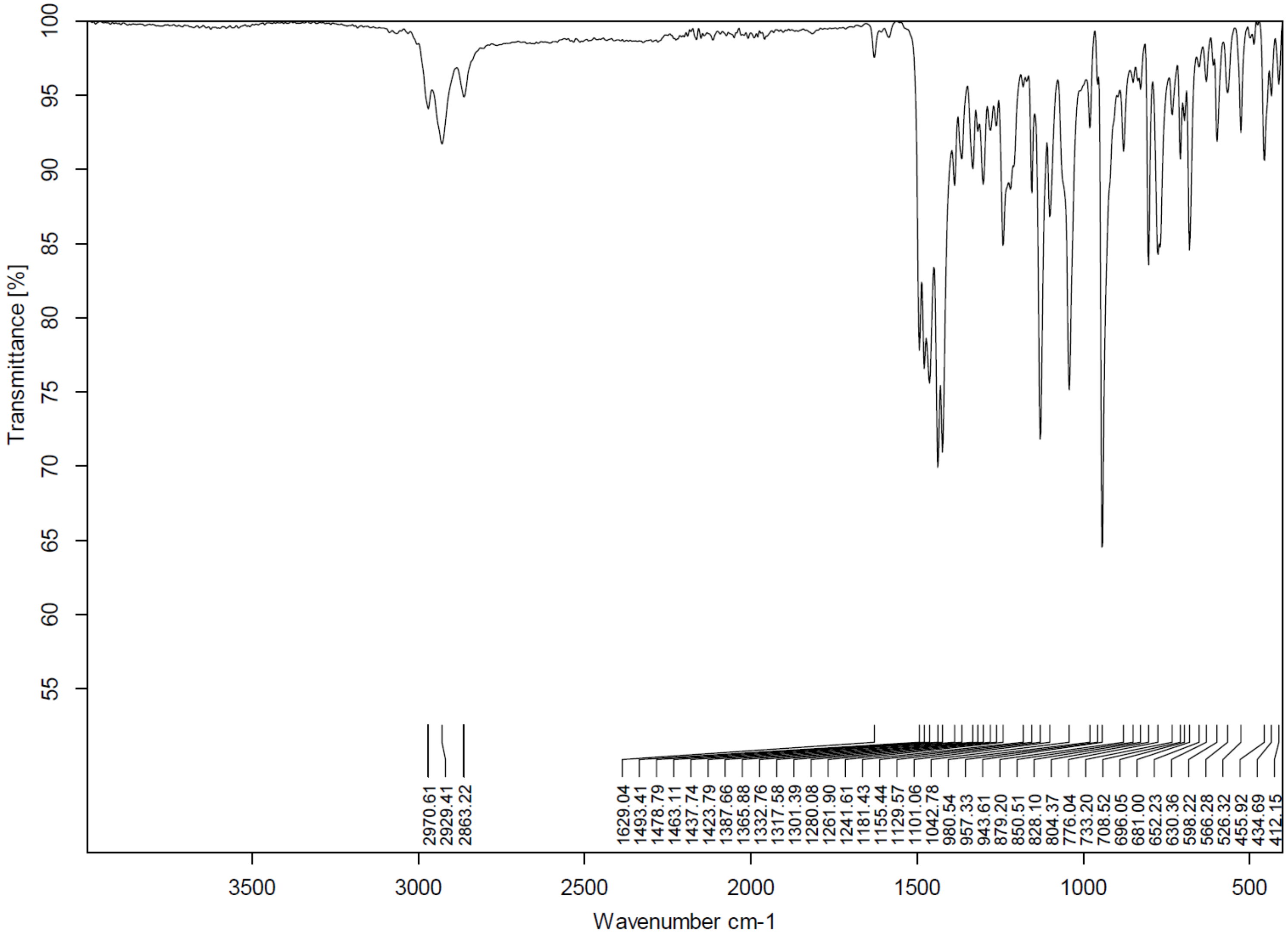

Figure S39. Solid-state IR spectrum of **2**.

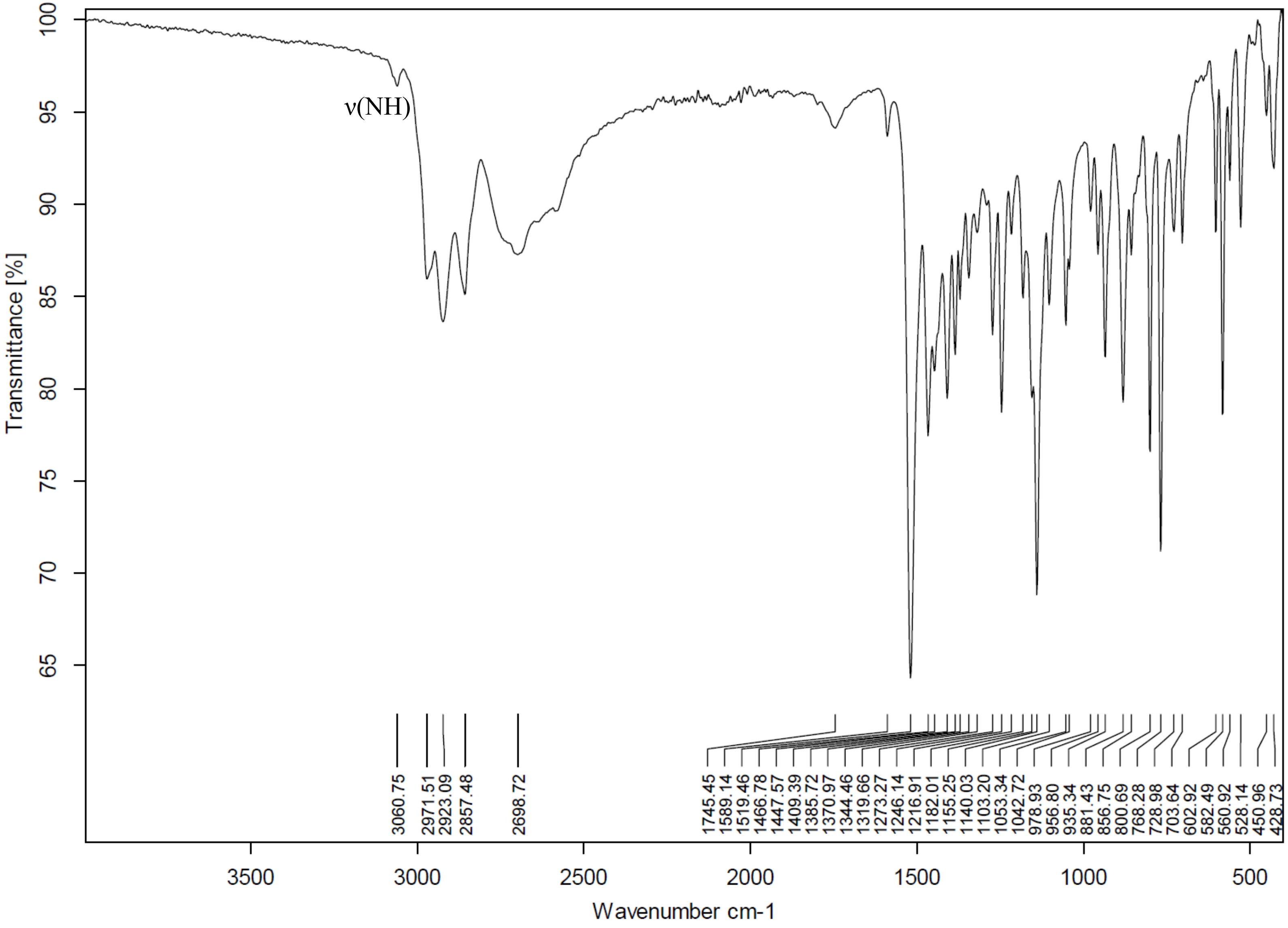

Figure S40. Solid-state IR spectrum of **3-Cl**.

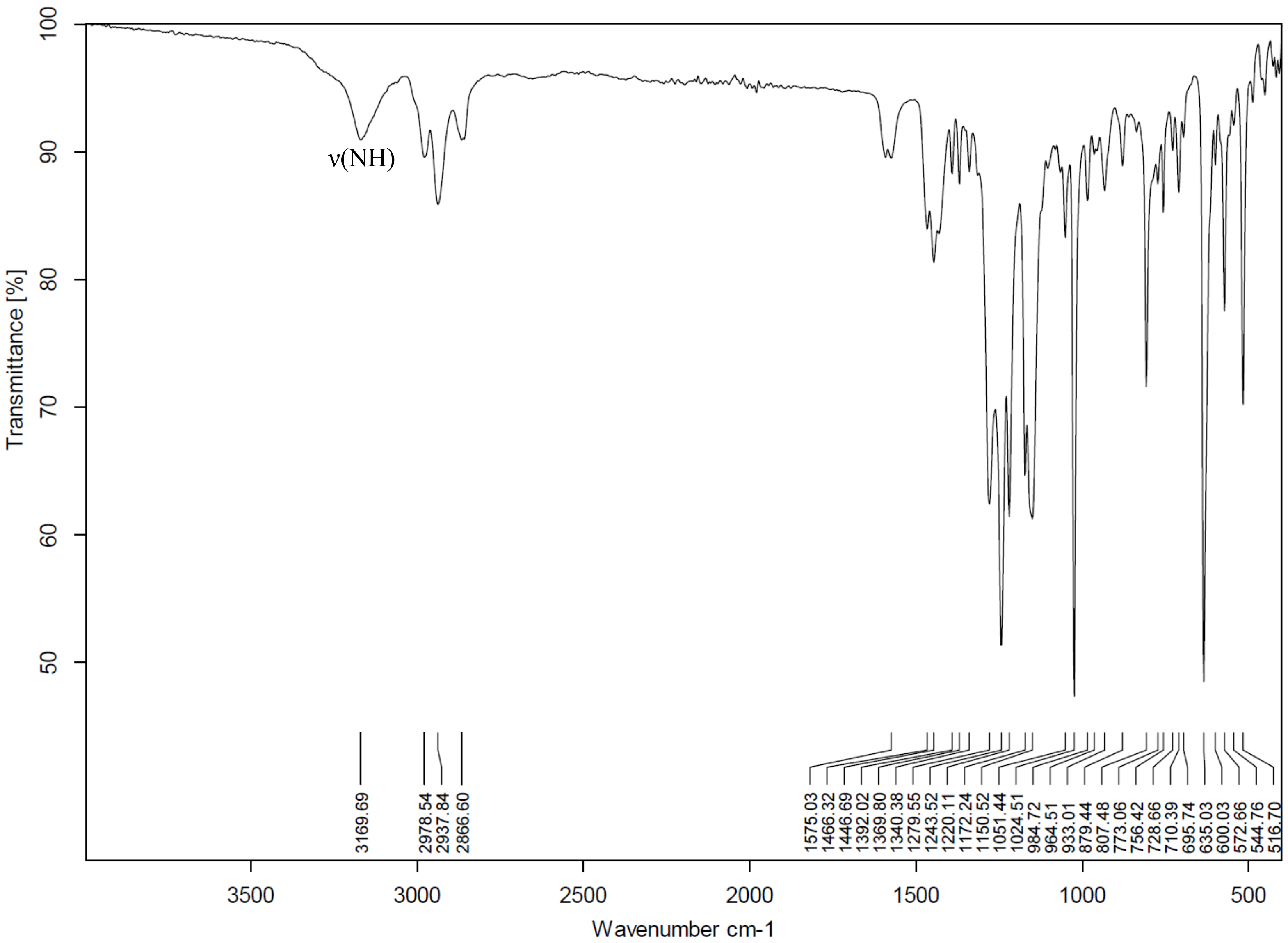

Figure S41. Solid-state IR spectrum of **3-OTf**.

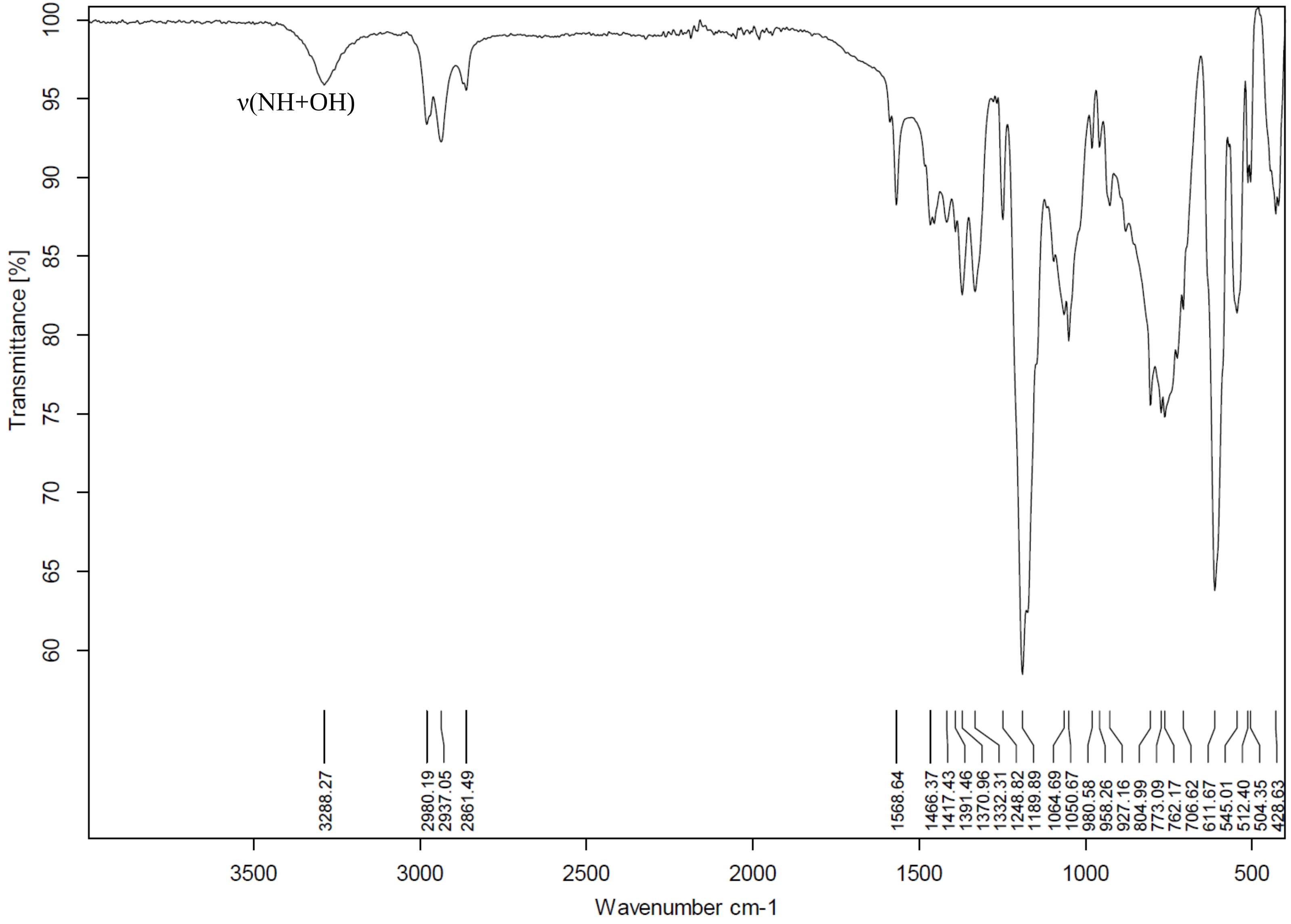

Figure S42. Solid-state IR spectrum of **3-OTf·HOTf**.

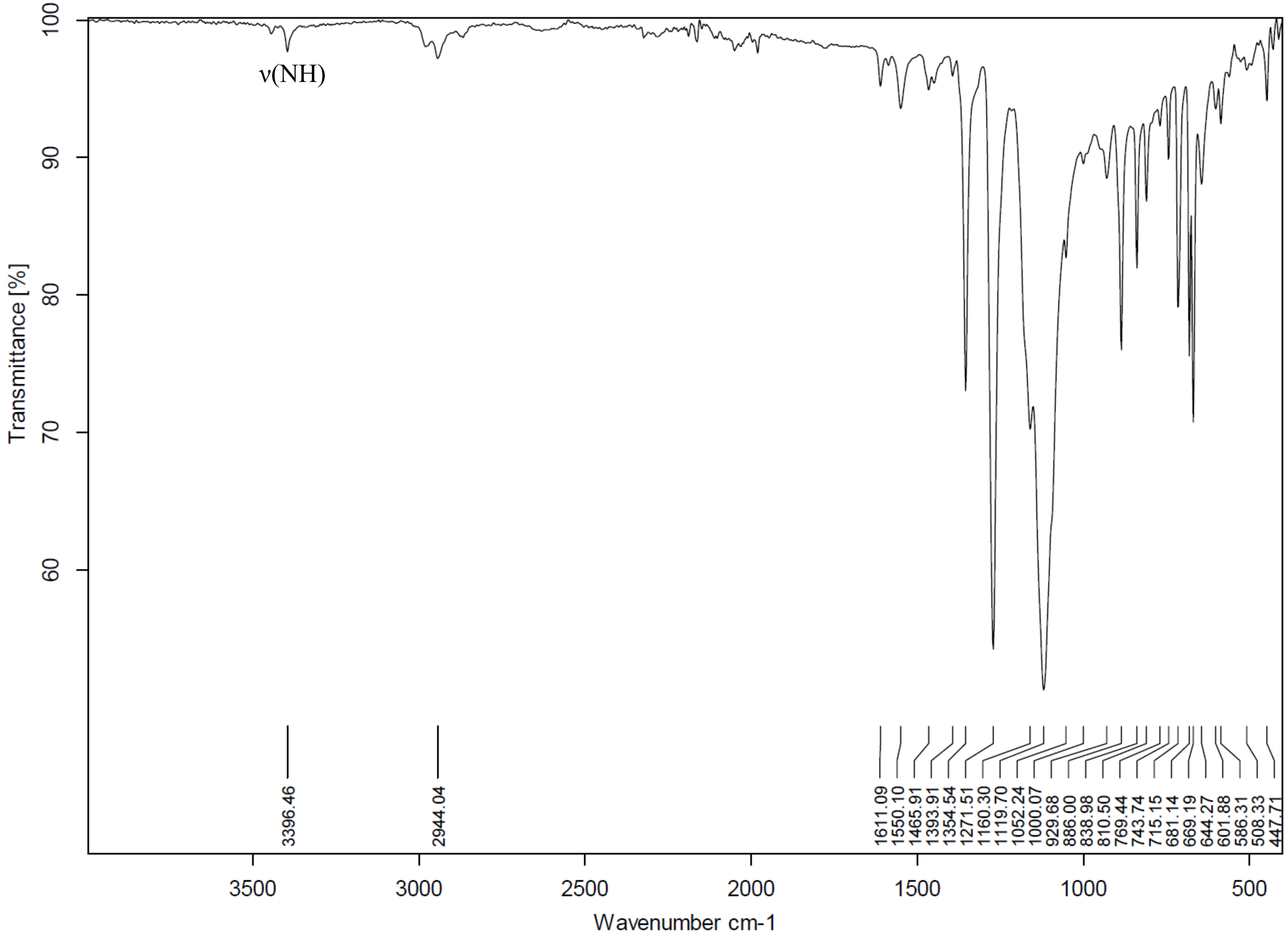

Figure S43. Solid-state IR spectrum of **3-BAr$^{F}_{4}$·Et$_2$O**.

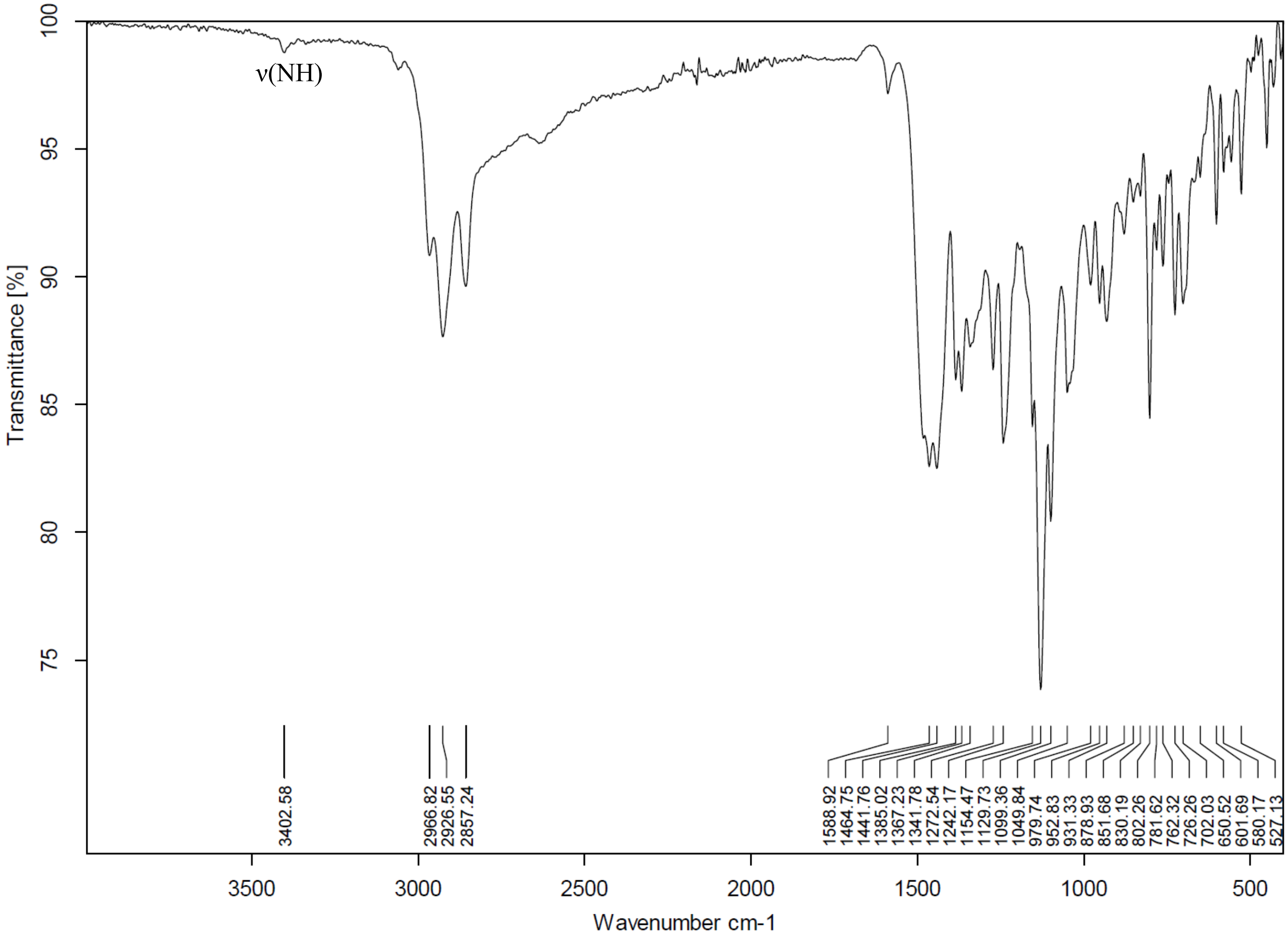

Figure S44. Solid-state IR spectrum of **4-Cl**.

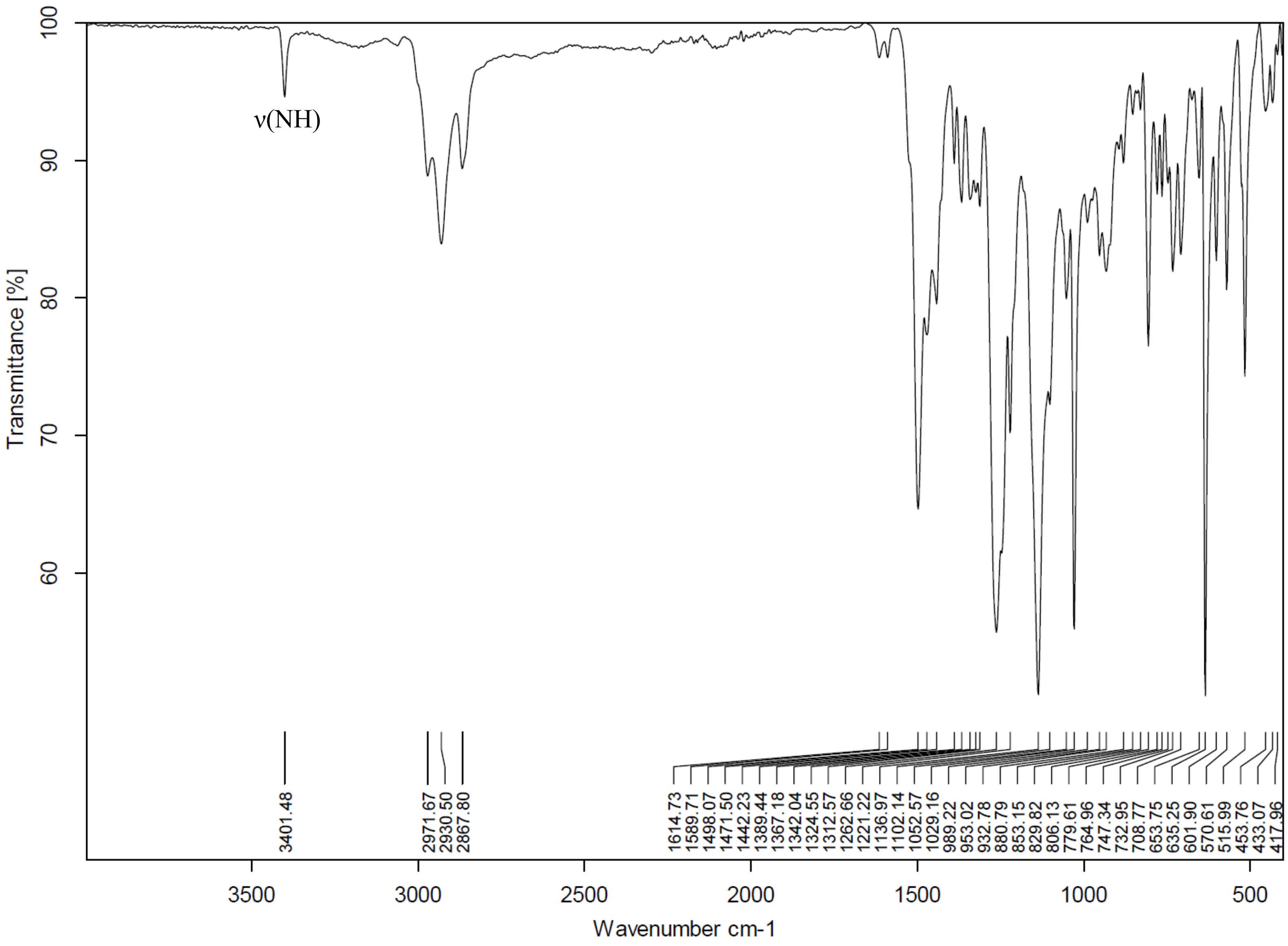

Figure S45. Solid-state IR spectrum of **4-OTf**.

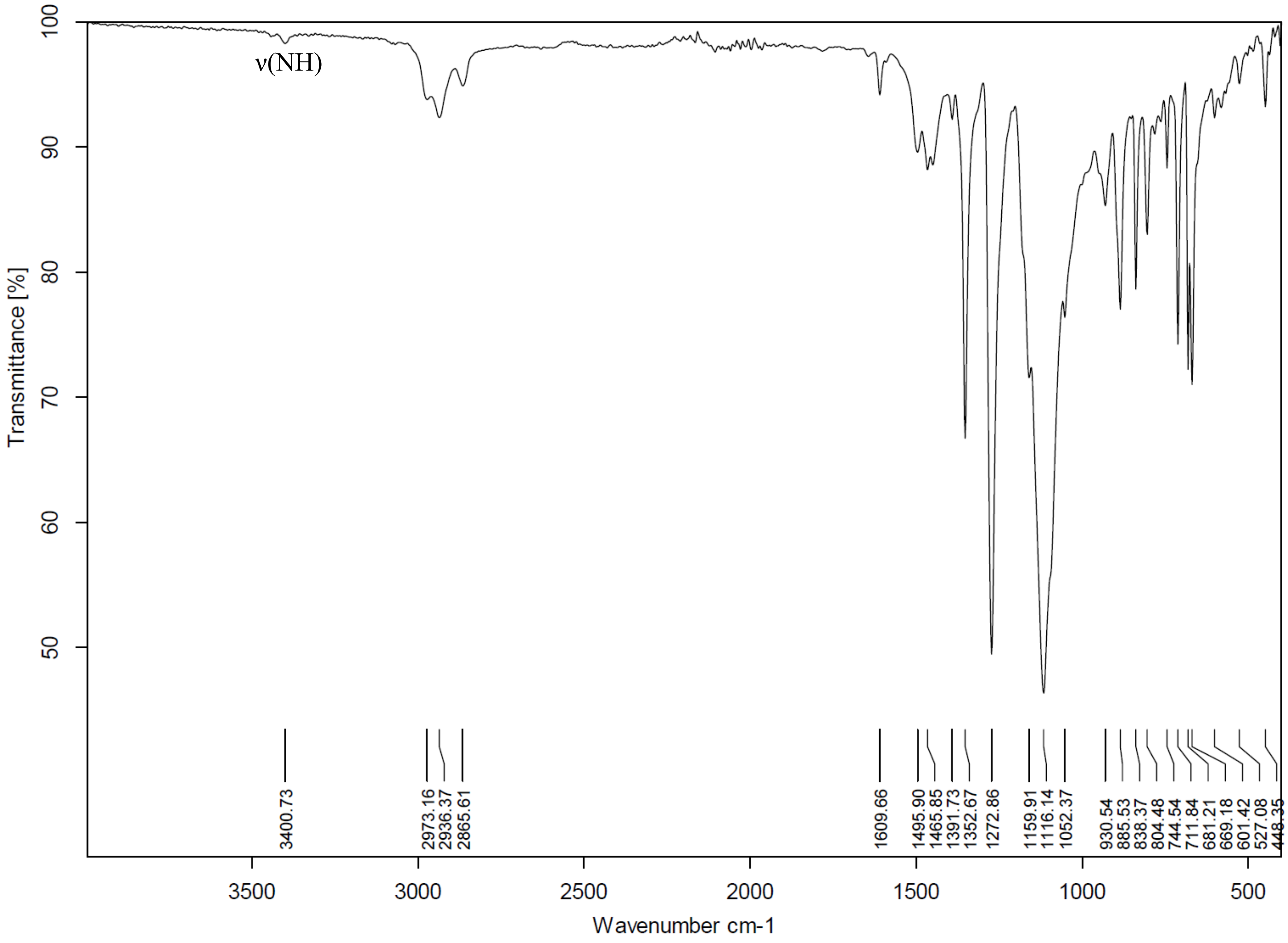

Figure S46. Solid-state IR spectrum of **4-BAr$^{F}_{4}$·Et$_2$O**.

UV-vis spectra

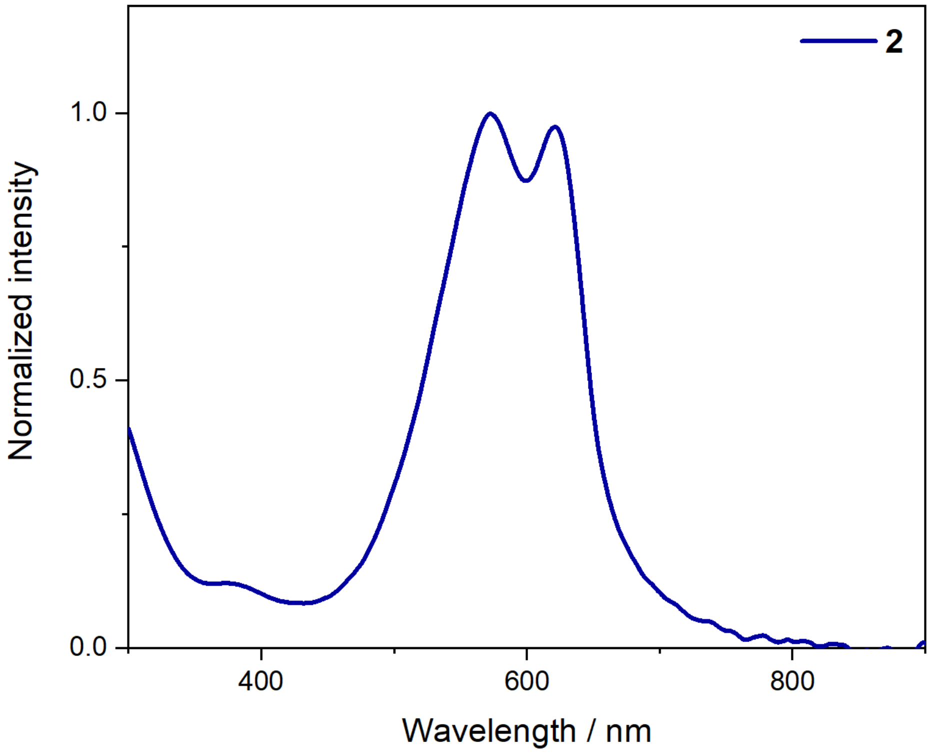

Figure S47. UV-vis absorption spectrum of **2** in CH_2Cl_2 at 25 °C.

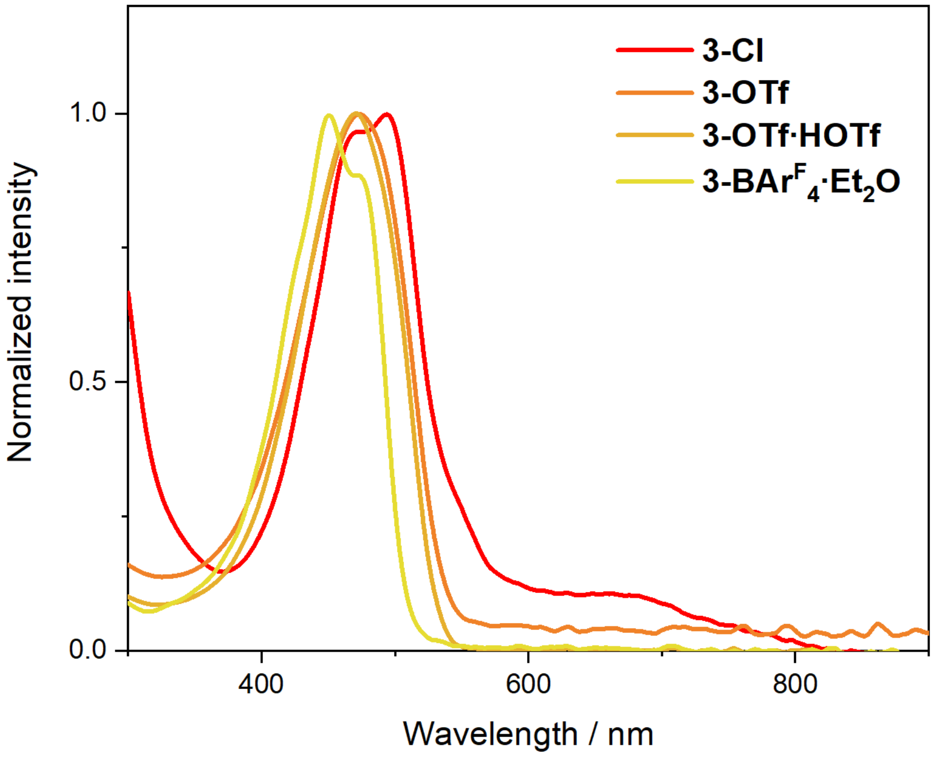

Figure S48. UV-vis absorption spectra of **3-X** with X = Cl (red), OTf (dark orange), OTf·HOTf (light orange), and $BAr^F{}_4 \cdot Et_2O$ (yellow) in CH_2Cl_2 at 25 °C.

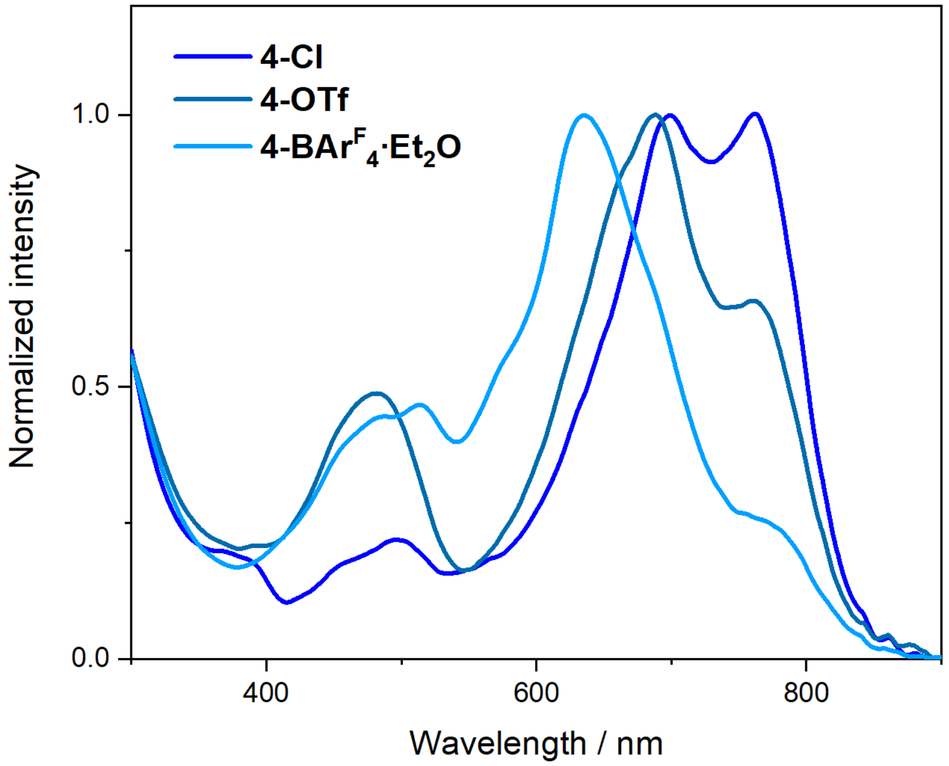

Figure S49. UV-vis absorption spectra of **4-X** with X = Cl (dark blue), OTf (petrol blue), and $BAr^{F}_{4}\cdot Et_2O$ (light blue) in CH_2Cl_2 at 25 °C.

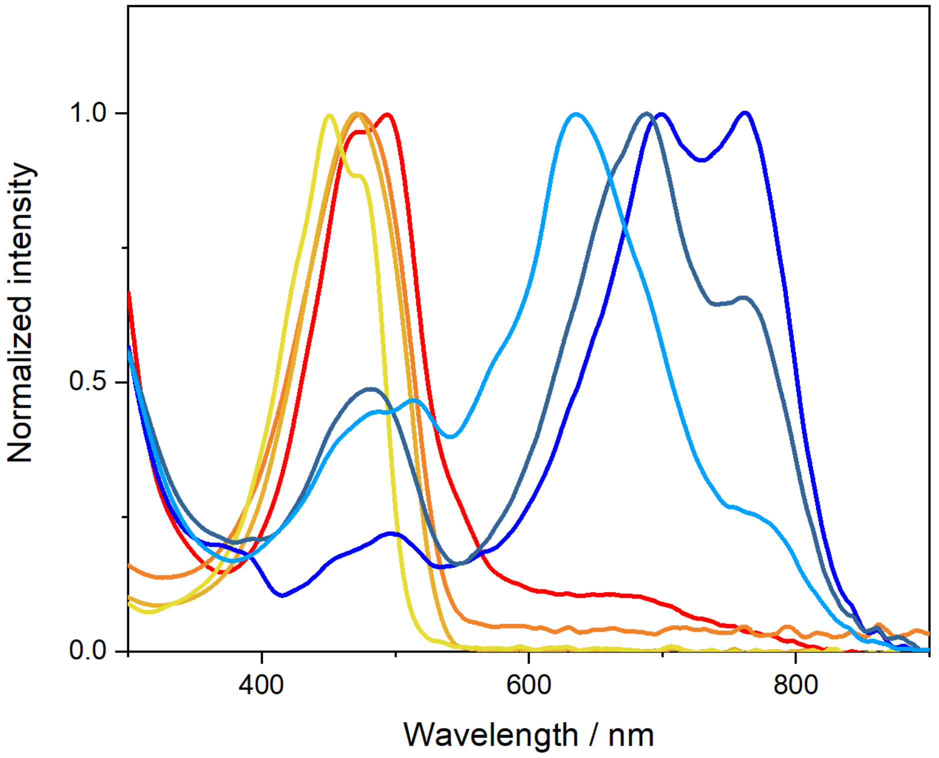

Figure S50. Overlap of UV-vis absorption spectra of **3-X** and **4-X** in CH_2Cl_2 at 25 °C. See Figures S48 and S49 for color code.

X-ray crystallographic data

The crystal data of **3-OTf·HOTf** and **3-BAr^F$_4$·Et$_2$O** were collected on a Bruker X8-APEX II diffractometer with a CCD area detector and multi-layer mirror monochromated Mo$_{K\alpha}$ radiation. The crystal data **2** was collected on a Bruker D8 Quest diffractometer with a CMOS area detector and multi-layer mirror monochromated Mo$_{K\alpha}$ radiation. The crystal data of **2**, **3-OTf** and **3-BAr^F$_4$** were collected on a XtaLAB Synergy, Dualflex diffractometer with a HyPix area detector and multi-layer mirror monochromated Cu$_{K\alpha}$ radiation. The structures were solved using the intrinsic phasing method,[3] refined with the ShelXL program[4] and expanded using Fourier techniques. All non-hydrogen atoms were refined anisotropically. Hydrogen atoms were included in structure factor calculations. All hydrogen atoms were assigned to idealized geometric positions, except for those bound to N2, which were detected in the inverse Fourier map and refined freely.

Crystallographic data have been deposited with the Cambridge Crystallographic Data Center as supplementary publication nos. CCDC-2128816 (**3-OTf**), 2128817 (**3-BAr^F$_4$·Et$_2$O**), 2128818 (**3-OTf·HOTf**), 2128819 (**2**), 2128820 (**3-Cl**), 2128821 (**3-BAr^F$_4$**). These data can be obtained free of charge from The Cambridge Crystallographic Data Centre via www.ccdc.cam.ac.uk/data_request/cif.

Crystal data for **2**: $C_{60}H_{70}B_2Cu_2F_{10}N_4S_2$, M_r = 1419.87, red block, 0.356×0.307×0.176 mm^3, monoclinic space group $P2_1/n$, a = 9.88691(5) Å, b = 23.13953(13) Å, c = 13.87825(7) Å, β = 99.4769(5)°, V = 3131.71(3) Å^3, Z = 2, ρ_{calcd} = 1.506 g·cm^{-3}, μ = 3.654 mm^{-1}, F(000) = 1464, T = 100.0(3) K, R_1 = 0.0384, wR_2 = 0.1004, 6181 independent reflections [2θ≤144.252°] and 394 parameters.

Refinement details for **3-Cl**: One of the two CH_2Cl_2 molecules (RESI DCM2) in the asymmetric unit was modelled as twofold disordered in a 82:18 ratio. 1,2- and 1,3-distances in these residues were restrained with SAME and ADPs with SIMU 0.005.

Crystal data for **3-Cl**: $[C_{48}H_{72}B_2N_4S_2]Cl\cdot(CH_2Cl_2)_4$, M_r = 1201.44, red block, 0.387×0.228×0.21 mm^3, triclinic space group $P\bar{1}$, a = 9.349(3) Å, b = 11.782(3) Å, c = 15.284(3) Å, α = 100.027(14)°, β = 106.935(13)°, γ = 105.492(13)°, V = 1493.1(7) Å^3, Z = 1, ρ_{calcd} = 1.336 g·cm^{-3}, μ = 0.575 mm^{-1}, F(000) = 632, T = 100(2) K, R_1 = 0.0389, wR_2 = 0.0896, 5851 independent reflections [$2\theta \leq 52.04°$] and 354 parameters.

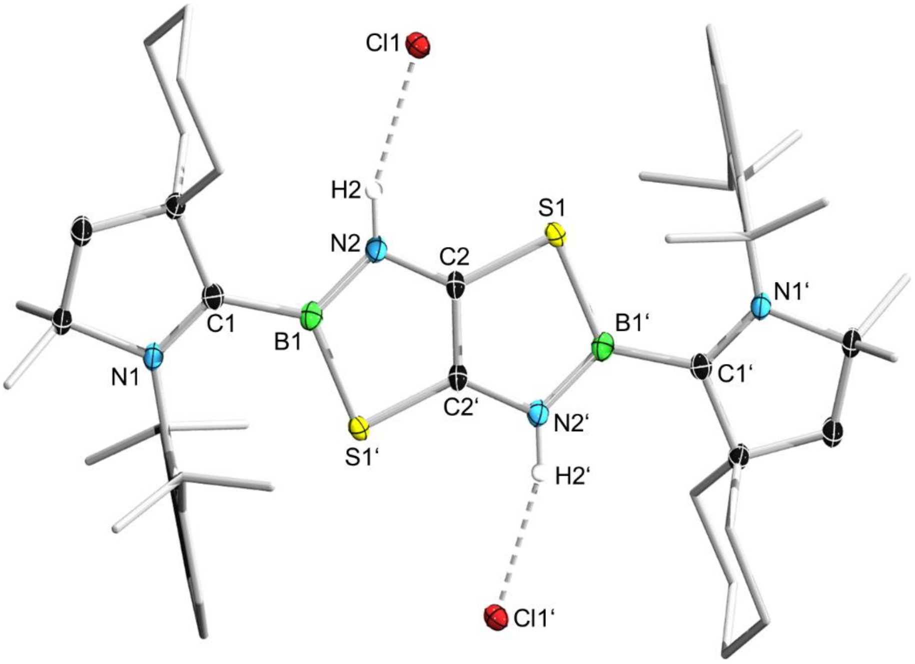

Figure S51. Crystallographically-derived solid-state structure of **3-Cl**. Atomic displacement ellipsoids drawn at 50% probability. Ellipsoids on ligand periphery and hydrogen atoms (except H bound to N2/N2') omitted for clarity.

Refinement details for **3-OTf**: The most disagreeable reflection (-4 -4 14) was omitted. The CAAC ligand was modelled as twofold disordered in the backbone and cyclohexyl group (RESI 21 and 22 DIS) in a 89:11 ratio. 1,2- and 1,3-distances within this disorder were restrained with SAME, ADPs with SIMU 0.005.

Crystal data for **3-OTf**: $[C_{48}H_{72}B_2N_4S_2][CF_3O_3S]_2$, M_r = 1088.97, orange plate, 0.096×0.054×0.032 mm^3, triclinic space group $P\bar{1}$, a = 9.4490(3) Å, b = 10.6687(3) Å, c = 13.2878(4) Å, α = 80.966(2), β = 89.074(2)°, γ = 86.747(2)°, V = 1320.74(7) Å^3, Z = 1, ρ_{calcd} = 1.369 g·cm^{-3}, μ = 2.272 mm^{-1}, F(000) = 576, T = 99.9(8) K, R_1 = 0.0679, wR_2 = 0.1484, 5209 independent reflections [$2\theta \leq 144.250°$] and 424 parameters.

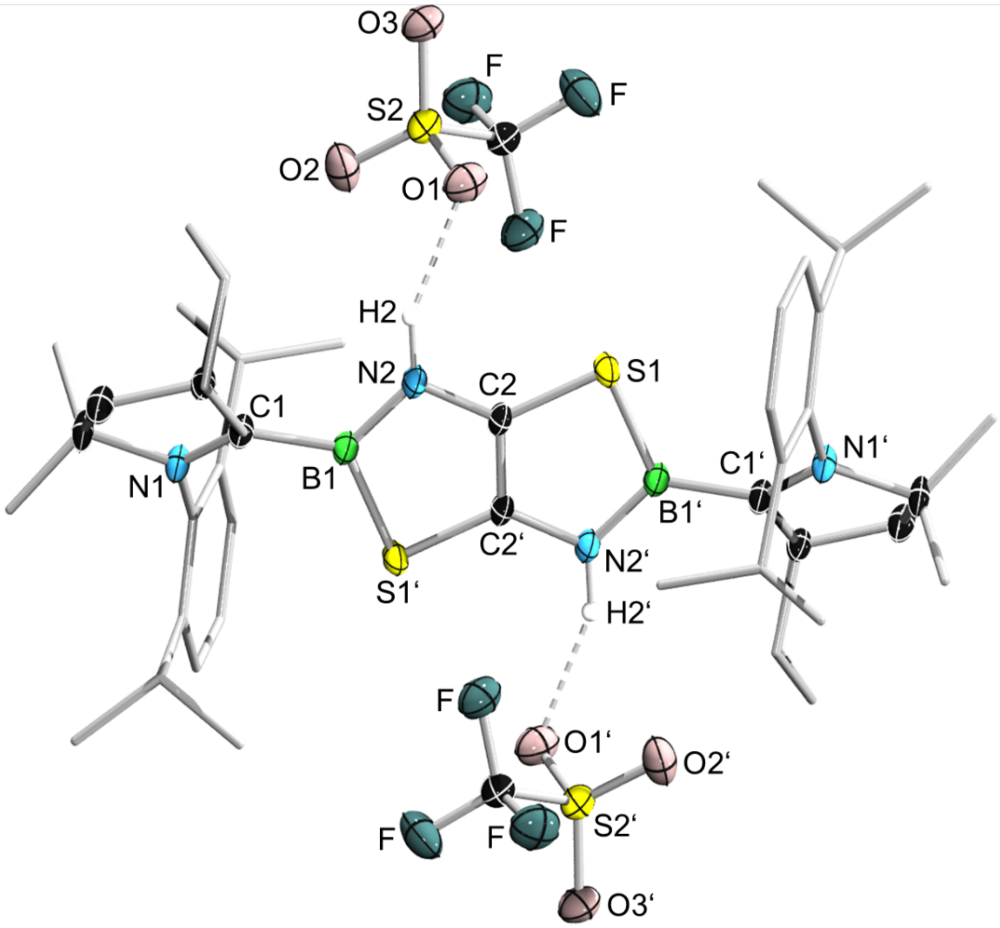

Figure S52. Crystallographically-derived solid-state structure of **3-OTf**. Atomic displacement ellipsoids drawn at 50% probability. Ellipsoids on ligand periphery and hydrogen atoms (except H bound to N2/N2’) omitted for clarity.

Refinement details for **3-OTf·HOTf**: The HOTf molecule hydrogen-bonded to the OTf^{-} anion was modelled as twofold disordered in a 74:26 ratio (RESI 6 and 61 OTf2). 1,2- and 1,3-distances within both residues were restrained to similarity with SAME, ADPs with SIMU 0.005.

Crystal data for **3-OTf·HOTf**: $[C_{48}H_{72}B_2N_4S_2][CF_3O_3S]_2{\cdot}(CHF_3O_3S)_2$, $M_r = 1558.98$, yellow block, 0.365×0.34×0.304 mm^3, triclinic space group $P\bar{1}$, $a = 9.577(4)$ Å, $b = 10.746(4)$ Å, $c = 17.956(12)$ Å, $\alpha = 78.701(9)°$, $\beta = 77.36(2)°$, $\gamma = 88.871(9)°$, $V = 1767.7(16)$ Å^3, $Z = 1$, $\rho_{calcd} = 1.464$ g·cm^{-3}, $\mu = 0.434$ mm^{-1}, $F(000) = 808$, $T = 101(2)$ K, $R_1 = 0.0556$, $wR_2 = 0.0930$, 6964 independent reflections [$2\theta \leq 52.042°$] and 509 parameters.

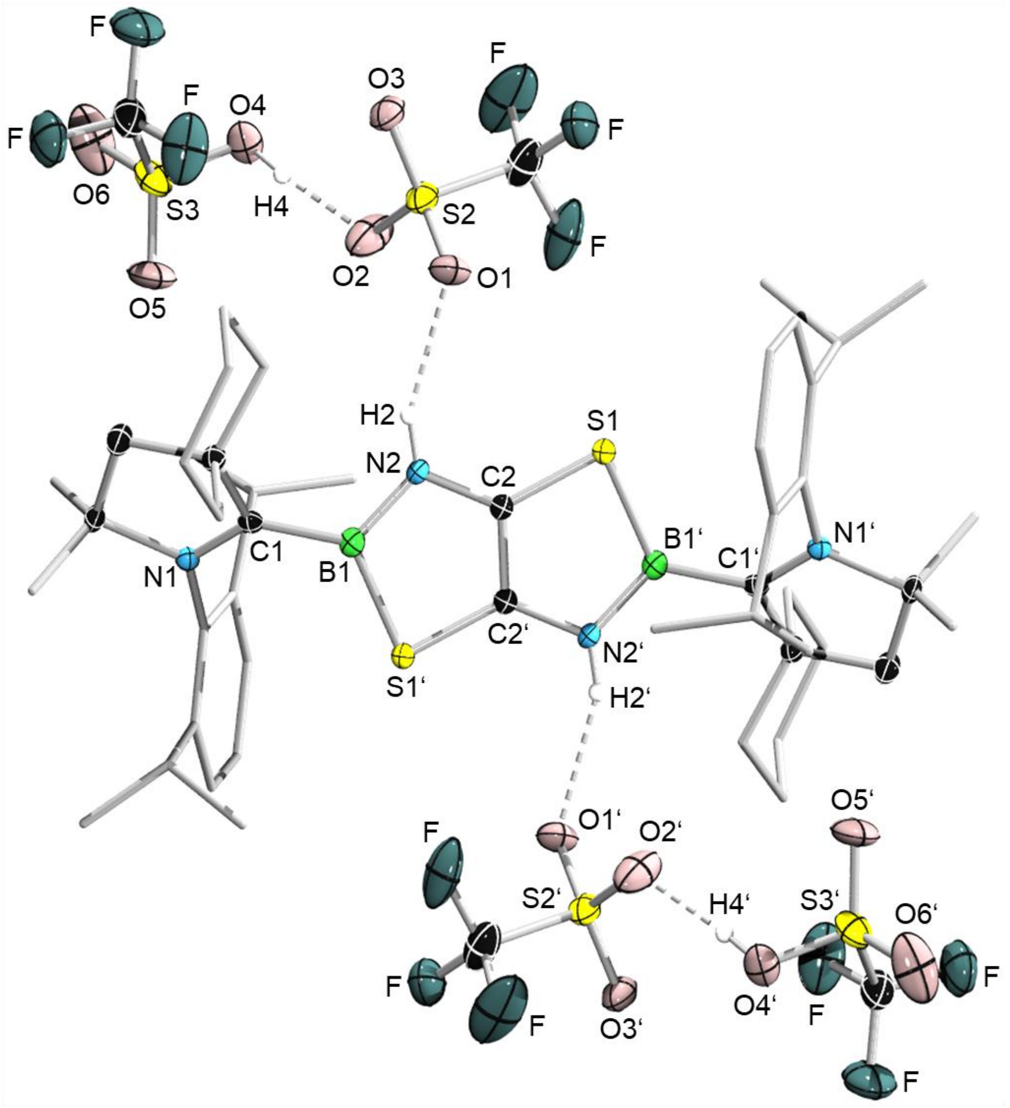

Figure S53. Crystallographically-derived solid-state structure of **3-OTf·HOTf**. Atomic displacement ellipsoids drawn at 50% probability. Ellipsoids on ligand periphery and hydrogen atoms (except H bound to N2/N2' and H2) omitted for clarity.

Refinement details for **3-BAr^F$_4$·Et$_2$O**: Two of the CF_3 groups of the $BAr^F{}_4^-$ anion were modelled as twofold disordered, one in F_3 only (RESI 21 and 22 F3), the other in CF_3 (RESI 31 and 32 CF3). 1,2- and 1,3-distances in these residues were restrained to similarity with SAME. ADPs in RESI F3 were restrained with SIMU 0.003 to similarity with the adjacent carbon atom C9_1. ADPs in RESI CF3 were restrained with SIMU 0.005.

Crystal data for **3-BAr^F$_4$·Et$_2$O**: $[C_{48}H_{72}B_2N_4S_2][C_{32}H_{12}BF_{24}]_2\cdot(C_4H_{10}O)_4$, M_r = 2813.76, orange block, 0.289×0.265×0.132 mm^3, triclinic space group $P\bar{1}$, a = 13.254(7) Å, b = 14.011(7) Å, c = 19.110(10) Å, α = 103.44(2)°, β = 101.23(4)°, γ = 100.81(4)°, V = 3283(3) Å^3, Z = 1, ρ_{calcd} = 1.423 g·cm^{-3}, μ = 0.162 mm^{-1}, F(000) = 1448, T = 98(2) K, 0.0874, wR_2 = 0.1571, 12939 independent reflections [$2\theta \leq 52.042°$] and 935 parameters.

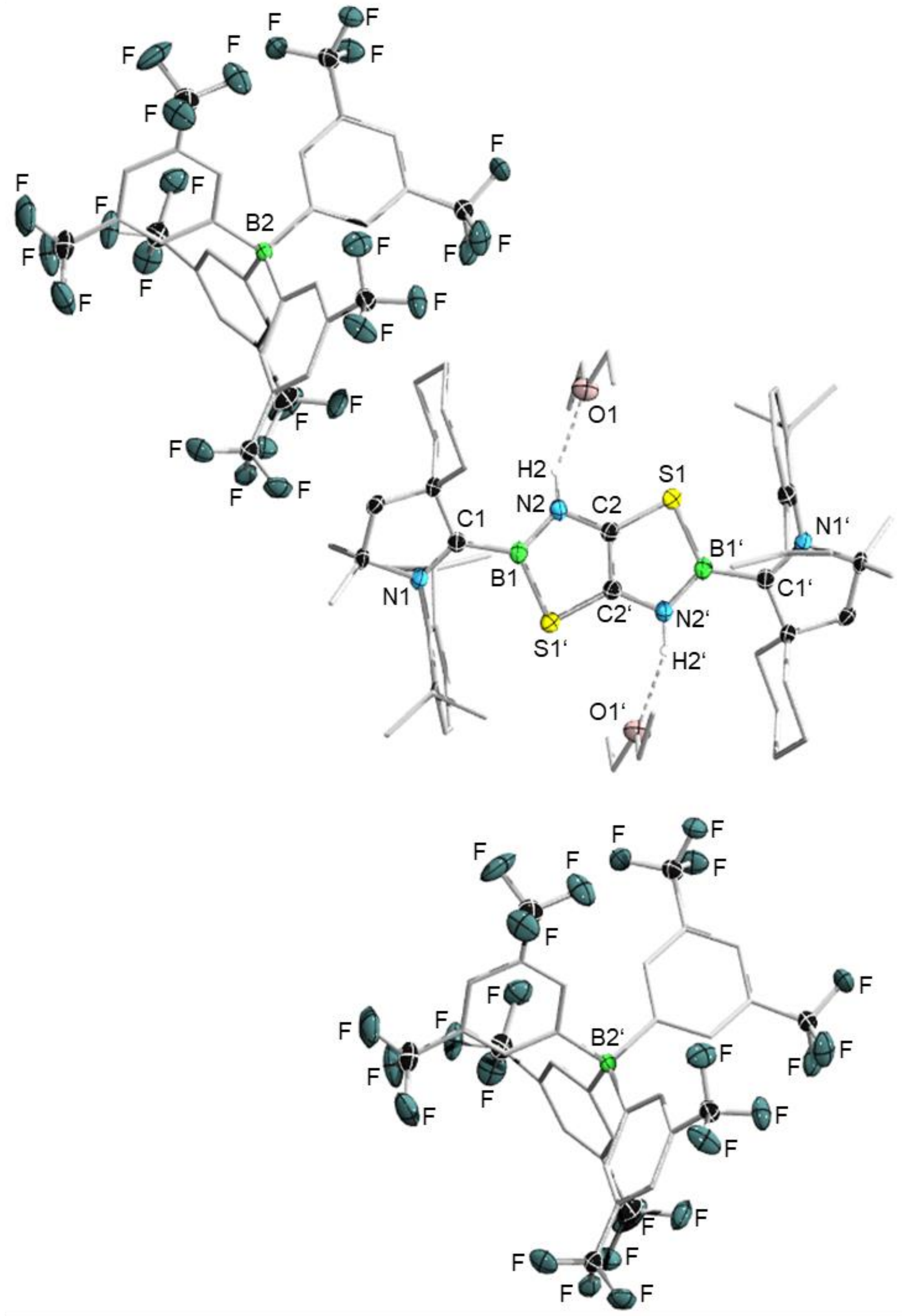

Figure S54. Crystallographically-derived solid-state structure of **3-BAr^{F}$_{4}$·Et$_2$O**. Atomic displacement ellipsoids drawn at 50% probability. Ellipsoids on ligand periphery and hydrogen atoms (except H bound to N2/N2’) omitted for clarity.

Refinement details for **3-BAr^F$_4$**: Two of the CF_3 groups of the $BAr^F{}_4{}^-$ anion were modelled as twofold disordered in the three fluorides: RESI 10 & 110 in a 90:10 ratio and RESI 11 & 111 in a 93:7 ratio. Two of the CF_3 groups of the $BAr^F{}_4$ anion were modelled as threefold disordered in the three fluorides, with three FVAR summed up to 1: RESI 9, 19 & 29 in a 53:20:27 ratio and RESI 8, 18 & 28 in a 62:18:20 ratio. 1,2- and 1,3-distances in these residues were restrained to similarity with SAME. ADPs in RESI 10 & 110 were restrained with SIMU 0.005 and ISOR 0.005 to similarity with the adjacent carbon atom C8_5. ADPs in RESI 11 & 111 were restrained with SIMU 0.005 to similarity with the adjacent carbon atom C8_4. ADPs in RESI 8, 18 & 28 were restrained with SIMU 0.005 and ISOR 0.003 to similarity with the adjacent carbon atom C7_7. ADPs in RESI 9, 19 & 29 were restrained with SIMU 0.005 and ISOR 0.001 to similarity with the adjacent carbon atom C8_7.

Crystal data for **3-BAr^F$_4$**: $[C_{48}H_{72}B_2N_4S_2][C_{32}H_{12}BF_{24}]_2$, M_r = 2517.28, orange block, 0.415×0.143×0.100 mm^3, triclinic space group $P\bar{1}$, a = 13.69288(11) Å, b = 13.92081(11) Å, c = 17.10886(11) Å, α = 101.5941(6)°, β = 107.8323(7)°, γ = 106.7535(7)°, V = 2819.14(4) Å^3, Z = 1, ρ_{calcd} = 1.483 g·cm^{-3}, μ = 1.590 mm^{-1}, F(000) = 1280, T = 99.9(6) K, R_1 = 0.0466, wR_2 = 0.1076, 11100 independent reflections [$2\theta \leq 144.256°$] and 945 parameters.

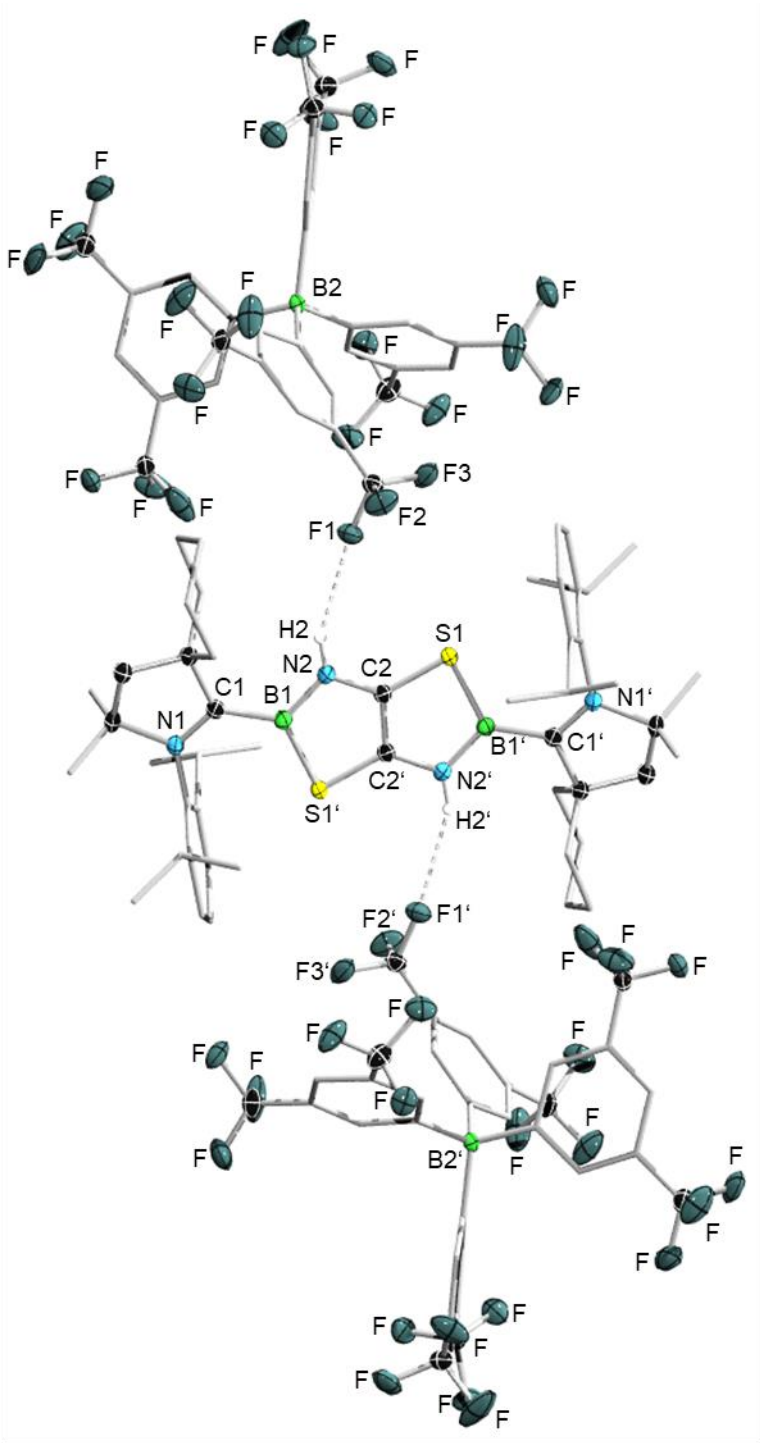

Figure S55. Crystallographically-derived solid-state structure of **3-BAr^{F}$_{4}$**. Atomic displacement ellipsoids drawn at 50% probability. Ellipsoids on ligand periphery and hydrogen atoms (except H bound to N2/N2') omitted for clarity.

Computational details

Geometry optimizations and Hessian calculations were performed for **1'**, **2**, **3**, **3-Cl**, and **3-OTf·HOTf** at the ωB97X-D[5]/def2-SVP[6] level of theory. All optimized structures were characterized as minimum energy geometries as only positive eigenvalues were obtained in the vibrational frequency calculations. Vertical excitation energies were computed for the aforementioned systems at the TD-DFT level (ωB97X-D/def2-SVP) in order to simulate their UV-vis spectra. A similar calculation was also performed for a model structure of **3-BAr$^{F}_{4}$·Et$_2$O**, as its large size prohibited us from performing a full geometry optimization. This structure was built by optimizing only the protonated TzbTzb unit and the additional two interacting Et_2O molecules of a monomer of the crystal structure of **3-BAr$^{F}_{4}$·Et$_2$O**. For selected cases, $S_0 \rightarrow S_1$ adiabatic excitation energies were also computed. The intramolecular charge transfer (ICT) character of the excitations was estimated using the interfragment charge transfer (IFCT)[7-9] and the Λ diagnostic[10] approaches. The aromatic character of **1'**, **2**, **3**, and **3-Cl** was probed using nucleus-independent chemical shift (NICS)[11-14] calculations at the ωB97X-D/def2-SVP level of theory. NICS(0), NICS(1), and $NICS_{zz}(1)$ values were obtained by placing ghost atoms at the ring centroid and at positions 1 Å above and below the ring. These calculations were done using the gauge-independent atomic orbital (GIAO) method.[15-17] For **3-Cl**, the anisotropy of the induced current density (ACID) plot was also obtained.[18] All DFT, TD-DFT, and NICS computations were performed using Gaussian 16, revision C.01.[19] The current density plot was obtained using the ACID software package, while the IFCT and Λ diagnostic calculations were performed using Multiwfn 3.8.[7]

Table S1. Comparison of the calculated geometrical properties of the S_0 and S_1 states of **1'**, **3**, and **3-Cl** at the level of theory (bond distances in Å, dihedral angle in °). The root-mean-square deviation (RMSD) values are also reported and were calculated considering all elements.

	**1' (S_0)**	**1' (S_1)**	**3 (S_0)**	**3(S_1)**	**3-Cl(S_0)**	**3-Cl (S_1)**
N1–C1	1.326	1.342	1.298	1.324	1.310	1.334
C1–B1	1.550	1.549	1.589	1.548	1.578	1.555
B1–N2	1.404	1.430	1.418	1.445	1.407	1.435
N2–C2	1.328	1.302	1.378	1.352	1.361	1.333
C2–C2'	1.421	1.461	1.363	1.412	1.376	1.423
C2'–S1	1.749	1.756	1.739	1.715	1.732	1.720
B1–S1	1.866	1.882	1.828	1.884	1.840	1.880
N2–H2	-	-	1.013	1.014	1.081	1.103
H2···Y	-	-	-	-	1.868	1.803
RMSD	-	0.054	-	0.135	-	0.046
N1-C1-B1-N2	168.3	167.5	161.5	166.1	164.5	165.8

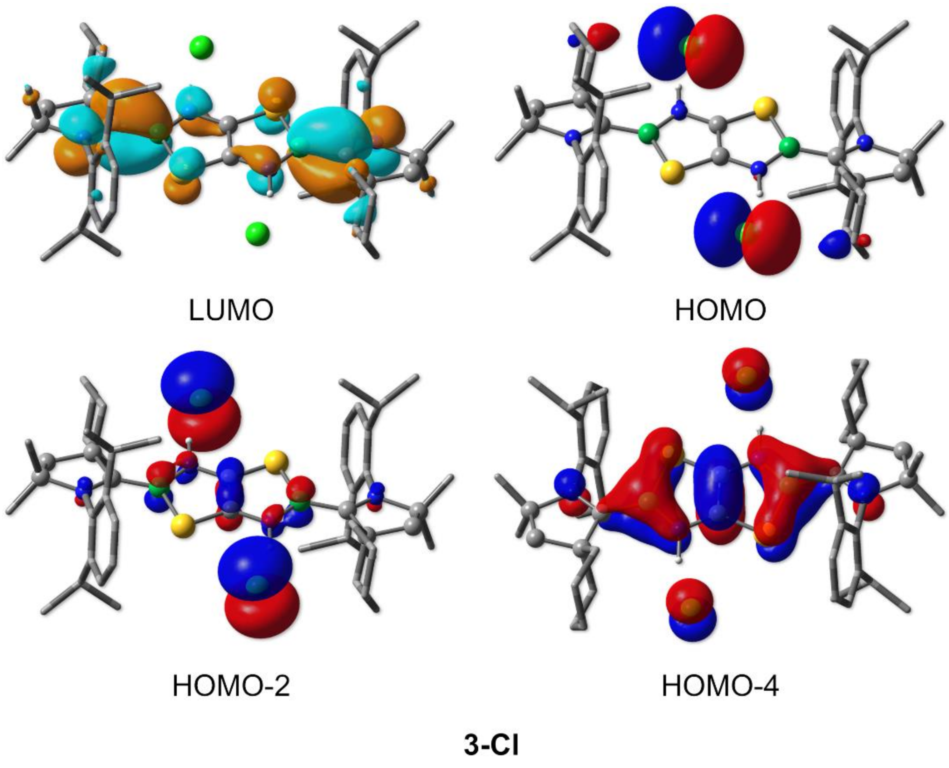

Figure S56. Canonical molecular orbitals involved in the $S_0 \rightarrow S_1$ electronic excitation of **3-Cl**.

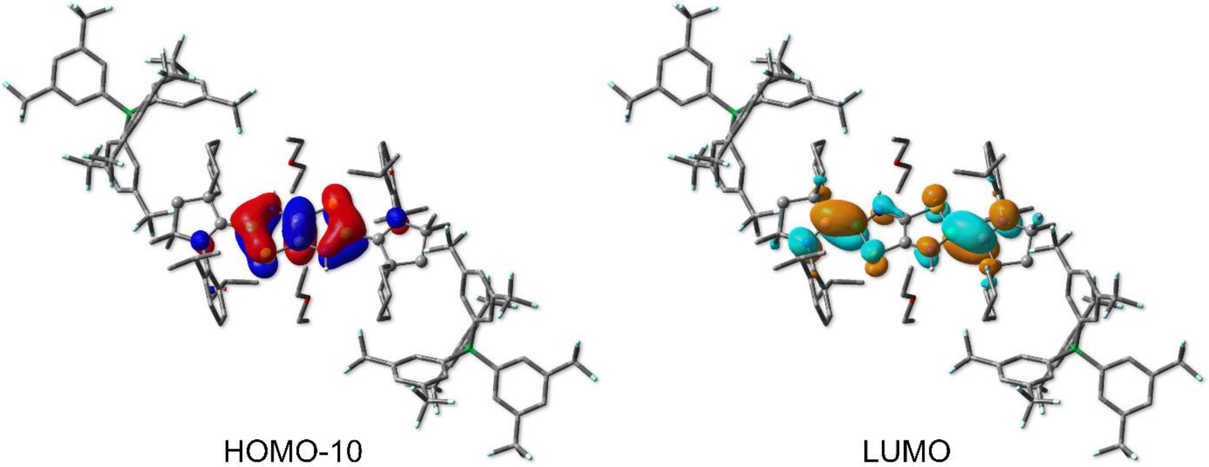

3-BAr$^{F}_{4}$·Et$_2$O

Figure S57. Canonical molecular orbitals involved in the $S_0 \rightarrow S_1$ electronic excitation of **3-BAr$^{F}_{4}$·Et$_2$O**.

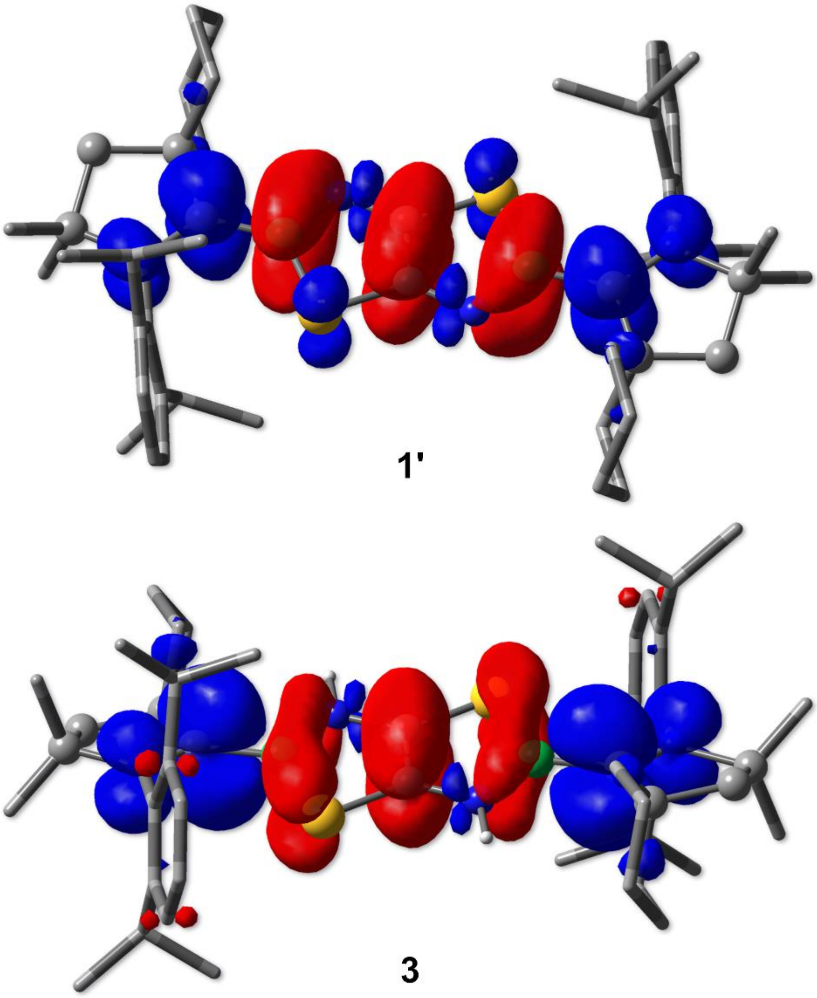

Figure S59. Charge density difference between the S_0 and S_1 states of **1'** (top) and **3** (bottom). Charge flows from red to blue following electronic excitation. Isosurface: 0.001.

Cartesian coordinates (Å)

1' (S_0)			
B	-2.230493133	-0.397934010	-0.101295949
S	-1.592881073	1.351512111	-0.219660957
N	-1.195676063	-1.335912087	0.039253061
C	-0.025866977	-0.707411047	0.062542063
C	-3.708818243	-0.863056036	-0.100470949
N	-4.794515313	-0.106926970	-0.007602942
C	-6.089445438	-0.865054018	0.063536063
C	-5.633792393	-2.260436119	-0.377687969
H	-5.850954407	-2.393471126	-1.449004044
H	-6.183388423	-3.044929172	0.159089070
C	-4.106592282	-2.329285138	-0.155063953
C	-6.651145444	-0.863347014	1.488186164
H	-7.576197547	-1.457260045	1.506240168
H	-6.899936488	0.156067064	1.810416188
H	-5.954330399	-1.303017048	2.212643217
C	-7.152461511	-0.284921967	-0.865807006
H	-8.076721542	-0.869572001	-0.751843997
H	-6.852225443	-0.339326973	-1.918924078
H	-7.379891521	0.761510112	-0.614392984
C	-3.713360261	-3.017086188	1.179712145
H	-2.630231182	-2.871802183	1.305467155
H	-4.206427291	-2.514448147	2.027377203
C	-4.039234294	-4.510657292	1.183703142
H	-3.690212270	-4.952533328	2.130230210
H	-5.132535372	-4.667629298	1.159923144
C	-3.398400254	-5.227682352	-0.004417942
H	-2.301008171	-5.194628353	0.107153066
H	-3.686375280	-6.291062446	-0.010303942
C	-3.779263276	-4.560044299	-1.325801039
H	-4.862133355	-4.688405300	-1.507100049
H	-3.267083242	-5.056690339	-2.164691095

C	-3.410466236	-3.077413194	-1.313090038
H	-3.677160251	-2.597636160	-2.269784104
H	-2.325257157	-2.963474194	-1.183313027
C	-4.742083299	1.329922134	0.084923064
C	-4.562706282	1.943515177	1.344168157
C	-4.536029272	3.339737273	1.389422157
H	-4.397100257	3.838465309	2.351021226
C	-4.650812273	4.104127332	0.236466075
H	-4.619172262	5.193990409	0.297115080
C	-4.774633288	3.479299290	-0.996896011
H	-4.823973288	4.086427332	-1.903363081
C	-4.812773299	2.086959186	-1.104582020
C	-4.869555309	1.468211141	-2.496365123
H	-4.949463322	0.378573065	-2.380507114
C	-6.086343401	1.955939185	-3.293152181
H	-6.173904402	1.396250147	-4.237170245
H	-5.988392375	3.021099262	-3.554390198
H	-7.026035457	1.837731185	-2.735647137
C	-3.580450214	1.733769155	-3.284991177
H	-3.633033224	1.242755116	-4.269219251
H	-2.698272152	1.357820121	-2.750934141
H	-3.435334196	2.812407228	-3.455034190
C	-4.330990270	1.175605116	2.640213248
H	-4.408587287	0.103996042	2.419064233
C	-5.374492366	1.522873151	3.709850323
H	-5.264481343	0.858157103	4.580390386
H	-6.405540419	1.432135154	3.339821301
H	-5.244392330	2.555598224	4.069244349
C	-2.918776168	1.408032125	3.194587288
H	-2.771508161	0.808669082	4.106329355
H	-2.765127152	2.465968198	3.460043308
H	-2.148281118	1.129504099	2.464875235
B	2.230482194	0.398035015	0.100943066
S	1.592859133	-1.351408106	0.219308074

N	1.195664125	1.336001090	-0.039716944
C	0.025853037	0.707507056	-0.062961946
C	3.708817306	0.863118039	0.099942066
N	4.794500375	0.106940975	0.007203059
C	6.089440464	0.865045022	-0.064165946
C	5.633852455	2.260462123	0.376990085
H	5.851053469	2.393565134	1.448291163
H	6.183453512	3.044903179	-0.159856953
C	4.106643342	2.329341141	0.154397070
C	6.650930493	0.863293017	-1.488898050
H	7.575867605	1.457382053	-1.507147051
H	6.899872510	-0.156100059	-1.811066070
H	5.953912455	1.302763054	-2.213285103
C	7.152606559	0.284981971	0.865050118
H	8.076891619	0.869535007	0.750805111
H	6.852595550	0.339580977	1.918220198
H	7.379903533	-0.761510107	0.613764105
C	3.713376321	3.017109192	-1.180363028
H	2.630237241	2.871856191	-1.306080037
H	4.206391349	2.514423154	-2.028033087
C	4.039290355	4.510670299	-1.184414028
H	3.690235332	4.952531333	-2.130935096
H	5.132597434	4.667608301	-1.160695023
C	3.398539311	5.227750356	0.003718059
H	2.301140232	5.194730357	-0.107795949
H	3.686550342	6.291120444	0.009555059
C	3.779445334	4.560142302	1.325105156
H	4.862328415	4.688477305	1.506347167
H	3.267320303	5.056832344	2.164004213
C	3.410596298	3.077525199	1.312455152
H	3.677330312	2.597757161	2.269144223
H	2.325379217	2.963608197	1.182738145
C	4.742042362	-1.329958129	-0.084552948
C	4.562894347	-1.944328168	-1.343452038

C	4.536176330	-3.340583269	-1.387851041
H	4.397420319	-3.839893304	-2.349174111
C	4.650710337	-4.104259325	-0.234406958
H	4.619037328	-5.194159407	-0.294383963
C	4.774321348	-3.478675284	0.998598131
H	4.823448349	-4.085259327	1.905439197
C	4.812484363	-2.086277182	1.105440139
C	4.869000371	-1.466693138	2.496865238
H	4.948852382	-0.377116060	2.380394230
C	6.085722464	-1.953829181	3.294116294
H	6.173087458	-1.393569145	4.237812365
H	5.987857429	-3.018843258	3.555973316
H	7.025471513	-1.835840181	2.736657257
C	3.579792277	-1.731892147	3.285443296
H	3.632165283	-1.240287113	4.269387366
H	2.697664212	-1.356354115	2.751014259
H	3.434753255	-2.810445224	3.456098307
C	4.331472335	-1.177218112	-2.640027131
H	4.409106347	-0.105475037	-2.419534116
C	5.375145424	-1.525287146	-3.709241209
H	5.265384405	-0.861136096	-4.580243269
H	6.406134473	-1.434433149	-3.339073182
H	5.244998391	-2.558239221	-4.067967233
C	2.919354231	-1.409902121	-3.194532174
H	2.772293222	-0.811088078	-4.106669235
H	2.765690212	-2.467988194	-3.459380190
H	2.148744175	-1.130894094	-2.465123117

**1' (S_1)**			
B	-2.238427000	-0.402846000	-0.096547000
S	-1.599826000	1.360270000	-0.259698000
N	-1.173194000	-1.343100000	0.068802000
C	-0.027185000	-0.725635000	0.079996000
C	-3.715463000	-0.868222000	-0.111165000

N	-4.813203000	-0.100337000	-0.025238000
C	-6.098466000	-0.863947000	0.035037000
C	-5.637281000	-2.254398000	-0.421419000
H	-5.838657000	-2.368666000	-1.498130000
H	-6.196852000	-3.046804000	0.093802000
C	-4.113024000	-2.329866000	-0.179218000
C	-6.665437000	-0.883068000	1.459875000
H	-7.587185000	-1.482904000	1.471197000
H	-6.920249000	0.132273000	1.791335000
H	-5.967114000	-1.324734000	2.181441000
C	-7.169521000	-0.282382000	-0.886051000
H	-8.091907000	-0.870540000	-0.773029000
H	-6.873681000	-0.326420000	-1.941052000
H	-7.399455000	0.761107000	-0.624434000
C	-3.739736000	-3.033810000	1.154279000
H	-2.658853000	-2.889093000	1.303668000
H	-4.249102000	-2.541175000	1.997646000
C	-4.059000000	-4.529441000	1.140581000
H	-3.724619000	-4.979085000	2.088996000
H	-5.151391000	-4.688267000	1.096506000
C	-3.397357000	-5.233702000	-0.043352000
H	-2.301573000	-5.195983000	0.083598000
H	-3.679128000	-6.298648000	-0.062012000
C	-3.764803000	-4.556915000	-1.363594000
H	-4.845114000	-4.687118000	-1.557812000
H	-3.241294000	-5.044932000	-2.200838000
C	-3.404552000	-3.071821000	-1.335106000
H	-3.664704000	-2.586274000	-2.290402000
H	-2.319155000	-2.959917000	-1.198299000
C	-4.756424000	1.328124000	0.112046000
C	-4.547355000	1.903290000	1.386228000
C	-4.512023000	3.297188000	1.476346000
H	-4.351314000	3.764018000	2.450720000
C	-4.648752000	4.099639000	0.351611000

H	-4.611453000	5.186921000	0.447037000
C	-4.801570000	3.515331000	-0.898535000
H	-4.866619000	4.151796000	-1.783881000
C	-4.847000000	2.127164000	-1.049713000
C	-4.924678000	1.549818000	-2.457766000
H	-5.003676000	0.457806000	-2.368421000
C	-6.150581000	2.060445000	-3.224960000
H	-6.248133000	1.531016000	-4.185394000
H	-6.058117000	3.133807000	-3.453051000
H	-7.083347000	1.921121000	-2.660834000
C	-3.645188000	1.834815000	-3.254922000
H	-3.712008000	1.374321000	-4.252987000
H	-2.756867000	1.439133000	-2.745254000
H	-3.496251000	2.917487000	-3.393891000
C	-4.298823000	1.091196000	2.651674000
H	-4.380203000	0.028203000	2.392818000
C	-5.326718000	1.401215000	3.747301000
H	-5.200459000	0.711141000	4.595718000
H	-6.362284000	1.315294000	3.389624000
H	-5.196172000	2.423620000	4.135242000
C	-2.879221000	1.303759000	3.194509000
H	-2.716371000	0.668351000	4.078750000
H	-2.721615000	2.350357000	3.499849000
H	-2.118274000	1.057006000	2.443645000
B	2.238443000	0.402721000	0.095890000
S	1.599865000	-1.360391000	0.259064000
N	1.173216000	1.342985000	-0.069408000
C	0.027208000	0.725513000	-0.080619000
C	3.715471000	0.868142000	0.110715000
N	4.813240000	0.100338000	0.024526000
C	6.098421000	0.864090000	-0.035810000
C	5.637252000	2.254242000	0.421575000
H	5.838803000	2.367858000	1.498324000
H	6.196698000	3.046989000	-0.093257000

C	4.112948000	2.329771000	0.179632000
C	6.664744000	0.884125000	-1.460901000
H	7.586278000	1.484289000	-1.472345000
H	6.919755000	-0.130944000	-1.793028000
H	5.965913000	1.325928000	-2.181897000
C	7.169913000	0.282091000	0.884486000
H	8.092272000	0.870247000	0.771229000
H	6.874632000	0.325719000	1.939658000
H	7.399666000	-0.761306000	0.622342000
C	3.739383000	3.034802000	-1.153195000
H	2.658497000	2.890081000	-1.302544000
H	4.248687000	2.542963000	-1.997058000
C	4.058485000	4.530457000	-1.138263000
H	3.723932000	4.980872000	-2.086250000
H	5.150864000	4.689370000	-1.094193000
C	3.396925000	5.233649000	0.046350000
H	2.301126000	5.195902000	-0.080472000
H	3.678571000	6.298613000	0.065863000
C	3.764648000	4.555797000	1.365970000
H	4.844974000	4.685958000	1.560133000
H	3.241208000	5.043041000	2.203708000
C	3.404568000	3.070685000	1.336274000
H	3.664913000	2.584350000	2.291116000
H	2.319160000	2.958791000	1.199529000
C	4.756530000	-1.328142000	-0.112532000
C	4.547487000	-1.903587000	-1.386574000
C	4.512135000	-3.297510000	-1.476381000
H	4.351429000	-3.764556000	-2.450654000
C	4.648820000	-4.099704000	-0.351463000
H	4.611495000	-5.187006000	-0.446646000
C	4.801649000	-3.515116000	0.898557000
H	4.866710000	-4.151378000	1.784048000
C	4.847111000	-2.126920000	1.049414000
C	4.924913000	-1.549223000	2.457316000

H	5.004215000	-0.457255000	2.367698000
C	6.150688000	-2.059966000	3.224634000
H	6.248445000	-1.530254000	4.184891000
H	6.057919000	-3.133227000	3.453084000
H	7.083466000	-1.921110000	2.660405000
C	3.645329000	-1.833666000	3.254516000
H	3.712267000	-1.372986000	4.252488000
H	2.757134000	-1.437828000	2.744747000
H	3.496063000	-2.916266000	3.393692000
C	4.299011000	-1.091741000	-2.652174000
H	4.380337000	-0.028708000	-2.393465000
C	5.327018000	-1.401944000	-3.747643000
H	5.200864000	-0.712016000	-4.596193000
H	6.362546000	-1.315974000	-3.389853000
H	5.196520000	-2.424421000	-4.135412000
C	2.879454000	-1.304423000	-3.195065000
H	2.716667000	-0.669211000	-4.079457000
H	2.721860000	-2.351087000	-3.500186000
H	2.118469000	-1.057492000	-2.444290000

2

B	0.565038000	-2.215882000	0.127349000
N	-0.722389000	-1.649983000	0.013113000
C	-0.625559000	-0.294565000	-0.043819000
S	-1.868901000	0.905500000	-0.202098000
Cu	-2.586827000	-2.152695000	0.054988000
F	-4.699779000	-3.705156000	-1.733307000
F	-7.365818000	-3.374804000	-1.867609000
C	-4.497533000	-2.199112000	0.080494000
F	-8.617440000	-1.658105000	-0.168590000
C	-5.275155000	-2.860946000	-0.858101000
F	-7.183055000	-0.307518000	1.700410000
C	-6.658983000	-2.718078000	-0.952581000
F	-4.529574000	-0.669588000	1.889619000

C	-6.565089000	-1.161344000	0.880262000
C	-5.187577000	-1.352984000	0.937058000
C	-7.304870000	-1.839943000	-0.084756000
C	0.874265000	-3.760125000	0.129991000
N	2.068085000	-4.292966000	-0.016020000
C	-0.170291000	-4.854198000	0.287768000
C	0.691263000	-6.128278000	0.438977000
H	0.788594000	-6.381630000	1.505905000
H	0.251460000	-7.001486000	-0.059010000
C	2.071132000	-5.798576000	-0.132185000
C	2.201683000	-6.221393000	-1.598368000
H	1.418113000	-5.784267000	-2.230191000
H	2.111468000	-7.315398000	-1.655521000
H	3.180381000	-5.943753000	-2.007595000
C	3.205815000	-6.440739000	0.658774000
H	3.163629000	-7.528130000	0.503285000
H	3.116298000	-6.256037000	1.735115000
H	4.189141000	-6.086057000	0.317877000
C	-2.083126000	-5.699611000	1.731144000
H	-1.612657000	-6.687188000	1.886349000
H	-2.665332000	-5.488862000	2.641172000
C	-3.003839000	-5.744364000	0.512965000
H	-3.547029000	-4.788508000	0.440729000
H	-3.762375000	-6.533155000	0.630668000
C	-2.219114000	-5.954411000	-0.780485000
H	-2.896861000	-5.885289000	-1.643770000
H	-1.792675000	-6.973058000	-0.798383000
C	-1.110138000	-4.914640000	-0.944020000
H	-1.572174000	-3.925023000	-1.084706000
H	-0.527265000	-5.112410000	-1.856486000
C	-1.009750000	-4.625188000	1.566035000
H	-0.340087000	-4.594597000	2.441750000
H	-1.496450000	-3.639609000	1.512581000
C	3.299436000	-3.534102000	-0.023272000

C	3.829149000	-3.041614000	-1.233726000
C	5.019445000	-2.312251000	-1.168646000
H	5.446436000	-1.895342000	-2.081561000
C	5.648700000	-2.060246000	0.041915000
H	6.572418000	-1.479751000	0.058870000
C	5.089097000	-2.526566000	1.224147000
H	5.576313000	-2.295817000	2.173524000
C	3.904358000	-3.265188000	1.224979000
C	3.156496000	-3.205665000	-2.590160000
H	2.247449000	-3.805028000	-2.452100000
C	3.317024000	-3.695458000	2.565706000
H	2.459339000	-4.355899000	2.373090000
C	2.714592000	-1.861468000	-3.184695000
H	2.226448000	-2.028222000	-4.157250000
H	3.572191000	-1.192023000	-3.343498000
H	2.008539000	-1.339627000	-2.526661000
C	4.071070000	-3.938902000	-3.580492000
H	3.522833000	-4.182179000	-4.503333000
H	4.478830000	-4.872737000	-3.166034000
H	4.927486000	-3.307396000	-3.862191000
C	4.331901000	-4.482481000	3.405226000
H	4.794365000	-5.302215000	2.836942000
H	3.842578000	-4.910254000	4.293538000
H	5.143322000	-3.830676000	3.763022000
C	2.782312000	-2.500045000	3.365562000
H	1.995481000	-1.964190000	2.819492000
H	3.585507000	-1.778515000	3.581682000
H	2.368747000	-2.843250000	4.326426000
B	-0.564984000	2.215865000	-0.126816000
N	0.722438000	1.649950000	-0.012585000
C	0.625603000	0.294530000	0.044280000
S	1.868936000	-0.905515000	0.202702000
C	-0.874228000	3.760112000	-0.129613000
N	-2.068085000	4.292919000	0.016174000

C	0.170295000	4.854214000	-0.287433000
C	-0.691302000	6.128246000	-0.438851000
H	-0.788532000	6.381475000	-1.505818000
H	-0.251577000	7.001527000	0.059080000
C	-2.071219000	5.798556000	0.132190000
C	-2.201980000	6.221515000	1.598312000
H	-1.418462000	5.784512000	2.230278000
H	-2.111839000	7.315529000	1.655351000
H	-3.180718000	5.943862000	2.007443000
C	-3.205840000	6.440600000	-0.658962000
H	-3.163697000	7.528009000	-0.503590000
H	-3.116216000	6.255779000	-1.735276000
H	-4.189191000	6.085936000	-0.318123000
C	2.083225000	5.699539000	-1.730724000
H	1.612740000	6.687082000	-1.886092000
H	2.665523000	5.488704000	-2.640674000
C	3.003823000	5.744448000	-0.512466000
H	3.547038000	4.788622000	-0.440088000
H	3.762338000	6.533258000	-0.630178000
C	2.218990000	5.954586000	0.780905000
H	2.896684000	5.885531000	1.644238000
H	1.792525000	6.973225000	0.798696000
C	1.110028000	4.914797000	0.944431000
H	1.572090000	3.925210000	1.085236000
H	0.527071000	5.112620000	1.856832000
C	1.009867000	4.625101000	-1.565597000
H	0.340285000	4.594384000	-2.441369000
H	1.496597000	3.639538000	-1.512004000
C	-3.299416000	3.534021000	0.023318000
C	-3.829293000	3.041649000	1.233750000
C	-5.019592000	2.312304000	1.168570000
H	-5.446749000	1.895468000	2.081446000
C	-5.648677000	2.060197000	-0.042065000
H	-6.572397000	1.479720000	-0.059073000

C	-5.088913000	2.526398000	-1.224263000
H	-5.575985000	2.295547000	-2.173689000
C	-3.904157000	3.264995000	-1.224997000
C	-3.156831000	3.205863000	2.590255000
H	-2.247718000	3.805137000	2.452230000
C	-3.316631000	3.695124000	-2.565693000
H	-2.458899000	4.355492000	-2.373042000
C	-2.715117000	1.861729000	3.185076000
H	-2.227249000	2.028588000	4.157754000
H	-3.572779000	1.192316000	3.343708000
H	-2.008886000	1.339805000	2.527309000
C	-4.071491000	3.939346000	3.580318000
H	-3.523363000	4.182756000	4.503187000
H	-4.479111000	4.873138000	3.165629000
H	-4.927996000	3.307963000	3.862020000
C	-4.331334000	4.482210000	-3.405361000
H	-4.793735000	5.302053000	-2.837185000
H	-3.841875000	4.909846000	-4.293665000
H	-5.142810000	3.830484000	-3.763176000
C	-2.781969000	2.499612000	-3.365434000
H	-1.995224000	1.963719000	-2.819281000
H	-3.585217000	1.778143000	-3.581560000
H	-2.368310000	2.842718000	-4.326294000
Cu	2.586837000	2.152777000	-0.054797000
F	4.700291000	3.705263000	1.733081000
F	7.366326000	3.374670000	1.866839000
C	4.497542000	2.199286000	-0.080722000
F	8.617452000	1.657892000	0.167538000
C	5.275420000	2.861027000	0.857731000
F	7.182583000	0.307487000	-1.701203000
C	6.659248000	2.718038000	0.951929000
F	4.529085000	0.669755000	-1.889853000
C	6.564852000	1.161355000	-0.880930000
C	5.187339000	1.353108000	-0.937430000

C	7.304885000	1.839861000	0.083955000

3 (S_0)

B	2.294525167	-0.390091028	0.041574003
S	1.630455118	1.271915090	0.412077030
N	1.214630086	-1.265035092	-0.240437017
C	-0.021326002	-0.659945049	-0.168819012
H	1.282704093	-2.246632160	-0.480491034
C	3.817995272	-0.842337058	0.020464001
N	4.818855345	-0.020900002	-0.067664005
C	6.174175447	-0.708039053	-0.186737013
C	5.808058399	-2.119935152	0.283289020
H	6.053792438	-2.226683162	1.350405099
H	6.389951439	-2.877336208	-0.255589018
C	4.283939306	-2.283347166	0.086502006
C	6.643060463	-0.665343048	-1.643162119
H	7.596723539	-1.206046085	-1.711384121
H	6.822596492	0.366117027	-1.971216141
H	5.938576422	-1.141316081	-2.336163167
C	7.239693523	-0.050974004	0.681277046
H	8.192001588	-0.566630039	0.496089035
H	7.024311503	-0.135359009	1.751917124
H	7.378237525	1.007742070	0.420439030
C	3.936132283	-3.032039219	-1.230627088
H	2.853850205	-2.963702211	-1.427115104
H	4.423141318	-2.541114182	-2.085915151
C	4.324661310	-4.510652323	-1.171281082
H	4.001742287	-5.001012362	-2.101357151
H	5.422403386	-4.608176330	-1.145479082
C	3.721081269	-5.210096373	0.045241003
H	2.621515188	-5.244637376	-0.059961004
H	4.058456293	-6.255657472	0.085814006
C	4.079375294	-4.484042322	1.340653097
H	5.166410373	-4.547555329	1.516949107

H	3.602361258	-4.972250356	2.202933158
C	3.644075264	-3.020011216	1.287697092
H	3.907966279	-2.496752179	2.221116159
H	2.545492182	-2.982152214	1.213439088
C	4.673104335	1.421953101	-0.096981007
C	4.408382316	2.063092149	-1.326101094
C	4.274345307	3.453460247	-1.301036095
H	4.078726295	3.986604288	-2.233383160
C	4.374135314	4.170428299	-0.115553008
H	4.269672306	5.256938376	-0.125191009
C	4.597479329	3.506127253	1.083524078
H	4.655246334	4.079531295	2.010562147
C	4.749164341	2.117726154	1.129738080
C	4.954087358	1.451934104	2.487313181
H	5.134107367	0.379593027	2.324420168
C	6.171918453	2.029756146	3.221653233
H	6.398013454	1.428639102	4.114514298
H	5.978667420	3.057320223	3.563763255
H	7.069173510	2.056397146	2.588889188
C	3.707815267	1.557421112	3.376872244
H	3.905785280	1.093779078	4.354506315
H	2.837719205	1.055305077	2.932841213
H	3.434465245	2.608204189	3.557670255
C	4.209398303	1.337960099	-2.652297190
H	4.393570317	0.267220019	-2.495281178
C	5.192875374	1.829328131	-3.722607268
H	5.131267368	1.194859087	-4.618794335
H	6.233583425	1.822641129	-3.369834245
H	4.960401358	2.858435203	-4.034592288
C	2.767089198	1.467149103	-3.163015229
H	2.660374192	0.939693067	-4.122437299
H	2.495830178	2.520257179	-3.331738240
H	2.037366149	1.043238074	-2.458739176
B	-2.294584165	0.390132028	-0.041575003

S	-1.630525118	-1.271917090	-0.411900030
N	-1.214697085	1.265052089	0.240545018
C	0.021259002	0.659958046	0.168939012
H	-1.282773092	2.246642162	0.480626034
C	-3.818058276	0.842360062	-0.020481001
N	-4.818883349	0.020890001	0.067704005
C	-6.174233445	0.707987051	0.186781013
C	-5.808178417	2.119861151	-0.283360021
H	-6.053934416	2.226516159	-1.350480099
H	-6.390091459	2.877282205	0.255469018
C	-4.284060308	2.283350166	-0.086606006
C	-6.643090467	0.665415048	1.643220117
H	-7.596796566	1.206050085	1.711389122
H	-6.822539499	-0.366020026	1.971401141
H	-5.938639447	1.141533081	2.336154166
C	-7.239773535	0.050832004	-0.681136047
H	-8.192092607	0.566460040	-0.495925036
H	-7.024456498	0.135160010	-1.751793128
H	-7.378261549	-1.007873073	-0.420223030
C	-3.936269282	3.032143218	1.230470086
H	-2.853981203	2.963872214	1.426947100
H	-4.423242317	2.541250181	2.085796149
C	-4.324874310	4.510733324	1.171033083
H	-4.001963288	5.001170359	2.101072152
H	-5.422621408	4.608201333	1.145244085
C	-3.721350266	5.210129373	-0.045544003
H	-2.621783190	5.244729379	0.059636004
H	-4.058776292	6.255671442	-0.086177006
C	-4.079633294	4.483975323	-1.340903097
H	-5.166675369	4.547422326	-1.517181108
H	-3.602662260	4.972152358	-2.203223158
C	-3.644259264	3.019969215	-1.287864095
H	-3.908146279	2.496638179	-2.221244162
H	-2.545673182	2.982171216	-1.213632089

C	-4.673047338	-1.421957102	0.097028007
C	-4.408250318	-2.063074146	1.326142094
C	-4.274177309	-3.453440250	1.301092092
H	-4.078498294	-3.986564288	2.233438163
C	-4.373962315	-4.170423298	0.115620009
H	-4.269449306	-5.256928380	0.125264009
C	-4.597324333	-3.506137255	-1.083463080
H	-4.655059333	-4.079549293	-2.010498143
C	-4.749062342	-2.117743151	-1.129688083
C	-4.953943358	-1.451973104	-2.487278181
H	-5.134034367	-0.379642027	-2.324406166
C	-6.171691436	-2.029875148	-3.221692233
H	-6.397758444	-1.428783102	-4.114578297
H	-5.978359439	-3.057433218	-3.563774257
H	-7.068991526	-2.056558148	-2.588992187
C	-3.707607268	-1.557390113	-3.376755243
H	-3.905541282	-1.093769077	-4.354407311
H	-2.837570205	-1.055218075	-2.932671209
H	-3.434179246	-2.608157188	-3.557526255
C	-4.209151305	-1.337915097	2.652304191
H	-4.393060317	-0.267144019	2.495215179
C	-5.192803374	-1.828971132	3.722595266
H	-5.131093369	-1.194450086	4.618739332
H	-6.233489443	-1.822058129	3.369760245
H	-4.960597355	-2.858111204	4.034674289
C	-2.766892201	-1.467413105	3.163084228
H	-2.660090193	-0.939947067	4.122490296
H	-2.495881180	-2.520579184	3.331852241
H	-2.037045145	-1.043696077	2.458820177

**3 (S_1)**			
B	2.308612000	-0.394723000	0.069522000
S	1.602302000	1.334888000	0.309764000
N	1.191336000	-1.289588000	-0.131104000

C	-0.023144000	-0.696304000	-0.114112000
H	1.267972000	-2.286939000	-0.299872000
C	3.795142000	-0.826492000	0.076719000
N	4.812149000	0.017576000	-0.002892000
C	6.163386000	-0.655165000	-0.064098000
C	5.797635000	-2.068741000	0.407357000
H	6.007353000	-2.159816000	1.483616000
H	6.409536000	-2.824036000	-0.101047000
C	4.282153000	-2.261435000	0.163468000
C	6.699590000	-0.631373000	-1.500610000
H	7.669101000	-1.147766000	-1.517463000
H	6.865246000	0.398547000	-1.842542000
H	6.038826000	-1.140567000	-2.212680000
C	7.195898000	0.016403000	0.836318000
H	8.158475000	-0.494333000	0.695995000
H	6.938587000	-0.053943000	1.899207000
H	7.338367000	1.072710000	0.567142000
C	3.984141000	-3.015016000	-1.162211000
H	2.902373000	-2.966896000	-1.378413000
H	4.475102000	-2.506245000	-2.004961000
C	4.401854000	-4.485652000	-1.106601000
H	4.111240000	-4.977362000	-2.046758000
H	5.500642000	-4.559298000	-1.055671000
C	3.786613000	-5.206055000	0.091594000
H	2.690437000	-5.264043000	-0.039353000
H	4.145148000	-6.244548000	0.134568000
C	4.098751000	-4.479749000	1.398803000
H	5.182799000	-4.519624000	1.598434000
H	3.614359000	-4.984431000	2.247609000
C	3.635080000	-3.024126000	1.343381000
H	3.863414000	-2.500511000	2.285566000
H	2.536148000	-3.015910000	1.248769000
C	4.625764000	1.440572000	-0.131682000
C	4.325559000	1.984762000	-1.402845000

C	4.131798000	3.368366000	-1.479961000
H	3.909508000	3.822406000	-2.447708000
C	4.211901000	4.174624000	-0.352616000
H	4.063892000	5.252574000	-0.440674000
C	4.468004000	3.608180000	0.891042000
H	4.500166000	4.249322000	1.774194000
C	4.673198000	2.233236000	1.036933000
C	4.874476000	1.666696000	2.437925000
H	5.085411000	0.592856000	2.344161000
C	6.060017000	2.324048000	3.155683000
H	6.272680000	1.798841000	4.098300000
H	5.842429000	3.372178000	3.410217000
H	6.972980000	2.314104000	2.545178000
C	3.603891000	1.789617000	3.289358000
H	3.787501000	1.393313000	4.298831000
H	2.762030000	1.229721000	2.856352000
H	3.290253000	2.839506000	3.396244000
C	4.181443000	1.158917000	-2.675425000
H	4.425332000	0.116090000	-2.439297000
C	5.149436000	1.629467000	-3.769205000
H	5.134923000	0.930282000	-4.618006000
H	6.183451000	1.702218000	-3.404965000
H	4.866496000	2.619801000	-4.156283000
C	2.741487000	1.164825000	-3.206281000
H	2.682213000	0.587475000	-4.140606000
H	2.393523000	2.186237000	-3.423476000
H	2.036390000	0.716598000	-2.490000000
B	-2.308561000	0.394629000	-0.069056000
S	-1.602330000	-1.334872000	-0.309252000
N	-1.191362000	1.289511000	0.131419000
C	0.023164000	0.696273000	0.114500000
H	-1.268000000	2.286872000	0.300143000
C	-3.795179000	0.826461000	-0.076069000
N	-4.812106000	-0.017517000	0.003865000

C	-6.163347000	0.655250000	0.065432000
C	-5.797722000	2.068715000	-0.406459000
H	-6.007810000	2.159558000	-1.482665000
H	-6.409419000	2.824144000	0.101986000
C	-4.282139000	2.261407000	-0.163132000
C	-6.698991000	0.631829000	1.502156000
H	-7.668349000	1.148498000	1.519269000
H	-6.864818000	-0.397969000	1.844362000
H	-6.037805000	1.140981000	2.213868000
C	-7.196130000	-0.016608000	-0.834445000
H	-8.158743000	0.493964000	-0.693776000
H	-6.939281000	0.053655000	-1.897450000
H	-7.338289000	-1.072913000	-0.565092000
C	-3.983615000	3.015594000	1.162080000
H	-2.901786000	2.967514000	1.377964000
H	-4.474339000	2.507290000	2.005245000
C	-4.401225000	4.486239000	1.105872000
H	-4.110256000	4.978385000	2.045689000
H	-5.500024000	4.559954000	1.055275000
C	-3.786326000	5.206005000	-0.092884000
H	-2.690103000	5.263979000	0.037670000
H	-4.144800000	6.244503000	-0.136248000
C	-4.098952000	4.479081000	-1.399632000
H	-5.183061000	4.518940000	-1.598931000
H	-3.614795000	4.983300000	-2.248847000
C	-3.635363000	3.023452000	-1.343643000
H	-3.864045000	2.499385000	-2.285493000
H	-2.536406000	3.015190000	-1.249359000
C	-4.625792000	-1.440648000	0.131721000
C	-4.325883000	-1.985766000	1.402502000
C	-4.132106000	-3.369420000	1.478592000
H	-3.910029000	-3.824206000	2.446037000
C	-4.211880000	-4.174796000	0.350583000
H	-4.063835000	-5.252807000	0.437833000

C	-4.467740000	-3.607407000	-0.892686000
H	-4.499731000	-4.247873000	-1.776335000
C	-4.672995000	-2.232360000	-1.037541000
C	-4.874381000	-1.664744000	-2.438084000
H	-5.085700000	-0.591042000	-2.343480000
C	-6.059743000	-2.321882000	-3.156352000
H	-6.272625000	-1.795942000	-4.098512000
H	-5.841842000	-3.369726000	-3.411785000
H	-6.972662000	-2.312735000	-2.545766000
C	-3.603756000	-1.786557000	-3.289604000
H	-3.787502000	-1.389558000	-4.298780000
H	-2.762106000	-1.226675000	-2.856169000
H	-3.289728000	-2.836246000	-3.397281000
C	-4.181904000	-1.160793000	2.675660000
H	-4.425762000	-0.117816000	2.440163000
C	-5.150063000	-1.632052000	3.768979000
H	-5.135688000	-0.933437000	4.618250000
H	-6.184022000	-1.704569000	3.404510000
H	-4.867198000	-2.622650000	4.155437000
C	-2.742001000	-1.167032000	3.206656000
H	-2.682838000	-0.590337000	4.141394000
H	-2.394031000	-2.188586000	3.423171000
H	-2.036847000	-0.718276000	2.490757000

3-Cl (S_0)

B	2.271043075	0.343714949	-0.105036980
S	1.580123017	-1.354620170	-0.259479991
N	1.231345007	1.275433023	0.071120033
C	0.006259916	0.682956983	0.083667034
H	1.209258011	2.349186097	0.193090042
C	3.798356187	0.740507967	-0.133117981
N	4.782108255	-0.115145097	-0.002853972
C	6.156346354	0.514226941	0.020662029
C	5.832996353	1.901004041	-0.535759013

H	6.018803344	1.910487040	-1.621175090
H	6.477858417	2.669089096	-0.092481979
C	4.329978235	2.151827066	-0.277862992
C	6.697203392	0.558164944	1.452399134
H	7.681191447	1.047166971	1.437369130
H	6.830635382	-0.455095132	1.853461164
H	6.051073367	1.127484986	2.130813184
C	7.158859423	-0.245019119	-0.841731034
H	8.136364474	0.248445912	-0.746429026
H	6.886603390	-0.236080117	-1.903131111
H	7.271718431	-1.286898193	-0.508720009
C	4.058406217	2.954662128	1.026664099
H	2.970490142	3.043976140	1.171461113
H	4.471717244	2.427127085	1.900815167
C	4.623584267	4.374413227	0.936137096
H	4.339870251	4.916885266	1.850873159
H	5.728574336	4.352283220	0.922500093
C	4.088618232	5.116799281	-0.289809993
H	3.007151155	5.274281298	-0.146899983
H	4.570272273	6.103884357	-0.373388999
C	4.291032244	4.317461224	-1.578097088
H	5.365556309	4.250611215	-1.830152102
H	3.809794210	4.838760262	-2.419654144
C	3.684848191	2.917922124	-1.454496079
H	3.818454196	2.344130084	-2.387376142
H	2.606079114	3.035020143	-1.274230065
C	4.586015233	-1.536687201	0.179173041
C	4.315548211	-2.032977232	1.472781132
C	4.097802188	-3.406176329	1.602422144
H	3.872296171	-3.819145359	2.587503215
C	4.114617186	-4.249832393	0.500347064
H	3.911948169	-5.314861446	0.624449072
C	4.350586207	-3.731482354	-0.765019029
H	4.322008201	-4.398017406	-1.628961087

C	4.583027228	-2.368187259	-0.961567043
C	4.759002244	-1.863092221	-2.390055144
H	4.968124266	-0.784809146	-2.351307140
C	5.937775301	-2.552849280	-3.090192195
H	6.138083336	-2.074299244	-4.060981262
H	5.712842320	-3.611855354	-3.288838211
H	6.859192379	-2.517292279	-2.492606149
C	3.482633154	-2.033177230	-3.225334206
H	3.657598166	-1.675113206	-4.251467275
H	2.639738094	-1.472881183	-2.802244173
H	3.178919127	-3.089536301	-3.282957210
C	4.168231208	-1.158078168	2.712607225
H	4.396407225	-0.121403096	2.435800205
C	5.143695276	-1.572893207	3.821258301
H	5.109197274	-0.849693151	4.650062365
H	6.181788350	-1.634580214	3.464358277
H	4.878888249	-2.558254276	4.234487332
C	2.729220104	-1.162411161	3.247892264
H	2.659023102	-0.515964115	4.135796326
H	2.413985074	-2.175537234	3.540735281
H	2.013495053	-0.795943131	2.501083209
B	-2.271056252	-0.343721078	0.105006035
S	-1.580132193	1.354618043	0.259435047
N	-1.231367184	-1.275455148	-0.071146977
C	-0.006290092	-0.682967112	-0.083671978
H	-1.209222186	-2.349283228	-0.193273986
C	-3.798359367	-0.740489097	0.133163038
N	-4.782126432	0.115148968	0.002868028
C	-6.156350535	-0.514250070	-0.020594973
C	-5.832975504	-1.901008174	0.535852069
H	-6.018768525	-1.910482171	1.621271146
H	-6.477815553	-2.669116221	0.092582035
C	-4.329960412	-2.151808196	0.277917048
C	-6.697250594	-0.558268070	-1.452318075

H	-7.681249656	-1.047243099	-1.437220077
H	-6.830669567	0.454961003	-1.853465106
H	-6.051168513	-1.127667113	-2.130710126
C	-7.158843600	0.245033989	0.841787090
H	-8.136348652	-0.248452041	0.746585081
H	-6.886519569	0.236206988	1.903170164
H	-7.271729596	1.286882064	0.508687065
C	-4.058364396	-2.954598256	-1.026617045
H	-2.970434319	-3.043831267	-1.171397056
H	-4.471706423	-2.427068214	-1.900755110
C	-4.623486443	-4.374373354	-0.936127037
H	-4.339762426	-4.916826395	-1.850872106
H	-5.728477549	-4.352240351	-0.922477037
C	-4.088509406	-5.116769414	0.289813049
H	-3.007038330	-5.274226428	0.146907039
H	-4.570141450	-6.103868481	0.373356055
C	-4.290964420	-4.317459351	1.578111143
H	-5.365498523	-4.250630345	1.830135158
H	-3.809748389	-4.838768396	2.419674200
C	-3.684799369	-2.917909254	1.454535132
H	-3.818388377	-2.344129216	2.387426202
H	-2.606038293	-3.034998272	1.274234119
C	-4.586055408	1.536691069	-0.179161985
C	-4.315558387	2.032981106	-1.472762076
C	-4.097802368	3.406182203	-1.602396087
H	-3.872242348	3.819145232	-2.587467156
C	-4.114659362	4.249836261	-0.500322008
H	-3.911958345	5.314859329	-0.624408018
C	-4.350667382	3.731480227	0.765035084
H	-4.322098378	4.398016273	1.628976147
C	-4.583111405	2.368187127	0.961574096
C	-4.759021421	1.863068096	2.390063198
H	-4.968209442	0.784801018	2.351308199
C	-5.937712487	2.552875152	3.090291251

H	-6.137966494	2.074333117	4.061095321
H	-5.712732462	3.611875225	3.288918265
H	-6.859177562	2.517350150	2.492776206
C	-3.482575331	2.033037098	3.225251262
H	-3.657481345	1.674901075	4.251370335
H	-2.639744270	1.472727055	2.802049229
H	-3.178793299	3.089372176	3.282940266
C	-4.168138385	1.158064039	-2.712559165
H	-4.396122406	0.121363967	-2.435693147
C	-5.143758449	1.572667073	-3.821159249
H	-5.109264453	0.849406025	-4.649910306
H	-6.181819518	1.634251086	-3.464141219
H	-4.879115425	2.558030143	-4.234488274
C	-2.729150280	1.162609031	-3.247896207
H	-2.658905275	0.516218986	-4.135838268
H	-2.414035250	2.175786106	-3.540684226
H	-2.013362230	0.796165004	-2.501134153
Cl	0.709898984	4.139314227	0.384742056
Cl	-0.710041158	-4.139229358	-0.385029000

**3-Cl (S_1)**			
B	2.278414000	0.353742000	-0.105593000
S	1.568503000	-1.381377000	-0.251014000
N	1.203261000	1.289169000	0.058461000
C	0.004645000	0.707217000	0.076575000
H	1.167020000	2.384022000	0.187154000
C	3.782415000	0.747790000	-0.141321000
N	4.789237000	-0.119693000	-0.021166000
C	6.147937000	0.518263000	-0.013593000
C	5.812672000	1.903662000	-0.573547000
H	5.980996000	1.904861000	-1.661981000
H	6.465787000	2.675079000	-0.146965000
C	4.312977000	2.158560000	-0.295249000
C	6.711877000	0.580341000	1.411523000

H	7.692278000	1.076676000	1.381469000
H	6.857086000	-0.428975000	1.819469000
H	6.069737000	1.149719000	2.093727000
C	7.154705000	-0.233967000	-0.880647000
H	8.131063000	0.263410000	-0.792302000
H	6.876118000	-0.230501000	-1.940759000
H	7.274412000	-1.274807000	-0.545922000
C	4.062616000	2.972889000	1.005874000
H	2.976857000	3.046283000	1.174999000
H	4.498775000	2.454371000	1.874015000
C	4.607925000	4.399448000	0.900217000
H	4.341309000	4.943566000	1.819389000
H	5.712334000	4.387842000	0.859030000
C	4.040054000	5.129447000	-0.317644000
H	2.957901000	5.268654000	-0.161342000
H	4.500374000	6.125874000	-0.410689000
C	4.243490000	4.328252000	-1.604165000
H	5.317826000	4.273031000	-1.859317000
H	3.752677000	4.840195000	-2.446244000
C	3.658112000	2.920873000	-1.469916000
H	3.793029000	2.345059000	-2.401132000
H	2.578008000	3.025802000	-1.289125000
C	4.585902000	-1.528153000	0.194759000
C	4.297352000	-1.995316000	1.496837000
C	4.057165000	-3.362040000	1.658275000
H	3.818343000	-3.748083000	2.651404000
C	4.072746000	-4.232931000	0.577420000
H	3.854042000	-5.291845000	0.725069000
C	4.327324000	-3.747577000	-0.697956000
H	4.295064000	-4.433702000	-1.546610000
C	4.578930000	-2.392143000	-0.923290000
C	4.759974000	-1.915140000	-2.359916000
H	4.980950000	-0.839400000	-2.334317000
C	5.928926000	-2.628826000	-3.050804000

H	6.127812000	-2.173786000	-4.033196000
H	5.697487000	-3.691125000	-3.223714000
H	6.853222000	-2.583340000	-2.458196000
C	3.478202000	-2.080278000	-3.187188000
H	3.654460000	-1.748930000	-4.222053000
H	2.648316000	-1.491427000	-2.775549000
H	3.150138000	-3.130590000	-3.218687000
C	4.168340000	-1.087821000	2.714406000
H	4.414663000	-0.064399000	2.407131000
C	5.139764000	-1.491294000	3.830394000
H	5.119297000	-0.748388000	4.642185000
H	6.175016000	-1.574972000	3.470020000
H	4.863002000	-2.463319000	4.267110000
C	2.731625000	-1.050509000	3.252359000
H	2.674499000	-0.384039000	4.126261000
H	2.391649000	-2.049783000	3.564966000
H	2.023185000	-0.681486000	2.499134000
B	-2.278331000	-0.353685000	0.104936000
S	-1.568443000	1.381453000	0.250160000
N	-1.203183000	-1.289089000	-0.059233000
C	-0.004571000	-0.707129000	-0.077437000
H	-1.166931000	-2.383938000	-0.187834000
C	-3.782309000	-0.747794000	0.141070000
N	-4.789199000	0.119640000	0.021204000
C	-6.147873000	-0.518385000	0.013961000
C	-5.812403000	-1.903754000	0.573859000
H	-5.980450000	-1.904936000	1.662336000
H	-6.465588000	-2.675212000	0.147460000
C	-4.312766000	-2.158586000	0.295190000
C	-6.712131000	-0.580554000	-1.411023000
H	-7.692461000	-1.077016000	-1.380733000
H	-6.857569000	0.428733000	-1.818957000
H	-6.070071000	-1.149864000	-2.093362000
C	-7.154475000	0.233816000	0.881230000

H	-8.130840000	-0.263583000	0.793093000
H	-6.875657000	0.230349000	1.941281000
H	-7.274279000	1.274658000	0.546542000
C	-4.062682000	-2.972991000	-1.005932000
H	-2.976957000	-3.046373000	-1.175292000
H	-4.499049000	-2.454539000	-1.874009000
C	-4.607935000	-4.399553000	-0.900054000
H	-4.341517000	-4.943727000	-1.819249000
H	-5.712335000	-4.387968000	-0.858617000
C	-4.039768000	-5.129458000	0.317726000
H	-2.957651000	-5.268670000	0.161181000
H	-4.500059000	-6.125882000	0.410948000
C	-4.242901000	-4.328180000	1.604243000
H	-5.317174000	-4.272972000	1.859664000
H	-3.751863000	-4.840056000	2.446230000
C	-3.657588000	-2.920797000	1.469758000
H	-3.792297000	-2.344923000	2.400967000
H	-2.577527000	-3.025721000	1.288710000
C	-4.586003000	1.528148000	-0.194549000
C	-4.297957000	1.995571000	-1.496641000
C	-4.057932000	3.362345000	-1.657912000
H	-3.819504000	3.748591000	-2.651057000
C	-4.073188000	4.233032000	-0.576888000
H	-3.854628000	5.291992000	-0.724418000
C	-4.327264000	3.747420000	0.698491000
H	-4.294753000	4.433389000	1.547262000
C	-4.578696000	2.391927000	0.923661000
C	-4.759247000	1.914643000	2.360255000
H	-4.980182000	0.838895000	2.334523000
C	-5.928014000	2.628145000	3.051650000
H	-6.126574000	2.172901000	4.034014000
H	-5.696561000	3.690418000	3.224701000
H	-6.852492000	2.582743000	2.459319000
C	-3.477219000	2.079691000	3.187146000

H	-3.653121000	1.748118000	4.221999000
H	-2.647428000	1.490984000	2.775110000
H	-3.149212000	3.130017000	3.218752000
C	-4.169315000	1.088309000	-2.714418000
H	-4.415362000	0.064798000	-2.407214000
C	-5.141253000	1.491843000	-3.829938000
H	-5.121049000	0.749062000	-4.641850000
H	-6.176363000	1.575367000	-3.469123000
H	-4.864772000	2.463962000	-4.266623000
C	-2.732819000	1.051306000	-3.252974000
H	-2.675945000	0.384965000	-4.126991000
H	-2.393143000	2.050679000	-3.565590000
H	-2.024008000	0.682308000	-2.500089000
Cl	0.633545000	4.093239000	0.400352000
Cl	-0.633413000	-4.093201000	-0.400864000

3-OTf·HOTf

H	2.408453000	1.212120000	-0.873787000
B	2.125609000	-0.970978000	-0.575695000
S	0.652330000	-1.893274000	0.001507000
N	1.814161000	0.404075000	-0.658910000
C	0.523836000	0.681857000	-0.287721000
C	3.557343000	-1.581702000	-0.882786000
N	3.998965000	-2.692412000	-0.358449000
C	5.462494000	-2.977462000	-0.643503000
C	5.867888000	-1.709908000	-1.410684000
H	6.528825000	-1.954887000	-2.251177000
H	6.415206000	-1.034018000	-0.743050000
C	4.568770000	-1.013998000	-1.859013000
C	6.240955000	-3.132956000	0.661676000
H	6.201208000	-2.226849000	1.274164000
H	7.294260000	-3.320728000	0.409646000
H	5.874629000	-3.987414000	1.248389000
C	5.644345000	-4.252754000	-1.466265000

H	5.218852000	-5.122737000	-0.948890000
H	6.722272000	-4.428682000	-1.585235000
H	5.208775000	-4.182618000	-2.470331000
C	3.159460000	-3.606297000	0.400393000
C	3.025899000	-3.432640000	1.794077000
C	2.158568000	-4.292379000	2.472312000
H	2.031783000	-4.170419000	3.549708000
C	1.434435000	-5.269167000	1.804267000
H	0.745302000	-5.913582000	2.353127000
C	1.570047000	-5.409909000	0.430385000
H	0.975136000	-6.161689000	-0.090818000
C	2.424282000	-4.586225000	-0.306738000
C	2.469598000	-4.781302000	-1.818478000
H	3.221974000	-4.099913000	-2.227593000
C	2.881786000	-6.210728000	-2.193403000
H	2.088775000	-6.930655000	-1.940013000
H	3.797059000	-6.532223000	-1.677143000
H	3.055965000	-6.284760000	-3.277680000
C	1.141258000	-4.425935000	-2.495718000
H	0.317457000	-5.044366000	-2.111556000
H	1.215770000	-4.598382000	-3.580378000
H	0.863429000	-3.376900000	-2.333667000
C	3.720515000	-2.334378000	2.580236000
H	4.401386000	-1.807588000	1.909329000
C	4.549734000	-2.895292000	3.740728000
H	5.172746000	-2.094916000	4.164647000
H	5.215108000	-3.707680000	3.413440000
H	3.908574000	-3.292350000	4.543571000
C	2.737633000	-1.270802000	3.086160000
H	2.209862000	-0.773836000	2.260604000
H	3.292424000	-0.498727000	3.640062000
H	1.982538000	-1.701353000	3.762828000
C	4.095675000	-1.466243000	-3.274522000
H	3.987286000	-2.560289000	-3.321696000

H	3.090786000	-1.045935000	-3.451322000
C	5.046831000	-0.991241000	-4.376411000
H	6.008529000	-1.524852000	-4.287480000
H	4.629305000	-1.283082000	-5.352624000
C	5.294383000	0.516760000	-4.327248000
H	6.045051000	0.799569000	-5.081228000
H	4.363168000	1.044147000	-4.598718000
C	5.723159000	0.968545000	-2.932518000
H	5.802656000	2.063437000	-2.877102000
H	6.724216000	0.572189000	-2.690038000
C	4.705118000	0.515578000	-1.890875000
H	3.743935000	0.962774000	-2.178633000
H	4.967649000	0.890774000	-0.896651000
B	-1.818673000	1.250435000	0.365636000
S	-0.361151000	2.176387000	-0.223546000
N	-1.510720000	-0.120013000	0.466732000
H	-2.104879000	-0.925899000	0.708515000
C	-0.223502000	-0.399676000	0.080151000
C	-3.230018000	1.879847000	0.718009000
N	-3.820584000	2.792475000	0.003990000
C	-5.200087000	3.181823000	0.516969000
C	-5.417370000	2.130814000	1.616169000
H	-5.889291000	2.582881000	2.497199000
H	-6.083854000	1.342918000	1.245807000
C	-4.039797000	1.518567000	1.939784000
C	-6.216360000	3.079464000	-0.614813000
H	-6.270467000	2.059637000	-1.010266000
H	-7.208086000	3.327367000	-0.210116000
H	-5.990398000	3.787485000	-1.425324000
C	-5.225608000	4.594867000	1.097010000
H	-5.062263000	5.358224000	0.329948000
H	-6.227489000	4.759948000	1.517487000
H	-4.497410000	4.731694000	1.907866000
C	-3.322166000	3.250779000	-1.282968000

C	-3.461777000	2.376411000	-2.386136000
C	-3.031036000	2.842002000	-3.630981000
H	-3.129144000	2.189221000	-4.500385000
C	-2.459738000	4.097196000	-3.782121000
H	-2.125699000	4.433027000	-4.765738000
C	-2.286848000	4.911966000	-2.673123000
H	-1.799157000	5.881330000	-2.792073000
C	-2.705914000	4.514607000	-1.400172000
C	-2.418495000	5.460887000	-0.240180000
H	-2.810182000	5.007305000	0.678707000
C	-3.100329000	6.822325000	-0.443942000
H	-2.583134000	7.403658000	-1.222531000
H	-4.150801000	6.731634000	-0.755087000
H	-3.063944000	7.413473000	0.483569000
C	-0.915801000	5.673254000	-0.019684000
H	-0.432298000	6.112286000	-0.905552000
H	-0.754081000	6.364108000	0.821436000
H	-0.391042000	4.739445000	0.208854000
C	-3.953467000	0.935620000	-2.305504000
H	-4.270618000	0.704124000	-1.279361000
C	-5.166435000	0.668694000	-3.203184000
H	-5.503132000	-0.368621000	-3.064889000
H	-6.011256000	1.331036000	-2.970641000
H	-4.917264000	0.799023000	-4.267749000
C	-2.813851000	-0.031682000	-2.667803000
H	-1.870514000	0.234292000	-2.171151000
H	-3.081171000	-1.052769000	-2.364810000
H	-2.624636000	-0.025912000	-3.752824000
C	-3.342452000	2.167478000	3.167521000
H	-3.261175000	3.257816000	3.030876000
H	-2.310219000	1.781117000	3.224992000
C	-4.074990000	1.846882000	4.471888000
H	-5.057495000	2.348982000	4.477775000
H	-3.509816000	2.275537000	5.313735000

C	-4.270561000	0.343106000	4.668144000
H	-4.860746000	0.155537000	5.578179000
H	-3.286385000	-0.130559000	4.829520000
C	-4.933071000	-0.302419000	3.451745000
H	-4.991899000	-1.393224000	3.577719000
H	-5.972813000	0.052202000	3.348350000
C	-4.140077000	-0.000085000	2.182703000
H	-3.135210000	-0.422200000	2.319971000
H	-4.590195000	-0.491585000	1.308571000
C	3.229466000	5.247361000	0.144079000
F	4.153313000	5.837102000	0.885776000
F	3.066732000	5.937496000	-0.974485000
F	2.084523000	5.246931000	0.814712000
S	3.751079000	3.514548000	-0.246845000
O	5.102614000	3.622212000	-0.766695000
O	3.653469000	2.851859000	1.097516000
O	2.707121000	3.018182000	-1.152017000
C	-2.590153000	-4.256049000	-0.955248000
F	-3.440187000	-4.927642000	-1.716477000
F	-1.766377000	-5.117724000	-0.370846000
F	-1.873325000	-3.449712000	-1.732272000
S	-3.508985000	-3.269135000	0.315131000
O	-4.367895000	-4.209900000	1.001990000
O	-4.221587000	-2.272678000	-0.551097000
O	-2.444552000	-2.622346000	1.095161000
F	8.433334000	1.719683000	2.119776000
O	-6.442564000	-0.090065000	-0.346823000
C	7.612245000	1.080883000	1.312017000
F	8.163030000	-0.079531000	0.960548000
F	7.408384000	1.799449000	0.224749000
O	6.341094000	-0.006593000	3.356858000
O	5.181855000	0.086613000	1.136890000
O	5.501710000	2.136510000	2.480479000
H	4.701225000	2.489903000	1.819273000

S	6.002230000	0.726356000	2.159477000
C	-7.840733000	-1.877755000	0.968691000
F	-8.359603000	-0.898674000	1.699511000
F	-8.734346000	-2.833115000	0.821388000
F	-6.776357000	-2.357554000	1.587564000
O	-8.557796000	-0.934012000	-1.415636000
O	-6.573413000	-2.367287000	-1.301163000
H	-5.575065000	-2.388081000	-0.977126000
S	-7.347931000	-1.180380000	-0.678355000

3-BAr$^{F}_{4}$·Et_2O (partially optimized)

C	11.114479000	0.628441000	-1.372480000
C	9.888927000	1.143901000	-1.816117000
H	9.131634000	1.083088000	-1.245779000
C	9.744870000	1.741459000	-3.063669000
C	10.821159000	1.853212000	-3.927362000
H	10.728390000	2.274454000	-4.773800000
C	12.037323000	1.331631000	-3.518619000
C	12.176167000	0.743229000	-2.269217000
H	13.027129000	0.405766000	-2.015247000
C	8.411900000	2.304683000	-3.448761000
C	13.239474000	1.374963000	-4.420512000
C	10.925454000	-1.758267000	-0.435837000
C	11.903864000	-2.563195000	-1.024797000
H	12.798354000	-2.245566000	-1.063463000
C	11.611777000	-3.808970000	-1.553929000
C	10.321974000	-4.322897000	-1.522871000
H	10.126577000	-5.180838000	-1.881006000
C	9.335413000	-3.546798000	-0.955289000
C	9.637015000	-2.291363000	-0.426658000
H	8.934967000	-1.778072000	-0.044333000
C	12.673013000	-4.634112000	-2.190804000
C	7.916873000	-4.035239000	-0.961437000
C	10.277683000	0.288362000	1.224101000

C	9.936798000	-0.586902000	2.265501000
H	10.182634000	-1.501914000	2.196009000
C	9.254517000	-0.161048000	3.390030000
C	8.861065000	1.157139000	3.529546000
H	8.383116000	1.445068000	4.298423000
C	9.182027000	2.043729000	2.520311000
C	9.885012000	1.622043000	1.391775000
H	10.103190000	2.256411000	0.719098000
C	8.927933000	-1.164678000	4.457232000
C	8.819908000	3.488696000	2.657998000
C	12.760102000	-0.052701000	0.666746000
C	13.435084000	1.166813000	0.610405000
H	13.040442000	1.887620000	0.133742000
C	14.663781000	1.363872000	1.226818000
C	15.271492000	0.348432000	1.939066000
H	16.120653000	0.475492000	2.345592000
C	14.602347000	-0.864086000	2.042339000
C	13.374977000	-1.056600000	1.420483000
H	12.940148000	-1.896460000	1.510294000
C	15.302320000	2.721076000	1.126786000
B	11.257826000	-0.218534000	0.020293000
F	7.372322000	1.570352000	-3.012015000
F	8.181716000	3.523279000	-2.893048000
F	8.239868000	2.480699000	-4.746156000
F	13.001884000	1.988327000	-5.579425000
F	14.278520000	1.992586000	-3.851857000
F	13.677024000	0.145043000	-4.721405000
F	13.831612000	-4.008920000	-2.339064000
F	12.904472000	-5.780090000	-1.558851000
F	12.305413000	-5.018282000	-3.449772000
F	7.170206000	-3.396345000	-1.871296000
F	7.805912000	-5.331376000	-1.237678000
F	7.296107000	-3.844230000	0.197595000
F	7.866271000	-1.911865000	4.151322000

F	8.703319000	-0.618799000	5.641538000
F	9.922098000	-2.058690000	4.654791000
F	7.793649000	3.681082000	3.501939000
F	8.456686000	4.046800000	1.495958000
F	9.831773000	4.236570000	3.121829000
F	16.537341000	2.755070000	1.615486000
F	14.606034000	3.659368000	1.759447000
F	15.382833000	3.154773000	-0.130831000
C	15.214391000	-1.939839000	2.884464000
F	14.543856000	-3.110643000	2.828254000
F	15.244102000	-1.631068000	4.181870000
F	16.461056000	-2.216655000	2.538310000
C	-11.123092000	-0.616685000	1.435279000
C	-9.897541000	-1.132145000	1.878916000
H	-9.140247000	-1.071331000	1.308578000
C	-9.753483000	-1.729702000	3.126468000
C	-10.829772000	-1.841456000	3.990161000
H	-10.737003000	-2.262698000	4.836599000
C	-12.045936000	-1.319874000	3.581418000
C	-12.184780000	-0.731473000	2.332016000
H	-13.035742000	-0.394010000	2.078046000
C	-8.420514000	-2.292927000	3.511560000
C	-13.248087000	-1.363207000	4.483311000
C	-10.934068000	1.770023000	0.498636000
C	-11.912477000	2.574951000	1.087596000
H	-12.806967000	2.257323000	1.126262000
C	-11.620390000	3.820727000	1.616728000
C	-10.330587000	4.334654000	1.585670000
H	-10.135191000	5.192594000	1.943805000
C	-9.344026000	3.558554000	1.018088000
C	-9.645628000	2.303119000	0.489457000
H	-8.943580000	1.789828000	0.107132000
C	-12.681626000	4.645868000	2.253603000
C	-7.925486000	4.046995000	1.024236000

C	-10.286297000	-0.276606000	-1.161302000
C	-9.945412000	0.598659000	-2.202702000
H	-10.191248000	1.513670000	-2.133210000
C	-9.263130000	0.172804000	-3.327231000
C	-8.869678000	-1.145383000	-3.466747000
H	-8.391730000	-1.433312000	-4.235624000
C	-9.190640000	-2.031972000	-2.457512000
C	-9.893625000	-1.610287000	-1.328976000
H	-10.111804000	-2.244655000	-0.656299000
C	-8.936546000	1.176434000	-4.394433000
C	-8.828521000	-3.476939000	-2.595199000
C	-12.768715000	0.064457000	-0.603947000
C	-13.443698000	-1.155057000	-0.547606000
H	-13.049055000	-1.875864000	-0.070943000
C	-14.672394000	-1.352116000	-1.164019000
C	-15.280105000	-0.336676000	-1.876267000
H	-16.129267000	-0.463736000	-2.282793000
C	-14.610960000	0.875842000	-1.979540000
C	-13.383590000	1.068356000	-1.357684000
H	-12.948761000	1.908217000	-1.447494000
C	-15.310933000	-2.709320000	-1.063987000
B	-11.266439000	0.230291000	0.042506000
F	-7.380935000	-1.558596000	3.074815000
F	-8.190329000	-3.511523000	2.955847000
F	-8.248481000	-2.468942000	4.808955000
F	-13.010497000	-1.976571000	5.642224000
F	-14.287133000	-1.980829000	3.914656000
F	-13.685637000	-0.133287000	4.784204000
F	-13.840225000	4.020676000	2.401863000
F	-12.913085000	5.791846000	1.621650000
F	-12.314027000	5.030038000	3.512571000
F	-7.178819000	3.408101000	1.934095000
F	-7.814525000	5.343132000	1.300477000
F	-7.304720000	3.855987000	-0.134796000

F	-7.874884000	1.923621000	-4.088523000
F	-8.711932000	0.630555000	-5.578739000
F	-9.930711000	2.070446000	-4.591992000
F	-7.802262000	-3.669326000	-3.439140000
F	-8.465300000	-4.035044000	-1.433159000
F	-9.840386000	-4.224814000	-3.059030000
F	-16.545954000	-2.743313000	-1.552687000
F	-14.614647000	-3.647612000	-1.696648000
F	-15.391446000	-3.143017000	0.193630000
C	-15.223004000	1.951595000	-2.821665000
F	-14.552469000	3.122400000	-2.765455000
F	-15.252716000	1.642825000	-4.119071000
F	-16.469669000	2.228412000	-2.475511000
C	3.307942000	2.053915000	0.238368000
C	0.451806000	-0.499153000	0.036871000
C	4.935643000	3.757287000	-0.038293000
C	5.116537000	5.079005000	0.696719000
H	4.848803000	5.012123000	1.757585000
H	4.538692000	5.885476000	0.223986000
H	6.178416000	5.356159000	0.642157000
C	5.447124000	3.928625000	-1.469804000
H	5.398124000	3.004532000	-2.058273000
H	6.500007000	4.238179000	-1.423001000
H	4.892623000	4.716915000	-1.995578000
C	5.585055000	2.577262000	0.696903000
H	5.722092000	2.832203000	1.758259000
H	6.580452000	2.359600000	0.289851000
C	4.618805000	1.380856000	0.572480000
C	4.524837000	0.579435000	1.888160000
H	4.292733000	1.265943000	2.718502000
H	3.687586000	-0.129823000	1.827609000
C	5.806013000	-0.204054000	2.169391000
H	6.645027000	0.491197000	2.343385000
H	5.684506000	-0.769553000	3.106048000

C	6.141372000	-1.143075000	1.011508000
H	5.337083000	-1.891457000	0.903812000
H	7.069451000	-1.694441000	1.220618000
C	6.266958000	-0.372305000	-0.300130000
H	6.454594000	-1.060680000	-1.138242000
H	7.141006000	0.299349000	-0.258886000
C	5.000094000	0.428764000	-0.596168000
H	4.177647000	-0.278185000	-0.771536000
H	5.115298000	1.003107000	-1.527862000
C	2.403155000	4.180327000	-0.491907000
C	1.764908000	4.995660000	0.468745000
C	0.731171000	5.822461000	0.018947000
H	0.225323000	6.477085000	0.731271000
C	0.335806000	5.829433000	-1.312153000
H	-0.463353000	6.497235000	-1.640207000
C	0.952195000	4.983208000	-2.225164000
H	0.616432000	4.980465000	-3.263912000
C	1.990876000	4.131281000	-1.842747000
C	2.101762000	5.006014000	1.956694000
H	2.958032000	4.337149000	2.122437000
C	0.947155000	4.462946000	2.811084000
H	1.221604000	4.497615000	3.875778000
H	0.696286000	3.424677000	2.557389000
H	0.038264000	5.072141000	2.685828000
C	2.489516000	6.408978000	2.442568000
H	2.871326000	6.362414000	3.473077000
H	1.618394000	7.081292000	2.450282000
H	3.259404000	6.875237000	1.813841000
C	2.565695000	3.181154000	-2.888568000
H	3.397230000	2.625702000	-2.436038000
C	3.116790000	3.936328000	-4.105015000
H	3.645251000	3.243879000	-4.776716000
H	3.815305000	4.735190000	-3.821585000
H	2.306532000	4.399966000	-4.687701000

C	1.535614000	2.137171000	-3.344097000
H	1.986867000	1.468170000	-4.091862000
H	0.662392000	2.615360000	-3.813872000
H	1.171196000	1.518809000	-2.511639000
B	1.911772000	1.301497000	0.190776000
N	3.479028000	3.306975000	-0.062123000
N	1.763972000	-0.103538000	0.120857000
H	2.454034000	-0.863469000	0.136409000
S	0.258072000	2.086528000	0.175849000
C	-3.313786000	-2.041855000	-0.244082000
C	-0.463805000	0.511765000	0.061276000
C	-4.935496000	-3.770647000	-0.135971000
C	-5.113702000	-5.011943000	-1.000765000
H	-4.861258000	-4.831768000	-2.052241000
H	-4.519855000	-5.856711000	-0.623812000
H	-6.171428000	-5.306747000	-0.961527000
C	-5.437470000	-4.082695000	1.274716000
H	-5.370072000	-3.222990000	1.952824000
H	-6.494968000	-4.371797000	1.206120000
H	-4.890288000	-4.927225000	1.712538000
C	-5.596822000	-2.524313000	-0.739886000
H	-5.758317000	-2.676228000	-1.817360000
H	-6.582889000	-2.346741000	-0.292123000
C	-4.626659000	-1.344749000	-0.519573000
C	-4.523046000	-0.454911000	-1.778545000
H	-4.312146000	-1.088481000	-2.655515000
H	-3.667233000	0.224893000	-1.676835000
C	-5.779158000	0.385528000	-1.998037000
H	-6.642113000	-0.267195000	-2.215869000
H	-5.639678000	1.012298000	-2.891911000
C	-6.078176000	1.247714000	-0.773325000
H	-5.238232000	1.944694000	-0.603129000
H	-6.978963000	1.856051000	-0.939379000
C	-6.245808000	0.377653000	0.470284000

H	-6.426056000	0.999611000	1.360165000
H	-7.138528000	-0.261336000	0.361438000
C	-5.007035000	-0.483427000	0.714933000
H	-4.167262000	0.181253000	0.961234000
H	-5.152241000	-1.132110000	1.592169000
C	-2.394810000	-4.244561000	0.190726000
C	-1.777824000	-4.885318000	-0.907566000
C	-0.739085000	-5.776938000	-0.626806000
H	-0.247715000	-6.298858000	-1.450175000
C	-0.321418000	-6.014627000	0.676252000
H	0.482567000	-6.727430000	0.869662000
C	-0.916619000	-5.335829000	1.731397000
H	-0.561272000	-5.511065000	2.748682000
C	-1.955449000	-4.425056000	1.520485000
C	-2.143633000	-4.638088000	-2.367852000
H	-3.010476000	-3.962856000	-2.399913000
C	-1.009432000	-3.936642000	-3.128881000
H	-1.299951000	-3.784837000	-4.179009000
H	-0.763593000	-2.957634000	-2.696414000
H	-0.092517000	-4.546692000	-3.125288000
C	-2.528303000	-5.938444000	-3.086132000
H	-2.936354000	-5.716164000	-4.083139000
H	-1.651365000	-6.586725000	-3.233081000
H	-3.277423000	-6.518932000	-2.531176000
C	-2.494428000	-3.656348000	2.721614000
H	-3.326493000	-3.024087000	2.385332000
C	-3.026966000	-4.597590000	3.810373000
H	-3.546965000	-4.023057000	4.590921000
H	-3.726707000	-5.346400000	3.414626000
H	-2.206677000	-5.143369000	4.300666000
C	-1.437366000	-2.714393000	3.316517000
H	-1.855852000	-2.182068000	4.183633000
H	-0.555511000	-3.272979000	3.666166000
H	-1.092135000	-1.965932000	2.589966000

B	-1.919849000	-1.291006000	-0.132653000
N	-3.479890000	-3.320042000	-0.077028000
N	-1.774190000	0.113320000	-0.032386000
H	-2.469898000	0.870006000	-0.047263000
S	-0.264999000	-2.072691000	-0.100881000
C	2.653487000	-2.677003000	2.499688000
H	2.440140000	-3.324697000	3.361690000
H	1.865432000	-1.908962000	2.454322000
H	3.619883000	-2.187135000	2.684556000
C	2.684457000	-3.504485000	1.235699000
H	3.485212000	-4.265383000	1.286536000
H	1.726316000	-4.043452000	1.109159000
C	2.858795000	-3.399708000	-1.111427000
H	1.891716000	-3.935683000	-1.180065000
H	3.656489000	-4.164954000	-1.107971000
C	3.023070000	-2.459784000	-2.284172000
H	2.930464000	-3.018354000	-3.225957000
H	4.012050000	-1.979181000	-2.277209000
H	2.246722000	-1.678586000	-2.279181000
O	2.899118000	-2.676187000	0.105472000
C	-2.433042000	2.741498000	-2.370742000
H	-2.342954000	3.433978000	-3.219924000
H	-3.208072000	2.006586000	-2.629359000
H	-1.469921000	2.223107000	-2.252971000
C	-2.790505000	3.515997000	-1.122478000
H	-2.025740000	4.292238000	-0.933167000
H	-3.762257000	4.029991000	-1.253560000
C	-2.962061000	3.362633000	1.226287000
H	-3.911910000	3.928254000	1.239237000
H	-2.140167000	4.101364000	1.286191000
C	-2.888232000	2.410404000	2.398240000
H	-2.934878000	2.976656000	3.338948000
H	-1.944005000	1.844716000	2.392078000
H	-3.728245000	1.701352000	2.398541000

O	-2.850457000	2.657277000	0.002172000

References

[1] S. Hagspiel, M. Arrowsmith, F. Fantuzzi, A. Vargas, A. Rempel, A. Hermann, T. Brückner, H. Braunschweig, *Angew. Chem. Int. Ed.* **2021**, *60*, 6446–6450.

[2] M. Brookhart, B. Grant, A. F. Volpe, *Organometallics* **1992**, *11*, 3920–3922.

[3] G. Sheldrick, *Acta Cryst.* **2015**, *A71*, 3–8.

[4] G. Sheldrick, *Acta Cryst.* **2008**, *A64*, 112–122.

[5] J.-D. Chai, M. Head-Gordon, *J. Chem. Phys*. **2008**, *128*, 084106.

[6] F. Weigend, R. Ahlrichs, *Phys. Chem. Chem. Phys.* **2005**, *7*, 3297–3305.

[7] T. Lu, F. Chen, *J. Comput. Chem*. **2012**, *33*, 580–592.

[8] W. Qiao, G. Duan, J. Wang, J. Dong, B. Pan, X. Mu, *ACS Omega* **2020**, *5*, 17275–17286.

[9] Y. Cui, P. Zhu, X. Shi, X. Liao, Y. Chen, *J. Phys. Chem. C* **2021**, *125*, 10250–10259.

[10] M. J. G. Peach, P. Benfield, T. Helgaker, D. J. Tozer, *J. Chem. Phys.* **2008**, *128*, 044118.

[11] Z. Chen, C. S. Wannere, C. Corminboeuf, R. Puchta, P. von R. Schleyer, *Chem. Rev*. **2005**, *105*, 3842–3888.

[12] A. Stanger, *J. Org. Chem*. **2006**, *71*, 883–893.

[13] J. O. C. Jiménez-Halla, E. Matito, J. Robles, M. Solà, *J. Organomet. Chem*. **2006**, *691*, 4359–4366.

[14] A. C. Tsipis, *Phys. Chem. Chem. Phys*. **2009**, *11*, 8244.

[15] R. Ditchfield, *Mol. Phys.* **1974**, *27*, 789–807.

[16] K. Wolinski, J. F. Hinton, P. Pulay, *J. Am. Chem. Soc.* **1990**, *112*, 8251–8260.

[17] J. R. Cheeseman, G. W. Trucks, T. A. Keith, M. J. Frisch, *J. Chem. Phys.* **1996**, *104*, 5497–5509.

[18] D. Geuenich, K. Hess, F. Köhler, R. Herges, *Chem. Rev*. **2005**, *105*, 3758–3772.

[19] M. J. Frisch, G. W. Trucks, H. B. Schlegel, G. E. Scuseria, M. A. Robb, J. R. Cheeseman, G. Scalmani, V. Barone, G. A. Petersson, H. Nakatsuji, X. Li, M. Caricato, A. V. Marenich, J. Bloino, B. G. Janesko, R. Gomperts, B. Mennucci, H. P. Hratchian, J. V. Ortiz, A. F. Izmaylov, J. L. Sonnenberg, D. Williams-Young, F. Ding, F. Lipparini, F. Egidi, J. Goings, B. Peng, A. Petrone, T. Henderson, D. Ranasinghe, V. G. Zakrzewski, J. Gao, N. Rega, G. Zheng, W. Liang, M. Hada, M. Ehara, K. Toyota, R. Fukuda, J. Hasegawa, M. Ishida, T. Nakajima, Y. Honda, O. Kitao, H. Nakai, T. Vreven, K. Throssell, J. A. Montgomery, Jr., J. E. Peralta, F. Ogliaro, M. J. Bearpark, J. J. Heyd, E. N. Brothers, K. N. Kudin, V. N. Staroverov, T. A. Keith, R. Kobayashi, J. Normand,

K. Raghavachari, A. P. Rendell, J. C. Burant, S. S. Iyengar, J. Tomasi, M. Cossi, J. M. Millam, M. Klene, C. Adamo, R. Cammi, J. W. Ochterski, R. L. Martin, K. Morokuma, O. Farkas, J. B. Foresman, D. J. Fox, *Gaussian 16, Revision C.01* Gaussian, Inc.: Wallingford CT, **2016**.
